# Cholesterol maintains the degradative capacity of lysosomes during clearance and recycling of dysfunctional mitochondria

**DOI:** 10.1038/s41467-026-77423-1

**Published:** 2026-09-16

**Authors:** Haoning Yang, Koji Matsuhisa, Meng Wei, Wataru Nishi, Dylan Hong Zheng Koh, Aik Yong Sim, Isabelle Bonne, Yasunori Saheki

**Affiliations:** 1 Lee Kong Chian School of Medicine, Nanyang Technological University, Singapore, 308232 Singapore; 2 Electron Microscopy Unit (EMU), Microscopy Cluster, Yong Loo Lin School of Medicine, National University of Singapore, Singapore, 117549 Singapore; 3 Department of Microbiology & Immunology, Yong Loo Lin School of Medicine, National University of Singapore, Singapore, 117545 Singapore; 4 Institute of Resource Development and Analysis, Kumamoto University, Kumamoto, 860-0811 Japan; 5 Present Address: Department of Pharmacology and Therapeutic Innovation, Nagasaki University Graduate School of Biomedical Sciences, Nagasaki, 852-8521 Japan

**Keywords:** Lysosomes, Sterols, Mitochondria, Endoplasmic reticulum

## Abstract

Efficient clearance and recycling of dysfunctional mitochondria through the robust catabolic activity of lysosomes are essential for cellular health. However, how membrane lipids contribute to maintaining the degradative capacity of lysosomes remains poorly understood. Here, we show that cholesterol plays a critical role in preserving the functional integrity of degradative lysosomes. Clearance of damaged mitochondria by degradative lysosomes is tightly coupled with the acute accumulation of phosphatidylinositol 4-phosphate (PI4P) on the lysosomal surface via PI4KIIα activity. This PI4P accumulation activates oxysterol-binding protein (OSBP)-mediated cholesterol transport from the endoplasmic reticulum (ER) to lysosomal membranes. The resulting efflux of cholesterol from the ER activates sterol regulatory element-binding protein 2 (SREBP-2), enhancing cholesterol production. Sustained cholesterol accumulation on lysosomal membranes maintains lysosomal acidity and membrane integrity for efficient mitochondrial degradation. This degradation process then leads to the release of free fatty acids and their recycling and storage through the formation of DGAT1-dependent lipid droplets. These findings uncover a key phosphoinositide-regulated cholesterol transport pathway that promotes the clearance and recycling of dysfunctional mitochondria, a process whose impairment is closely linked to neurodegeneration.

## Abstract (web summary)

Damaged mitochondria are cleared by lysosomes. Here, authors show that ER-to-lysosome cholesterol transport preserves lysosomal degradative function during mitophagy, enabling efficient recycling of lipids and their storage in lipid droplets.

## Introduction

Mitochondria are essential for cellular energy homeostasis, playing a key role in maintaining cell physiology. The accumulation of dysfunctional or damaged mitochondria is deleterious to cells and is often associated with neurodegenerative diseases, including Alzheimer’s disease (AD) and Parkinson’s disease (PD)^1–3^. Hence, dysfunctional mitochondria are rapidly cleared and recycled by a selective degradation process termed mitophagy (i.e., mitochondrial degradation via the autophagy pathway). During this process, dysfunctional mitochondria are engulfed by autophagosomes, which subsequently fuse with lysosomes for degradation^4,5^.

Characterized by its acidic environment and abundance of digestive enzymes, the lysosome plays a crucial role in many catabolic processes, including autophagy^6–9^. During autophagy, lysosomes digest diverse cellular substrates, including damaged mitochondria, and facilitate the efflux of various nutrients for their metabolic recycling^6,10–13^. Disrupting the degradative property of the lysosome results in the accumulation of toxic waste, which leads to cellular damage or even death^10,14,15^. Thus, the functional integrity of lysosomes must be tightly regulated. However, lysosomes exhibit functional heterogeneity and vary in their digestive properties^16^. This heterogeneity is regulated, at least in part, by the lipid composition of the lysosomal membrane^17^. Phosphatidylinositol 4-phosphate (PI4P), generated from phosphatidylinositol (PI) by phosphatidylinositol 4-kinase IIα (PI4KIIα), promotes autophagy during nutrient starvation^18^. In contrast, phosphatidylinositol 3-phosphate enables lysosomes to coordinate with mTOR kinase to support anabolism and cell growth^18,19^. Lysosomal membranes contain relatively low levels of cholesterol at steady state^20^, but cholesterol biosynthesis and uptake are enhanced when lysosomal function is impaired via viral infection or chronic depletion of key lysosomal enzymes^21,22^. This suggests a potential requirement of cholesterol in supporting the functional integrity of lysosomes. However, it remains unclear how the regulatory network that drives cholesterol biosynthesis, uptake, and transport helps shape the degradative properties of lysosomes, including properties essential for the clearance and recycling of dysfunctional mitochondria.

Cholesterol plays an essential role in the structural integrity of cellular membranes^23^. It is highly enriched in the plasma membrane (PM) at steady state, primarily through non-vesicular transport mediated by lipid transfer proteins (LTPs)^24–27^, including oxysterol-binding protein (OSBP), whose functions are tightly regulated by PI4P^28–31^. In addition to regulating cholesterol transport, cells employ intricate strategies to balance cholesterol uptake, synthesis, and storage. Cholesterol is acquired either from the extracellular environment, primarily in the form of low-density lipoprotein (LDL), or synthesized de novo in the endoplasmic reticulum (ER)^32–34^. LDL-derived cholesterol esters (CEs) are delivered to lysosomes following LDL receptor (LDLR)-mediated endocytosis of LDL. After hydrolysis by lipid lipases, the resulting free cholesterol is exported from the lysosome and delivered to other organelles and membranes (including the PM) through the cooperative functions of the Niemann-Pick C1 and Niemann-Pick C2 (NPC1 and NPC2), whose dysfunction leads to severe neurological disorders^35^. In contrast, de novo cholesterol synthesis is regulated by sterol regulatory element-binding protein 2 (SREBP-2), which localizes to the ER^36–39^. When cellular cholesterol levels drop, SREBP-2 responds to the decreased levels of ER cholesterol and translocates to the Golgi. There, it undergoes sequential proteolytic cleavage to release its N-terminal transcriptional domain, which activates genes involved in cholesterol biosynthesis and uptake, including HMG-CoA reductase (HMGCR) and LDLR. This helps meet cellular demand and maintain cholesterol homeostasis.

To study the potential role of cholesterol in maintaining the functional integrity of lysosomes, we visualized cholesterol dynamics during mitophagy. Cholesterol accumulated on the surface of degradative lysosomes that contained damaged mitochondria. This cholesterol accumulation was mediated by PI4P-driven, OSBP-mediated cholesterol transport and coupled with increased production of cholesterol through SREBP-2 activation. Lipidomics analysis revealed a massive increase in the levels of free fatty acids (FAs) following mitophagy. These FAs, through their conversion to triacylglycerol (TAG), were stored in newly formed lipid droplets in a diacylglycerol acyltransferase 1 (DGAT1)-dependent manner. Mechanistically, cholesterol accumulation was required to maintain the degradative capacity and membrane integrity of lysosomes, ensuring the efficient digestion of damaged mitochondria and subsequent formation of lipid droplets. Together, these analyses reveal a critical role played by cholesterol in sustaining lysosomal function upon acute mitochondrial dysfunction, providing molecular insights into the links between mitochondrial damage, cholesterol homeostasis, and lysosomal integrity.

## Results

### Cholesterol accumulates in the membranes of degradative lysosomes following mitophagy

**Fig. 1:**
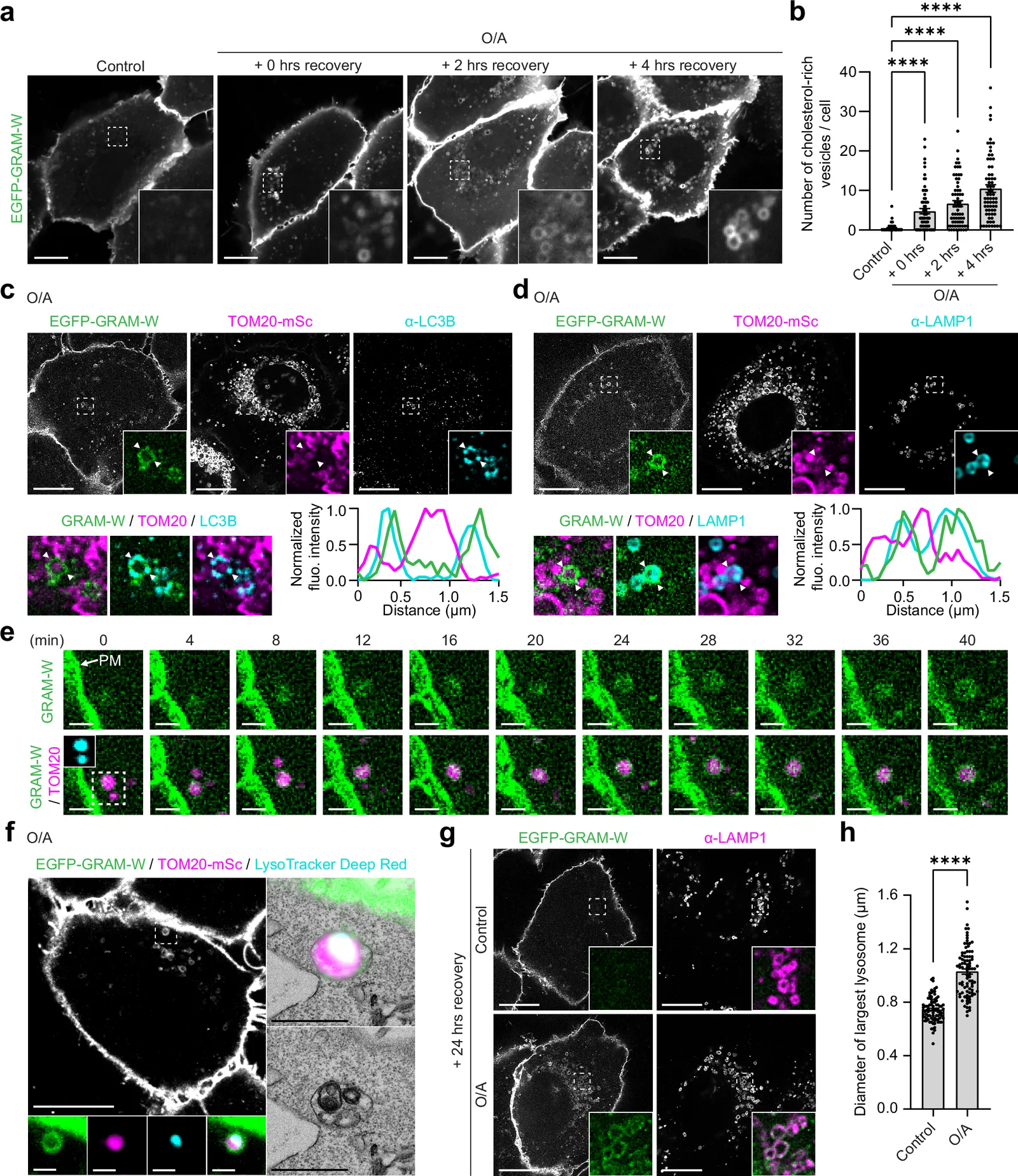
Cholesterol accumulates in the membranes of degradative lysosomes when mitochondria are depolarized. **a** Confocal images of live N/TERT cells expressing EGFP-GRAM-W that were treated with ethanol (Control) or 10 μM oligomycin and 4 μM antimycin A (O/A) for 2 hours, washed with phosphate-buffered saline (PBS) and incubated in keratinocyte serum-free medium (KSFM) for the indicated hours. Insets show at higher magnification the regions indicated by white dashed boxes. Scale bars, 10 µm. **b** Quantification of cholesterol-rich vesicles from (**a**) (mean ± SEM, *n* = 71, 72, 66, 69 cells; data are pooled from three independent experiments; one-way ANOVA with Dunnett’s multiple comparisons test, ****P < 0.0001). **c**, **d** SDC-SIM images of fixed N/TERT cells expressing EGFP-GRAM-W and TOM20-mScarlet (mSc) that were treated with O/A for 2 hours. Cells were immunolabeled with antibodies against LC3B (**c**) or LAMP1 (**d**). Scale bars, 10 µm. White arrowheads indicate a vesicle of interest. Graph: normalized line-scan quantifications of the fluorescence signals along the line as indicated by white arrowheads. **e** Timelapse SDC-SIM images of a degradative lysosome from live N/TERT cells expressing EGFP-GRAM-W and TOM20-mSc that were stained with LysoTracker Deep Red for 30 minutes and then treated with O/A for 30 minutes before imaging. Inset shows LysoTracker signals of the region indicated by a white dashed box. PM, plasma membrane. Scale bars, 1 µm. **f** Left: SDC-SIM images of fixed N/TERT cells expressing EGFP-GRAM-W and TOM20-mSc that were treated with O/A for 2 hours and stained with LysoTracker. Scale bar, 10 µm. Insets show at higher magnification the regions indicated by white dashed boxes. Scale bars, 1 µm. Right: Correlated SDC-SIM and transmission electron microscopy images of the same region. Scale bars, 1 µm. **g** SDC-SIM images of fixed N/TERT cells expressing EGFP-GRAM-W that were treated with ethanol (Control) or O/A for 2 hours, washed with PBS and recovered in KSFM for 24 hours. Cells were immunolabeled with antibodies against LAMP1. Scale bars, 10 µm. **h** Quantification of the diameter of the largest lysosome in cells from (**g**) (mean ± SEM, *n* = 86 cells for control and 88 for O/A; data are pooled from three independent experiments; Two-tailed unpaired t-test, ****P < 0.0001).

Human immortalized keratinocytes, N/TERT cells, rely primarily on de novo cholesterol synthesis rather than uptake, and at steady state, they exhibit low levels of cholesterol in lysosomal membranes^40^. This makes N/TERT cells a good model for monitoring changes in intracellular cholesterol distribution, particularly on vesicular structures like lysosomes. N/TERT cells stably expressing a cholesterol biosensor, EGFP-tagged GRAM-W (EGFP-GRAM-W)^40,41^, were co-treated with oligomycin and antimycin A (O/A), which are pharmacological inhibitors of mitochondria ATP synthase (Complex V) and cytochrome c reductase (Complex III), respectively. Their co-treatment induces mitochondrial depolarization and disrupts oxidative phosphorylation, thereby inducing mitophagy^42^. After 2 hours of treatment, drugs were removed, and cells were imaged at different time points during recovery using a spinning-disc confocal (SDC) microscope. Significant accumulation of cholesterol, as assessed by EGFP-GRAM-W, was observed on vesicular structures following two hours of O/A treatment. This accumulation increased during the recovery phase (Fig. 1a, b). Similar results were obtained when N/TERT cells were treated with other drugs known to induce mitochondrial depolarization, namely valinomycin, CCCP, FCCP, and TTFA (Fig. S1a). This indicates that mitochondrial depolarization is accompanied by a major alteration in the intracellular distribution of cholesterol. Staining fixed cells with filipin, a chemical probe for cholesterol, showed extensive overlap between EGFP-GRAM-W and filipin, confirming the accumulation of cholesterol on vesicular structures (Fig. S1b). A significant increase in cholesterol on lysosomal membranes was also detected using purified mCherry-tagged D4H proteins, another widely used probe for accessible cholesterol^43,44^ (Fig. S1c, d). Recent studies have reported the acute accumulation of cholesterol in ruptured lysosomes^45,46^. However, O/A treatment did not induce the rupture of lysosomal membranes, as assessed in fixed cells via immunostaining for galectin-3 (Gal-3) and for CHMP2A, a component of the ESCRT-III complex^47,48^ (Fig. S1e, f). In contrast, treatment with L-Leucyl-L-Leucine methyl ester (LLOMe), a compound known to disrupt lysosomal membranes, resulted in clear Gal-3 and CHMP2A recruitment (Fig. S1e, f). These results rule out membrane disruption as the cause of cholesterol accumulation during mitophagy.

Mitophagy requires engulfment of damaged mitochondria via autophagosomes, which subsequently fuse with lysosomes to degrade damaged mitochondria. While autophagosomes are positive for LC3B, their fusion with lysosomes results in degradative lysosomes (i.e., autolysosomes) that are additionally positive for the lysosomal marker LAMP1^12,49^. To examine the presence of cholesterol in these structures, cells stably expressing EGFP-GRAM-W together with a mitochondria marker, TOM20 tagged with mScarlet (TOM20-mSc), were treated with O/A for 2 hours and processed for immunostaining with antibodies against LC3B (Fig. 1c) or LAMP1 (Fig. 1d). Cells were then imaged under spinning-disc confocal structured illumination microscopy (SDC-SIM). The EGFP-GRAM-W signal overlapped with LC3B-positive vesicles that contained TOM20-mSc (Fig. 1c). In addition, EGFP-GRAM-W-positive structures were also positive for LAMP1, consistent with the accumulation of cholesterol on the membranes of degradative lysosomes (Fig. 1d). Quantification of 18 O/A-treated cells across three independent experiments identified a total of 1,831 lysosomes, among which 56% of lysosomes contained TOM20-mSc (Supplementary Table 1). We also determined the proportion of lysosomes decorated by EGFP-GRAM-W and assessed how many of these GRAM-W positive lysosomes contained TOM20-mSc. Among all the TOM20-positive lysosomes, 72% of those lysosomes were GRAM-W positive. These results provide additional evidence for the delivery of damaged mitochondria into lysosomes and further support that lysosomes containing damaged mitochondria are more frequently associated with elevated membrane cholesterol.

To monitor the time-course of cholesterol accumulation in degradative lysosomal membranes, individual vesicles that were positive for both TOM20-mSc and LysoTracker^TM^ Deep Red, a marker of the lysosome, were imaged every two minutes after addition of O/A (Fig. 1e and Supplementary Movie 1). EGFP-GRAM-W progressively accumulated in these structures over 40 minutes (Fig. 1e). This was consistent with the progressive accumulation of cholesterol on degradative lysosomes during mitophagy. Finally, correlative light and electron microscopy (CLEM) was performed to examine the ultrastructure of vesicles that were positive for both EGFP-GRAM-W and LysoTracker^TM^ Deep Red and that contained TOM20-mSc (Fig. 1f). These vesicles contained electron dense elements, further supporting their identity as degradative lysosomes.

To investigate whether the accumulation of cholesterol on degradative lysosomes is transient or sustained, cells were treated with O/A for 2 hours, allowed to recover for 24 hours, and then immunostained for LAMP1. Degradative lysosomes became larger in O/A-treated condition, as revealed by LAMP1 staining (Fig. 1g, h), indicating that mitophagy continued during the recovery period with ongoing lysosome-autophagosome fusion. Importantly, cholesterol remained highly enriched on these degradative lysosomal membranes, suggesting sustained accumulation throughout the mitophagy process.

O/A treatment induced cholesterol accumulation, as assessed by EGFP-GRAM-W, in other cell lines as well, including HeLa, NIH3T3, and U2OS cells (Fig. S1g, h). However, less cholesterol accumulated than seen in O/A-treated N/TERT cells, likely due to the relatively high basal EGFP-GRAM-W signals on lysosomes of these cell lines at steady state (Fig. S1g).

To determine whether the observed cholesterol accumulation is specific to depolarization-induced mitophagy, deferiprone (DFP) was used to induce a mechanistically distinct form of mitophagy via iron depletion^50^. N/TERT cells treated with DFP for 24 hours showed major accumulation of cholesterol on enlarged lysosomes that were labeled by LAMP1-miRFP703 (Fig. S1i), suggesting that mitophagy triggers cholesterol accumulation on degradative lysosomal membranes irrespective of the upstream mechanism.

Finally, we investigated whether lysosomal cholesterol accumulation is a general feature of autophagy. To this end, N/TERT cells were starved using Hanks’ balanced salt solution (HBSS) to trigger nutrient deprivation-dependent autophagy. Cells treated with HBSS for 24 hours showed some accumulation of cholesterol on vesicular structures. However, they were not labeled with LAMP1-miRFP703, suggesting that the cholesterol accumulation in this condition primarily occurred on non-lysosomal compartments (Fig. S1j).

Taken together, our results show that mitophagy leads to the dramatic accumulation of cholesterol on degradative lysosomal membranes, suggesting that cholesterol plays a critical role in this process.

### Cholesterol accumulates on degradative lysosomes through PI4P-driven, OSBP-mediated cholesterol transport

**Fig. 2:**
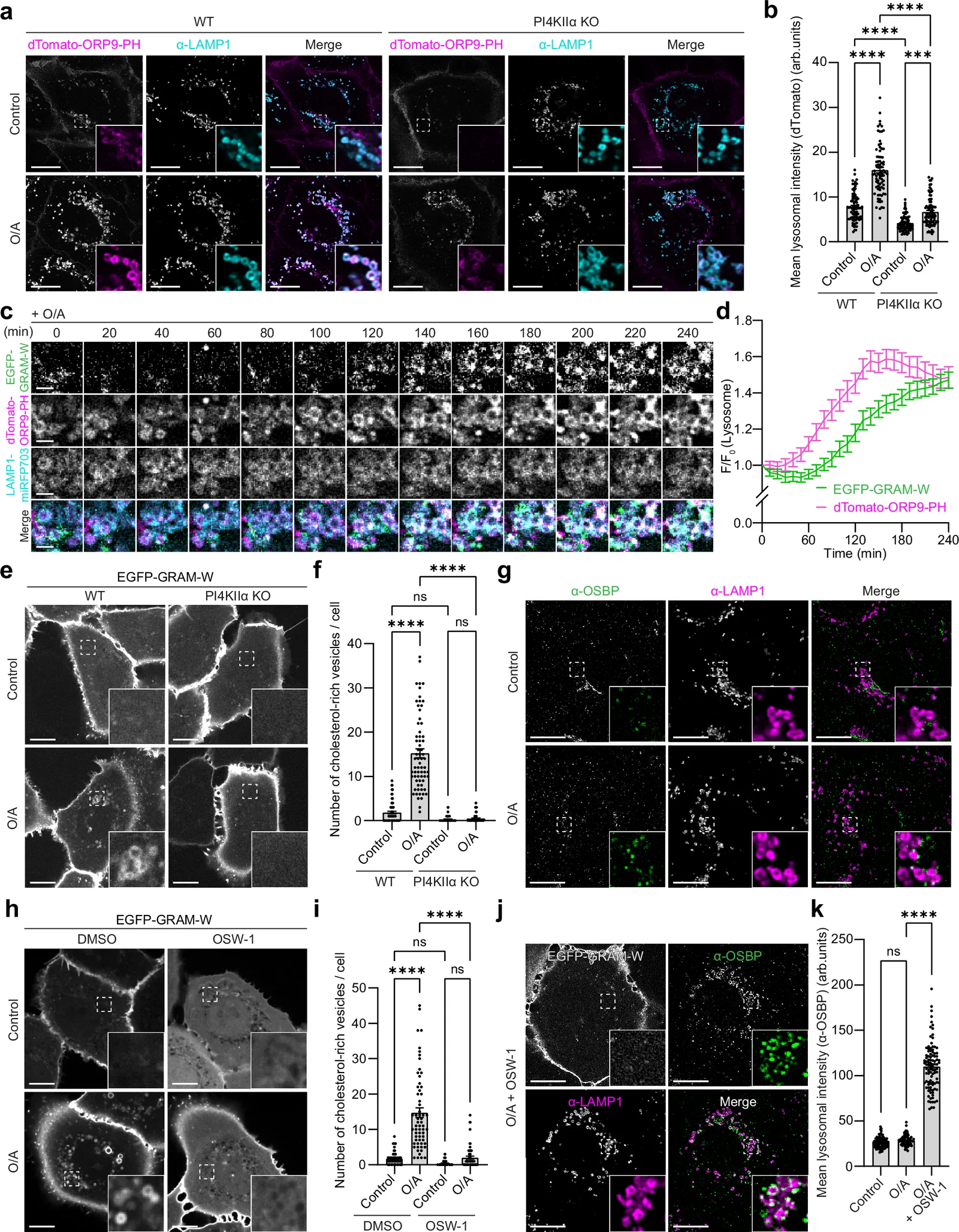
Cholesterol accumulates on degradative lysosomes through PI4P-driven, OSBP-mediated cholesterol transport. **a** SDC-SIM images of fixed wild-type (WT) and PI4KIIα knockout (KO) N/TERT cells expressing dTomato-ORP9-PH that were treated with ethanol (Control) or O/A for 2 hours and immunolabeled against LAMP1. Scale bars, 10 µm. arb., arbitrary. **b** Quantification of dTomato-ORP9-PH signals on lysosomal membranes from (**a**) (mean ± SEM, *n* = 80, 81, 79, 83 cells; data are pooled from three independent experiments; one-way ANOVA with Tukey’s multiple comparisons test, ***P = 0.0001, ****P < 0.0001). **c** SDC-SIM images of live N/TERT cells expressing indicated constructs that were treated with O/A for the indicated time. Scale bars, 1 µm. **d** Time course of lysosomal signals from (**c**) (mean ± SEM, *n* = 19 cells; data are pooled from four independent experiments). **e** Confocal images of live WT or PI4KIIα KO N/TERT cells expressing EGFP-GRAM-W that were treated with ethanol (Control) or O/A for 2 hours. Scale bars, 10 µm. **f** Quantification of cholesterol-rich vesicles from (**e**) (mean ± SEM, *n* = 73, 69, 61, 64 cells; data are pooled from three independent experiments; one-way ANOVA with Tukey’s multiple comparisons test, ****P < 0.0001, ns denotes not significant). **g** SDC-SIM images of fixed N/TERT cells expressing EGFP-GRAM-W that were treated with ethanol (Control) or O/A for 2 hours and immunolabeled as indicated. Scale bars, 10 µm. **h** Confocal images of live N/TERT cells expressing EGFP-GRAM-W, that were pre-treated with DMSO or 20 nM OSW-1 for 4 hours and then treated with ethanol (Control) or O/A for 2 hours in the presence of the same drug. Scale bars, 10 µm. **i** Quantification of cholesterol-rich vesicles from (**h**) (mean ± SEM, *n* = 62, 62, 66, 62 cells; data are pooled from three independent experiments; one-way ANOVA with Tukey’s multiple comparisons test, ****P < 0.0001). **j** SDC-SIM images of fixed N/TERT cells expressing EGFP-GRAM-W, that were pre-treated with O/A for 1.5 hours and then treated with 20 nM OSW-1 for 0.5 hours in the presence of O/A. Cells were immunolabeled as indicated. Scale bars, 10 µm. **k** Quantification of α-OSBP signals on lysosomal membranes from (**g**) and (**j**) (mean ± SEM, *n* = 99 cells for each condition; data are pooled from three independent experiments; one-way ANOVA with Tukey’s multiple comparisons test, ****P < 0.0001).

Recent studies have highlighted the role of PI4P in promoting autophagy during nutrient starvation^18^. To assess the potential involvement of PI4P during O/A-induced accumulation of cholesterol on degradative lysosomal membranes, we generated N/TERT cells that stably expressed a PI4P biosensor, namely the pleckstrin homology (PH) domain of OSBP-related protein 9 (ORP9) tagged with dTomato (dTomato-ORP9-PH)^51^. In cells treated with O/A for 2 hours, the levels of PI4P, as assessed by dTomato-ORP9-PH, were significantly elevated on degradative lysosomal membranes that were positive for both LAMP1 and EGFP-GRAM-W (Fig. 2a, b, Fig. S2a). A significant increase in PI4P on lysosomal membranes was also detected using EGFP-tagged PH domain of FAPP1, another biosensor for PI4P^52,53^ (Fig. S2b, c). Importantly, time-lapse imaging showed that the PI4P accumulation on lysosomal membranes preceded cholesterol accumulation, as assessed by EGFP-GRAM-W, indicating that PI4P may drive the accumulation of cholesterol on degradative lysosomal membranes during mitophagy (Fig. 2c, d). PI4KIIα, which generates PI4P from phosphatidylinositol, plays a major role in the accumulation of PI4P on degradative lysosomal membranes upon nutrient starvation-induced autophagy^18^. To ask whether PI4KIIα plays a similar role in the context of mitophagy, we generated PI4KIIα knockout (KO) N/TERT cells via CRISPR/Cas9-mediated gene editing (Fig. S2d) and treated them with O/A. Compared with control, less PI4P accumulated on degradative lysosomal membranes of PI4KIIα KO cells (Fig. 2a, b). Strikingly, O/A-induced accumulation of cholesterol on degradative lysosomal membranes was completely abolished in PI4KIIα KO cells (Fig. 2e, f). This indicates that in response to mitophagy, PI4KIIα drives the accumulation of PI4P on degradative lysosomal membranes, which in turn recruits cholesterol to these same membranes.

PI4P serves as a major driving force for cholesterol transport by regulating OSBP activity, which facilitates the counter-transport of PI4P and cholesterol at various membrane contact sites formed between the ER and other organelles^54^. To investigate the potential involvement of OSBP in cholesterol accumulation on degradative lysosomal membranes, N/TERT cells were treated with O/A for 2 hours and processed for immunostaining with antibodies against OSBP and LAMP1 (Fig. 2g). The localization of OSBP is highly dynamic, as it dissociates from cellular membranes when mediating the counter-transport of PI4P and cholesterol^28^. Compared to control cells, in which OSBP primarily localized to the trans-Golgi network (TGN) (Figs. 2g, S2e), OSBP was re-distributed throughout the cytoplasm in cells treated with O/A (Fig. 2g). OSBP can be trapped at sites where it is active by treatment with OSW-1, a chemical inhibitor of OSBP that blocks its counter-transport function^29^. Co-treatment with OSW-1, but not with dimethyl sulfoxide (DMSO), suppressed O/A-induced lysosomal cholesterol accumulation (Fig. 2h, i). Remarkably, OSBP was trapped on degradative lysosomal membranes (Fig. 2j, k), supporting the hypothesis that OSBP plays a major role in driving cholesterol transport to degradative lysosomes during mitophagy. Importantly, OSBP was significantly less trapped on degradative lysosomal membranes in PI4KIIα KO cells compared to wild-type cells (Fig. S2f, g). These results suggest that PI4P generated by PI4KIIα is the major trigger to recruit and activate OSBP at ER-lysosome contact sites.

Finally, we generated N/TERT cells stably expressing an ER marker, BFP-tagged Sec61β, and imaged ER morphology following exposure to O/A. In O/A-treated cells, the ER network was disrupted, lacking the clear sheets typically observed in untreated control cells (Fig. S2h). Notably, the ER and EGFP-GRAM-W-positive vesicles were in close association in O/A-treated cells (Fig. S2h), supporting the presence of close contact between the ER and lysosomes under this condition.

Together, these results demonstrate that mitophagy is coupled with PI4KIIα-dependent production of PI4P on degradative lysosomal membranes, which in turn promotes OSBP-mediated cholesterol delivery at ER-lysosome contact sites, leading to cholesterol accumulation on these membranes.

### Cholesterol accumulation on degradative lysosomal membranes is accompanied by SREBP-2 activation and enhanced cholesterol biosynthesis

**Fig. 3:**
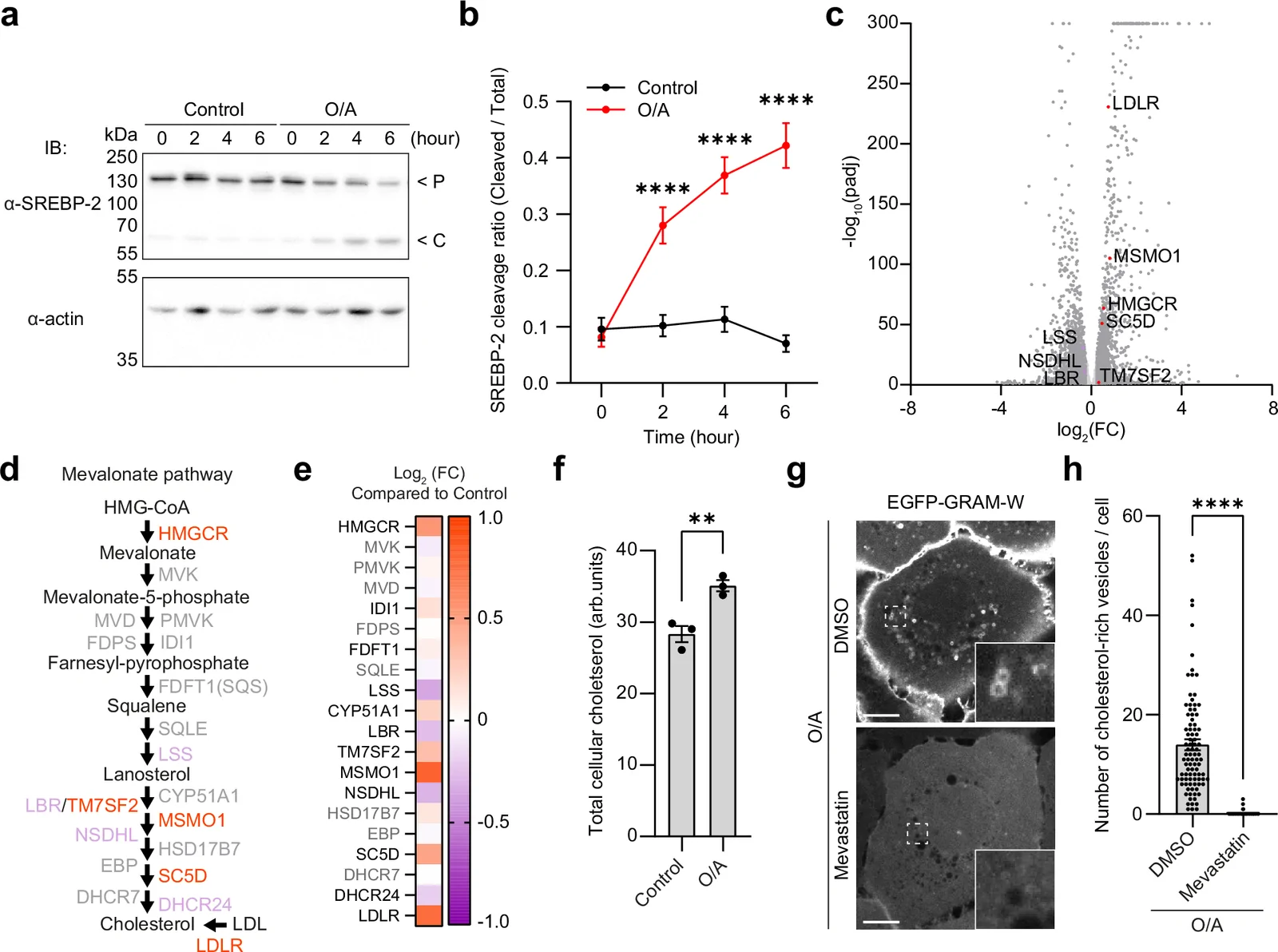
Cholesterol accumulation on degradative lysosomal membranes is accompanied by SREBP-2 activation and enhanced cholesterol biosynthesis. **a** Lysates of N/TERT cells treated with ethanol (Control) or O/A for the indicated time were processed for SDS-PAGE and immunoblotting (IB). Precursor (P) and cleaved (C) forms of SREBP-2 are indicated. **b** Quantification of the ratio of cleaved to total SREBP-2 from (**a**) (mean ± SEM, *n* = 8 lysates from independent experiments for each condition; two-way ANOVA with Šídák’s multiple comparisons test, ****P < 0.0001 compared to Control at the same timepoint). **c** Volcano plot showing transcriptome-wide changes in gene expression in N/TERT cells treated with O/A for 4 hours compared to ethanol. Data are presented with fold change in log_2_ [log_2_(FC)] and adjusted *P* values in -log_10_ [-log_10_(padj)] (DESeq2, two-sided Wald test, Benjamini-Hochberg-adjusted *P* values). Data points with -log_10_(padj) over 300 are presented as 300. Dark gray dots are differentially expressed genes [|log_2_(FC)| > 0.25]. Red dots are representative genes involved in the mevalonate pathway of cholesterol biosynthesis. Data are pooled from four independent experiments. **d** The mevalonate pathway synthesizes cholesterol, whereas LDLR mediates the uptake of cholesterol in the form of LDL. Red/purple represent upregulated/downregulated genes in O/A-treated N/TERT cells compared to ethanol-treated N/TERT cells [|log_2_(FC)| > 0.25, adjusted *P* values < 0.05]. **e** Heatmap displaying log_2_(FC) of genes from (**d**). Gray indicates genes with adjusted *P* value > 0.05. **f** Quantification of total cellular free cholesterol in N/TERT cells treated with ethanol (Control) or O/A for 2 hours, washed with PBS and recovered in KSFM for 24 hours. Lipids were extracted from the cells, and the amount of total cellular free cholesterol was assessed using Cholesterol Glo^TM^ Assay. Total cellular free cholesterol was normalized with cell number (mean ± SEM, *n* = 3 independent experiments for each condition; Two-tailed unpaired t-test, **P = 0.0079). **g** Confocal images of live N/TERT cells expressing EGFP-GRAM-W, that were pre-treated with DMSO or 5 µM mevastatin for 18 hours and then treated with O/A for 2 hours in the presence of the same drug. Scale bars, 10 µm. **h** Quantification of cholesterol-rich vesicles from (**g**) (mean ± SEM, *n* = 92 cells for each condition; data are pooled from four independent experiments; Two-tailed unpaired t-test, ****P < 0.0001).

Our previous study showed that hyperactivation of OSBP, which helps reduce levels of cholesterol in the ER, is coupled with the activation of SREBP-2^30^. To ask if levels of SREBP-2 activity change during mitophagy, we assessed levels of its cleaved, transcriptionally active form. Cells were treated with ethanol (control) or O/A for 2, 4, or 6 hours and lysates were analyzed via Western blotting using antibodies against SREBP-2. The ratio of cleaved SREBP-2 ( ~ 60 kDa) to total SREBP-2 (cleaved + the ~135 kDa precursor) was compared. In control cells, ~10% of SREBP-2 was cleaved (Fig. 3a). In contrast, more than 30% of SREBP-2 was cleaved in cells treated with O/A for 2 hours, with 40% cleaved at 6 hours of treatment, revealing acute activation of SREBP-2 during mitophagy (Fig. 3b).

The cleaved/activated form of SREBP-2 enters the nucleus and increases the expression of genes that enhance cholesterol production and uptake^33,36,37^. Thus, mRNA sequencing analysis was performed to examine whether O/A-induced SREBP-2 cleavage contributes to the upregulation of genes involved in cholesterol biosynthesis and uptake. We treated cells with ethanol (control) or O/A, extracted total RNA, constructed mRNA libraries, and performed massively parallel sequencing. Remarkably, a number of genes involved in cholesterol biosynthesis and uptake, including LDLR and HMGCR (the ER-localized rate-limiting enzyme of the mevalonate pathway), were upregulated in cells treated with O/A (Fig. 3c–e, Fig. S3).

The activation of SREBP-2 and transcription of its target genes are likely coupled with increased production of cholesterol in O/A-treated cells. To measure total cellular cholesterol levels, lipids were extracted from N/TERT cells treated with ethanol (control) or O/A, and total cellular cholesterol was measured using the Cholesterol Glo^TM^-assay kit. O/A-treated cells contained ~24% more cholesterol compared to controls (Fig. 3f), showing that SREBP-2 activation during mitophagy results in increased cholesterol production.

Finally, we investigated the contribution of de novo cholesterol synthesis to the accumulation of cholesterol on degradative lysosomal membranes. Treatment of cells with mevastatin, which inhibits HMGCR, resulted in cholesterol depletion, as revealed by the dissociation of EGFP-GRAM-W from the PM (Fig. 3g). Importantly, mevastatin also prevented O/A-induced cholesterol accumulation on degradative lysosomes (Fig. 3g, h).

Collectively, these results demonstrate that mitophagy promotes cholesterol biosynthesis via the activation of SREBP-2. These analyses also confirm that the cholesterol that accumulates on degradative lysosomal membranes is synthesized de novo in the ER.

### Activation of SREBP-2 is driven by OSBP-mediated cholesterol transport from the ER to the lysosome

**Fig. 4:**
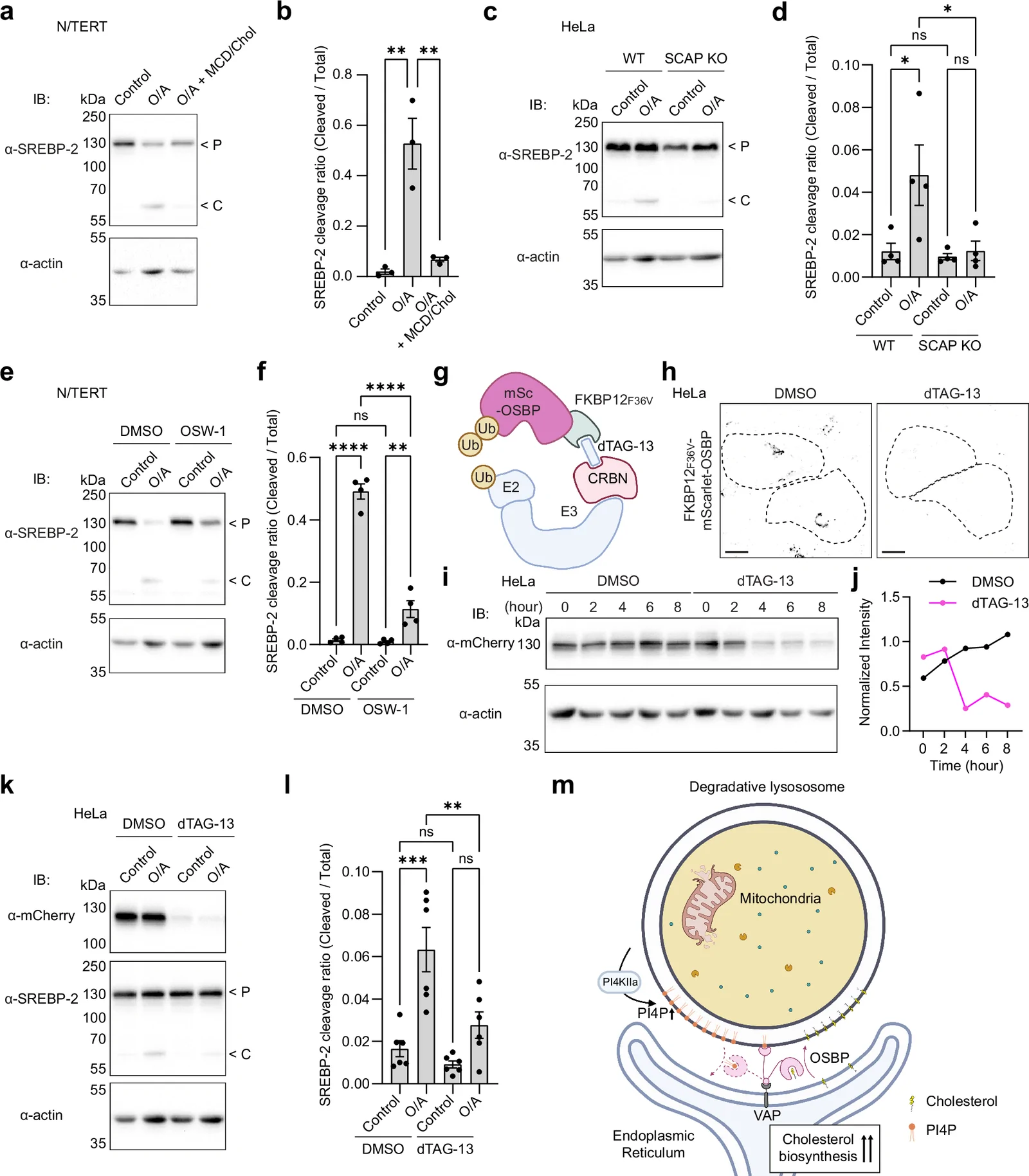
Activation of SREBP-2 is driven by OSBP-mediated cholesterol transport from the ER to the lysosome. **a**, **c** Lysates of N/TERT cells treated with ethanol (Control), O/A, or O/A plus 200 μM MCD-cholesterol complexes (O/A + MCD/Chol) for 4 hours (**a**) and wild-type (WT) and SCAP knockout (KO) HeLa cells treated with ethanol (Control) or O/A for 4 hours (**c**) were processed for SDS-PAGE and immunoblotting (IB). Precursor (P) and cleaved (C) forms of SREBP-2 are indicated. **b**, **d** Quantification of the ratio of cleaved to total SREBP-2 from (**a**) and (**c**) (mean ± SEM, *n* = 3 (**b**) or 4 (**d**) lysates from three (**b**) or four (**d**) independent experiments; one-way ANOVA with Tukey’s multiple comparisons test, **P = 0.0021 for Control and O/A, *P* = 0.0035 for O/A and O/A + MCD/Chol, *P = 0.0297 for WT Control and WT O/A, *P* = 0.0309 for WT O/A and SCAP KO O/A, ns denotes not significant). **e** Lysates of N/TERT cells treated with ethanol (Control) or O/A for 4 hours in the presence of DMSO or 20 nM OSW-1 were processed for SDS-PAGE and IB. **f** Quantification of the ratio of cleaved to total SREBP-2 from (**e**) (mean ± SEM, *n* = 4 lysates from four independent experiments; one-way ANOVA with Tukey’s multiple comparisons test, **P = 0.0083, ****P < 0.0001). **g** dTAG-13-induced degradation system for acute depletion of endogenous OSBP. Created in BioRender. Saheki, Y. (2026) https://BioRender.com/qhsicas. **h** Confocal images of live FKBP12_F36V_-mScarlet-OSBP knock-in (KI) HeLa cells that were treated with DMSO or 10 μM dTAG-13 for 24 hours. Dotted lines, cell boundaries. Scale bars, 10 µm. **i** Lysates of FKBP12_F36V_-mScarlet-OSBP KI HeLa cells treated as (**h**) for the indicated times were processed for SDS-PAGE and IB as indicated. **j** Quantification of FKBP12_F36V_-mScarlet-OSBP from (**i**). **k** Lysates of FKBP12_F36V_-mScarlet-OSBP KI HeLa cells pre-treated as indicated for 6 hours, followed by treatment with ethanol (Control) or O/A for 4 hours in the same drug, were processed for SDS-PAGE and IB. **l** Quantification of the ratio of cleaved to total SREBP-2 from (**k**) (mean ± SEM, *n* = 6 lysates from six independent experiments; one-way ANOVA with Tukey’s multiple comparisons test, **P = 0.0044, ***P = 0.0003). **m** SREBP-2 activation is driven by OSBP-mediated ER-to-lysosome cholesterol transport. Created in BioRender. Saheki, Y. (2026) https://BioRender.com/zkm3vkl.

Since a decrease in cholesterol levels in the ER can activate SREBP-2, we asked whether cholesterol supplementation could suppress O/A-induced SREBP-2 activation. N/TERT cells were treated with ethanol, O/A, or O/A plus methyl-β-cyclodextrin (MCD)-loaded cholesterol (MCD/Chol) for 4 hours, and then lysates were analyzed by Western blotting using antibodies against SREBP-2. Compared to O/A-treated cells, cells co-treated with O/A and MCD/Chol exhibited less SREBP-2 cleavage (Fig. 4a, b), suggesting that mitophagy-induced SREBP-2 cleavage is associated with depletion of cholesterol from the ER.

Following ER cholesterol depletion, ER-localized SREBP-2 translocates to the Golgi, where it is cleaved. This process requires SCAP, which forms a complex with SREBP-2 and regulates its cholesterol-dependent shuttling between the ER and the Golgi^37^. To examine the requirement of SCAP in O/A-induced SREBP-2 cleavage, SCAP KO cells were generated via CRISPR/Cas9-mediated gene editing. SCAP KO was confirmed by the absence of SREBP-2 cleavage upon cholesterol depletion (Fig. S4a). Whereas HeLa cells tolerated SCAP KO, this genetic deletion was lethal in N/TERT cells, likely due to its essential role in keratinocyte viability. Therefore, WT and SCAP KO HeLa cells were treated with ethanol (control) or O/A, and cell lysates were subjected to Western blotting using antibodies against SREBP-2. Importantly, O/A treatment induced SREBP-2 cleavage in HeLa cells (as seen in N/TERT cells), albeit to a lesser extent (Fig. 4c, d). Strikingly, O/A-induced SREBP-2 cleavage was abolished in SCAP KO HeLa cells (Fig. 4c, d). These results are consistent with the requirement of SCAP-mediated trafficking of SREBP-2 from the ER to the Golgi, a process that depends on ER cholesterol depletion.

Given OSBP’s role in promoting cholesterol efflux from the ER, its activation during mitophagy may contribute to SREBP-2 cleavage. Therefore, we examined the effect of OSBP inhibition on O/A-induced SREBP-2 cleavage. We first co-treated N/TERT cells with O/A and either the OSBP inhibitor OSW-1 or DMSO. The extent of SREBP-2 cleavage was analyzed via Western blotting. Compared to DMSO-treated cells, co-treatment with OSW-1 suppressed O/A-induced SREBP-2 cleavage (Fig. 4e, f). As a second test, CRISPR/Cas9-mediated gene-editing was used to generate HeLa cells in which the endogenous OSBP was tagged with FKBP12_F36V_ and mScarlet-I (Fig. S4b). When these cells are treated with dTAG-13, FKBP12_F36V_-tagged OSBP is recruited to cereblon (CRBN), an E3 ligase adaptor protein. It is then ubiquitinated and degraded by the proteasome^55^ (Fig. 4g). HeLa cells expressing FKBP12_F36V_-mScarlet-I-OSBP were treated with DMSO or dTAG-13 for 24 hours, and the extent of OSBP degradation was assessed via SDC microscopy and Western blotting using antibodies against OSBP and mCherry, which also detects mScarlet-I. Cells treated with dTAG-13 exhibited near complete loss of the mScarlet-I signal, indicating efficient degradation of endogenous OSBP by the degron system (Fig. 4h and Fig. S4b). After confirming the effectiveness of this approach, cells were then treated with DMSO or dTAG-13 for 2, 4, 6, or 8 hours and the extent of degradation was analyzed via Western blotting. FKBP12_F36V_-mScarlet-I-OSBP was significantly depleted after 4 hours of dTAG-13 treatment (Fig. 4i, j). Taking advantage of this strategy, we investigated the impact of acute OSBP degradation in O/A-induced SREBP-2 cleavage. Cells were pretreated with DMSO or dTAG-13 for 6 hours and further treated with O/A for 4 hours. Lysates from these cells were subjected to Western blotting using antibodies against mCherry (to confirm OSBP degradation) and SREBP-2 (to evaluate SREBP-2 cleavage). Importantly, OSBP degradation suppressed O/A-induced SREBP-2 cleavage (Fig. 4k, l), confirming that OSBP activity is responsible for SREBP-2 activation during mitophagy.

A previous study has shown that OSBP-mediated cholesterol transport from the ER to lysosomes can also be elicited by LLOMe-induced lysosomal membrane rupture^45^. Hence, we investigated whether LLOMe treatment also induced SREBP-2 cleavage. Cells were treated with LLOMe for 1 hour to induce lysosomal membrane rupture and cell lysates were analyzed by Western blotting using antibodies against SREBP-2. Remarkably, SREBP-2 was efficiently cleaved following LLOMe treatment, indicating acute activation of SREBP-2 upon lysosomal membrane rupture (Fig. S4c, d). Importantly, this cleavage was inhibited by OSW-1, highlighting the conserved role of OSBP in SREBP-2 activation during mitophagy and in the context of lysosomal membrane rupture (Fig. S4e, f).

Taken together, these results demonstrate that mitophagy triggers PI4P-driven OSBP-mediated cholesterol transport, leading to ER cholesterol depletion and subsequent activation of SREBP-2. This in turn drives increased cholesterol production to maintain high levels of cholesterol on degradative lysosomal membranes (Fig. 4m).

### Cholesterol contributes to the degradative capacity and membrane integrity of degradative lysosomes

**Fig. 5:**
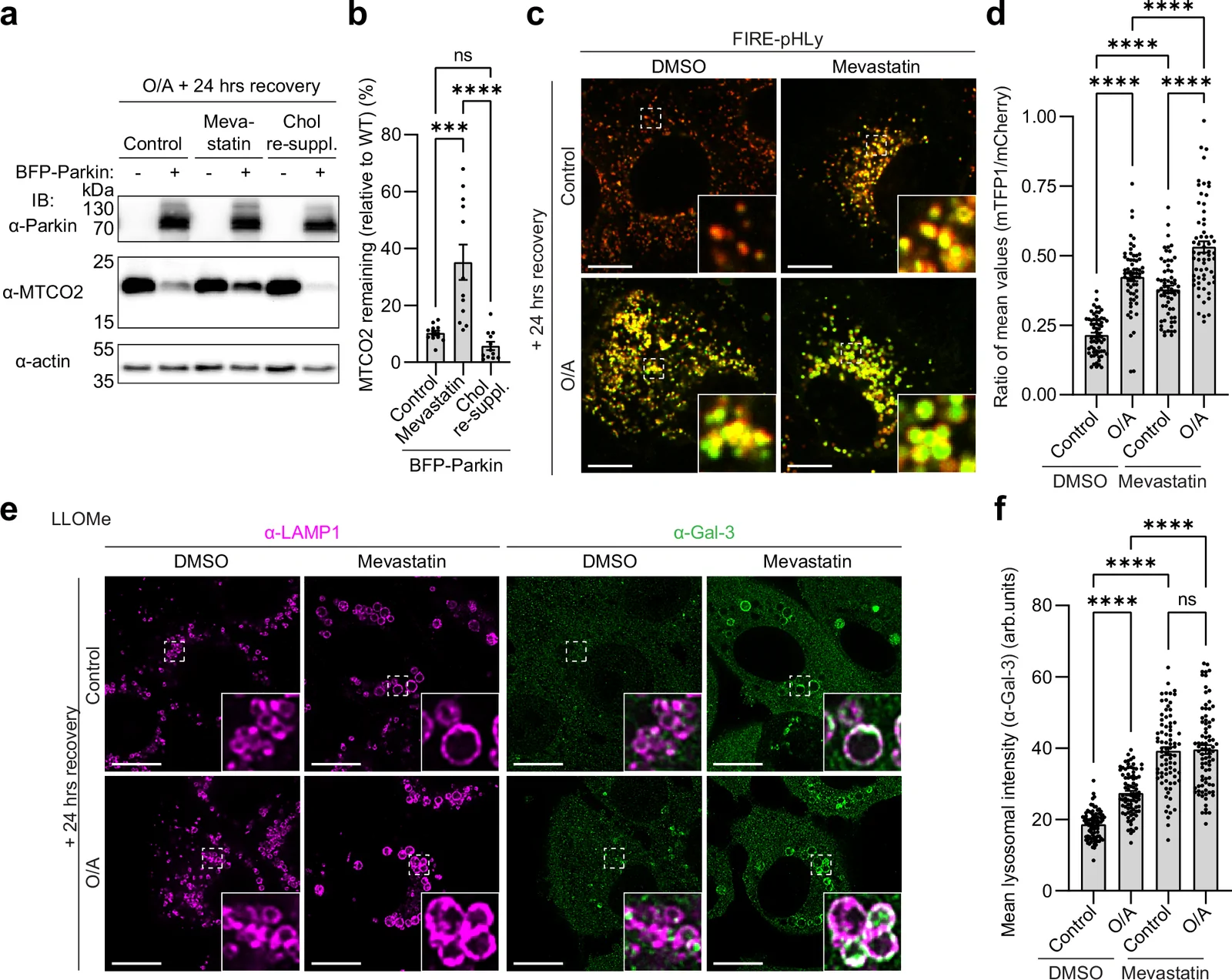
Cholesterol contributes to the degradative capacity and membrane integrity of degradative lysosomes. **a** Lysates of N/TERT wild-type (WT) and BFP-Parkin expressing cells were processed for SDS-PAGE and immunoblotting (IB) as indicated. Cells pre-treated with DMSO (Control) or 25 µM mevastatin for 24 hours were treated with O/A for 2 hours in the presence of DMSO (Control), 25 µM mevastatin (Mevastatin), or 2.5 µM MCD/cholesterol (Chol re-suppl.). Cells were then washed with PBS and recovered in KSFM containing the same drugs for 24 hours. **b** Quantification of MTCO2 levels in BFP-Parkin-expressing cells, normalized to those in WT cells, from (**a**) (mean ± SEM, *n* = 12 lysates from twelve independent experiments for each condition; one-way ANOVA with Tukey’s multiple comparisons test, ***P = 0.0001, ****P < 0.0001, ns denotes not significant). **c** Confocal images of live N/TERT cells expressing FIRE-pHLy that were pre-treated with DMSO or 5 µM mevastatin for 16 hours and then treated with ethanol (Control) or O/A for 2 hours in the presence of the same drug. Cells were then washed with PBS and recovered in KSFM containing the same drugs for 24 hours. Insets show at higher magnification the regions indicated by white dashed boxes. Scale bars, 10 µm. **d** Quantification of the ratio of mean fluorescent intensity of mTFP1 to that of mCherry from (**c**) (mean ± SEM, *n* = 63 cells for each condition; data are pooled from three independent experiments; one-way ANOVA with Tukey’s multiple comparisons test, ****P < 0.0001). **e** SDC-SIM images of fixed N/TERT cells that were pre-treated with DMSO or 5 µM mevastatin for 16 hours and then treated with ethanol (Control) or O/A for 2 hours. Cells were then washed with PBS and recovered in KSFM containing the same drugs for 24 hours. Cells were finally treated with 1 mM of LLOMe for 5 minutes and immunolabeled against LAMP1 and galectin-3 (Gal-3). Scale bars, 10 µm. **f** Quantification of α-Gal-3 signals on lysosomal membranes from (**e**) (mean ± SEM, *n* = 83, 83, 80, 83 cells; data are pooled from three independent experiments; one-way ANOVA with Tukey’s multiple comparisons test, ****P < 0.0001).

The robust accumulation of cholesterol on degradative lysosomal membranes suggests that this lipid may play an important role in lysosomal function and integrity. We first asked whether efficient degradation of depolarized mitochondria requires cholesterol. To address this, we generated N/TERT cells stably expressing BFP-tagged Parkin and examined mitochondrial depolarization-dependent degradation of MTCO2 (COXII) by Western blotting of lysates from cells treated with O/A for 2 hours, followed by a 24-hour recovery, in the absence or presence of mevastatin to deplete cholesterol. In wild-type control cells, MTCO2 remained largely detectable, whereas BFP-Parkin-expressing cells showed robust MTCO2 degradation, consistent with previous reports^42,56^ (Fig. 5a). Notably, MTCO2 clearance was significantly reduced in cells treated with mevastatin (Fig. 5a, b). Furthermore, MTCO2 clearance was restored by cholesterol re-supplementation through the removal of mevastatin and the addition of MCD/Chol, following 24 hours of mevastatin treatment (Fig. 5a, b). These results demonstrate that cholesterol is required for efficient degradation of damaged mitochondria.

The degradative capacity of lysosomes depends on their low pH, which provides the acidic environment necessary for efficient breakdown of various substrates. A recent study reported a potential role of cholesterol in the assembly of the vacuolar ATPase (V-ATPase) at the TGN to maintain acidity of the TGN lumen^57^. Since a structurally similar V-ATPase functions at lysosomes^58,59^, we asked whether cholesterol contributes to the maintenance of lysosomal acidity. To monitor lysosomal pH, we used a genetically encoded, pH-sensitive biosensor, FIRE-pHLy (mTFP1-LAMP1-mCherry)^60^. While cytosolic mCherry maintains constant fluorescence, the fluorescence of luminal mTFP1 varies with pH (low when exposed to the acidic pH of the lysosomal lumen, with fluorescence increasing as the luminal pH rises) (Fig. S5a). To examine the impact of mitophagy and cholesterol depletion on lysosomal pH, N/TERT cells stably expressing FIRE-pHLy were generated and treated with ethanol (control) or O/A for 2 hours, allowed to recover for 24 hours, in the absence or presence of mevastatin to induce cholesterol depletion. O/A-induced mitophagy resulted in the deacidification of lysosomes. This was enhanced by cholesterol depletion. Importantly, cholesterol depletion alone induced the deacidification of lysosomes, highlighting the importance of cholesterol in maintaining lysosomal pH and degradative capacity (Fig. 5c, d).

Finally, we examined the impact of cholesterol depletion on lysosomal membrane integrity by immunostaining fixed cells with antibodies against Gal-3. No rupture was observed in N/TERT cells treated with O/A, regardless of cholesterol depletion (Fig. S5b). Thus, LLOMe was used to assess the resilience of lysosomal membranes against acute damages. N/TERT cells were treated with ethanol (control) or O/A in the absence or presence of cholesterol depletion (via mevastatin) and additionally treated with LLOMe for a short duration (5 minutes). The extent of lysosomal membrane rupture was then compared. Whereas LLOMe treatment alone did not result in significant lysosomal membrane rupture, additional treatment with O/A induced substantial lysosomal membrane rupture, suggesting that lysosomal membranes become more fragile during mitophagy (Fig. 5e, f). Notably, mevastatin treatment caused greater lysosomal membrane rupture, with no further increase observed upon combined treatment with O/A (Fig. 5e, f). These results demonstrate that cholesterol plays a protective role in maintaining lysosomal membrane integrity. Because LLOMe requires an acidic environment for enzymatic activation, elevated lysosomal pH, as shown earlier in O/A or mevastatin-treated cells, could reduce the potency of LLOMe to induce membrane damage. However, despite this potentially inhibitory pH shift, N/TERT cells treated with O/A or mevastatin showed a dramatic enhancement in lysosomal membrane rupture upon LLOMe exposure (Fig. 5e, f). The size of degradative lysosomes increased notably upon cholesterol depletion, particularly when lysosomal damage was induced by LLOMe (Fig. 5e). These findings suggest that cholesterol is critical for preserving lysosomal membrane resilience in response to damage, and that cholesterol supports both the degradative capacity and membrane integrity of degradative lysosomes.

### Depolarization of mitochondria is followed by the formation of DGAT1-dependent lipid droplets

**Fig. 6:**
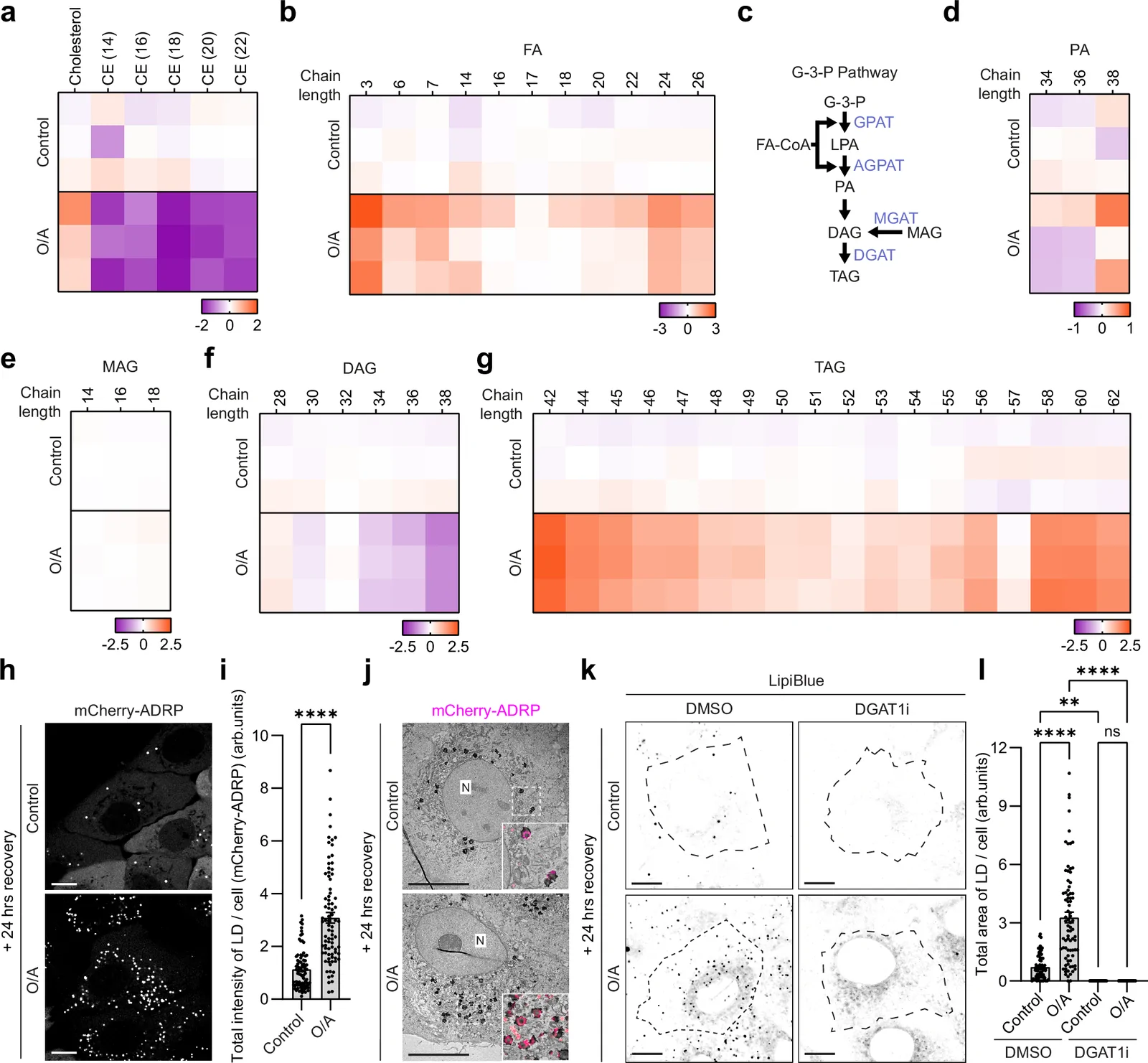
Depolarization of mitochondria is followed by the formation of DGAT1-dependent lipid droplets. **a**, **b** Heatmaps displaying the normalized amount of free cholesterol (**a**) cholesterol ester (CE) (**a**) and fatty acid (FA) (**b**) in N/TERT cells treated with ethanol (Control) or O/A for 2 hours, washed with PBS, and recovered in KSFM for 24 hours before lipid extraction and analysis by LC-MS/MS. **c** The G-3-P pathway for triacylglycerol (TAG) biosynthesis. **d**–**g** Heatmaps displaying the normalized amount of phosphatidic acid (PA) (**d**), monoacylglycerol (MAG) (**e**), diacylglycerol (DAG) (**f**), and TAG (**g**) in N/TERT cells treated with ethanol (Control) or O/A for 2 hours, washed with PBS, and recovered in KSFM for 24 hours before lipid extraction and analysis by LC-MS/MS. **h** Confocal images of live N/TERT cells expressing mCherry-ADRP that were treated with ethanol (Control) or O/A for 2 hours, washed with PBS, and recovered in KSFM for 24 hours. Scale bars, 10 µm. **i** Quantification of lipid droplets (LDs) from (**h**) (mean ± SEM, *n* = 82 cells for control and 83 cells for O/A; data are pooled from three independent experiments; Two-tailed unpaired t-test, ****P < 0.0001). **j** Correlative light and electron microscopy images of N/TERT cells stably expressing mCherry-ADRP that were treated with ethanol (Control) or O/A for 2 hours, washed with PBS, and recovered in KSFM for 24 hours. N, nucleus. Scale bars, 1 µm. **k** Confocal images of live N/TERT cells that were pre-treated with DMSO or 20 µM T863 (DGAT1i) for 2 hours and then treated with ethanol (Control) or O/A for 2 hours in the continued presence of the same drug. Cells were then washed with PBS and recovered in KSFM containing DMSO or 20 µM DGAT1i for 24 hours, followed by staining with LipiBlue. Dotted lines indicate the cell boundaries. Scale bars, 10 µm. **l** Quantification of the total area of LD in each cell (labeled by LipiBlue) as shown in (**k**) (mean ± SEM, *n* = 75, 81, 69, 75 cells; data are pooled from three independent experiments; one-way ANOVA with Tukey’s multiple comparisons test, **P = 0.006, ****P < 0.0001, ns denotes not significant).

Given that key mitochondrial components, such as lipids, are subject to recycling following mitochondrial clearance by degradative lysosomes, we performed lipidomics analysis to examine potential changes in cellular lipid composition. N/TERT cells were treated with either ethanol (control) or O/A for 2 hours, allowed to recover for 24 hours, and then subjected to lipid extraction and analysis via lipidomics. Consistent with the results from the Cholesterol Glo^TM^-assay (Fig. 3f), cholesterol levels were significantly elevated. Interestingly, cholesterol ester (CE) levels were markedly reduced, suggesting enhanced CE hydrolysis, likely to make more free cholesterol available in response to elevated demand (Fig. 6a and Fig. S6a).

Lipidomics analysis also revealed a substantial increase in free fatty acids (FAs) (both short- and long-chain) in O/A-treated cells (Fig. 6b and Fig. S6a). Since free FAs, through their conversion to fatty acyl-CoA (FA-CoA), serve as major substrates for the synthesis of triacylglycerol (TAG) via the glycerol-3-phosphate (G-3-P) pathway, intermediates of this pathway were systematically analyzed (Fig. 6c). FA-CoA contributes to the synthesis of lysophosphatidic acid (LPA) and phosphatidic acid (PA). PA is subsequently dephosphorylated to generate diacylglycerol (DAG), which can also arise from monoacylglycerol (MAG) through monoacylglycerol acyltransferase (MGAT)-mediated acylation. Finally, DAG is converted to TAG via DGAT1 and DGAT2 and stored in lipid droplets. Unlike FAs, no major changes were observed in PA or MAG levels (Fig. 6d, e and Fig. S6a), while DAG levels were significantly reduced (Fig. 6f and Fig. S6a). Strikingly, a major increase in TAG across all chain lengths was detected (Fig. 6g and Fig. S6a). These results show that mitochondrial depolarization has a major impact on cellular lipid metabolism, leading to a dramatic accumulation of TAG.

Elevated levels of these lipids could induce lipotoxicity if unattended, requiring cells to sequester them through the formation of lipid droplets (LDs)^61–64^. To monitor the formation of LDs in N/TERT cells, we established N/TERT cells stably expressing mCherry-tagged adipose differentiation-related protein / perilipin-2 (ADRP) (mCherry-ADRP), a surface marker for LDs^64^. These N/TERT cells were treated with O/A for 2 hours, allowed to recover for 24 hours, and imaged. In agreement with the accumulation of TAG in N/TERT cells, as revealed by the lipidomics analysis, a robust increase in LD formation was observed under light microscopy and further confirmed via CLEM (Fig. 6h–j). Additionally, the accumulation of LDs in response to O/A was observed in other cell lines, including HeLa, U2OS, HEK293T, and NIH3T3 cells, using a number of LD detection methodologies, namely LipiRed/Blue or BODIPY (Fig. S6b–f). Finally, co-treatment of N/TERT cells with O/A and a DGAT1 inhibitor (DGAT1i) abolished the formation of LDs (Fig. 6k, l), indicating that the formation of LDs following mitochondrial depolarization by O/A is driven by DGAT1-mediated TAG generation.

Overall, these findings demonstrate that mitochondrial depolarization triggers a profound change in cellular lipid composition, leading to the accumulation of various lipid species, including cholesterol, FAs, and TAG. Importantly, TAG is sequestered into LDs, highlighting a mechanism for storing excessive lipids during mitochondrial clearance.

### Degradative capacity of lysosomes is essential for lipid droplet formation during the clearance of depolarized mitochondria

While our study suggests that the increase in cellular cholesterol levels following mitophagy is driven by enhanced cholesterol synthesis in the ER, the source of FA accumulation (which is coupled with the buildup of TAG) remains unclear. To address this issue, we performed a time-course analysis of LD formation and mitochondrial depolarization-induced mitophagy. To monitor LD formation, N/TERT cells were treated with O/A for 2 hours and imaged at different time points (0, 2, 4, and 6 hours) during recovery, using LipiRed (Fig. S7a, b). To monitor mitophagy, we established N/TERT cells that stably expressed a mitophagy reporter, TOM20 tagged with EGFP and mScarlet (TOM20-EGFP-mScarlet). TOM20-EGFP-mScarlet is targeted to mitochondria at steady state. During mitophagy, TOM20-EGFP-mScarlet, together with depolarized mitochondria, enters autophagosomes, which then fuse with lysosomes. In the acidic environment of the degradative lysosomes, EGFP loses its fluorescence whereas mScarlet continues to fluoresce. Changes in the mScarlet to EGFP ratio indicates the extent of mitophagy. N/TERT cells stably expressing TOM20-EGFP-mScarlet were treated with O/A for 2 hours, imaged at different timepoints (0, 2, 4, and 6 hours) during recovery, and the extent of mitophagy was monitored (Fig. S7c, d). Time-course analyses of the LD formation (Fig. S7a, b) and mitophagy (Fig. S7c, d) showed that mitophagy precedes LD formation (Fig. S7e). This temporal correlation suggests that LDs form following the release of lipids generated by the digestion of damaged mitochondria in degradative lysosomes.

**Fig. 7:**
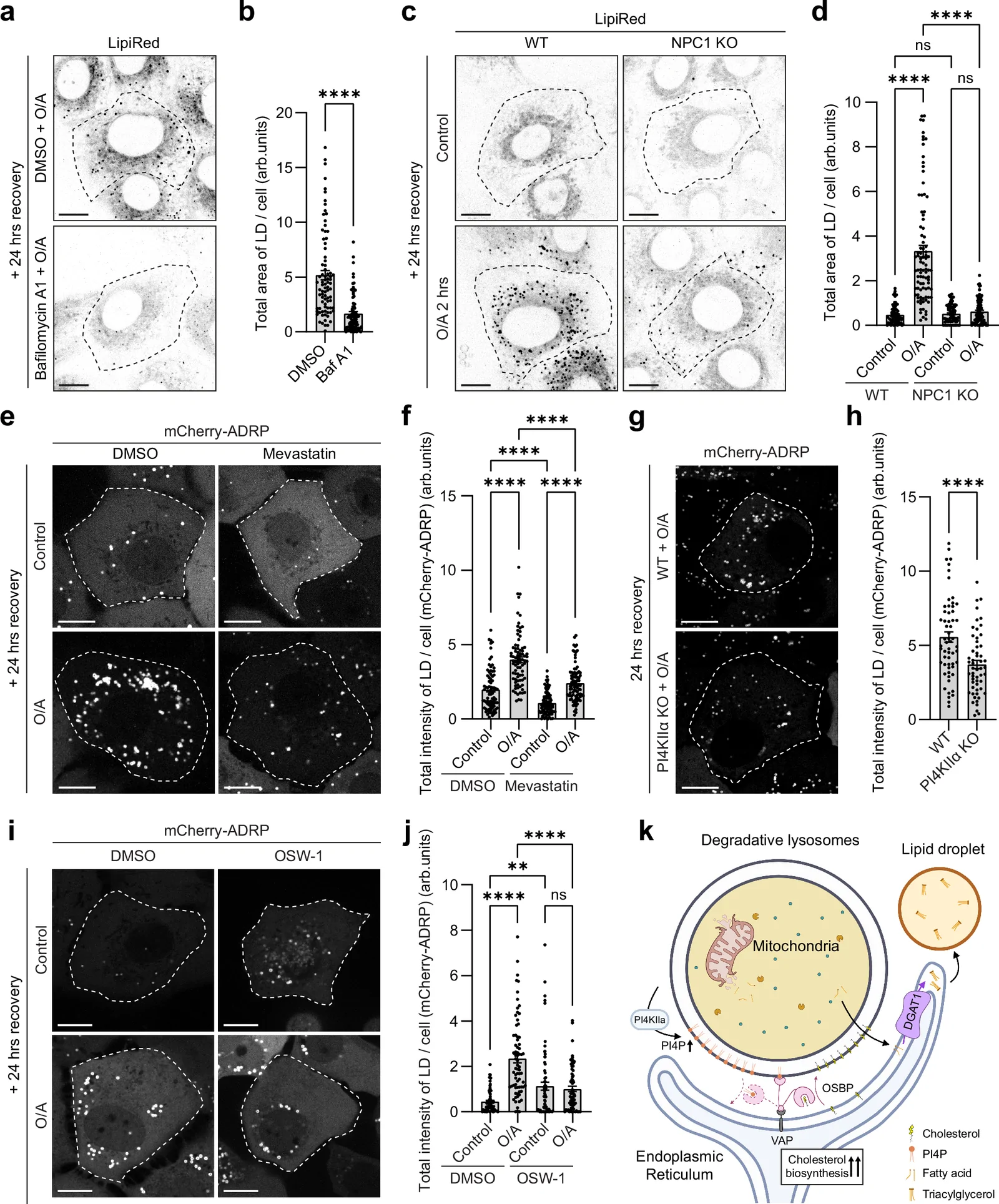
Degradative capacity of lysosomes is essential for lipid droplet formation during the clearance of depolarized mitochondria. **a** Confocal images of live N/TERT cells that were treated with O/A for 2 hours in the presence of DMSO or 100 nM bafilomycin A1 (BafA1), washed with PBS, and recovered in KSFM containing DMSO or BafA1 for 24 hours, followed by LipiRed staining. Dotted lines indicate the cell boundaries. **b** Quantification of lipid droplets (LDs) from (**a**) (*n* = 83 and 84 cells; Two-tailed unpaired t-test, ****P < 0.0001). **c** Confocal images of live wild-type (WT) or NPC1 knockout (KO) N/TERT cells that were treated with ethanol (Control) or O/A for 2 hours, PBS washed, and recovered in KSFM (24 hours), followed by LipiRed staining. **d** Quantification of LDs from (**c**) (*n* = 88, 91, 89, 87 cells; one-way ANOVA with Tukey’s multiple comparisons test, ****P < 0.0001, ns denotes not significant). **e** Confocal images of live N/TERT cells that were pre-treated with DMSO or 5 µM mevastatin for 16 hours and then treated with ethanol (Control) or O/A for 2 hours in the same drugs. Cells were then washed with PBS and recovered in KSFM containing DMSO or 5 µM mevastatin for 24 hours. **f** Quantification of LDs from (**e**) (*n* = 82, 84, 82, 85 cells; one-way ANOVA with Tukey’s multiple comparisons test, ****P < 0.0001). **g** Confocal images of live WT and PI4KIIα KO N/TERT cells treated with O/A for 2 hours, PBS washed, and recovered in KSFM for 24 hours. **h** Quantification of LDs from (**g**) (*n* = 59 and 54 cells; Two-tailed unpaired t-test, ****P < 0.0001). **i** Confocal images of live N/TERT cells pre-treated with DMSO or 20 nM OSW-1 for 2 hours and then treated with ethanol (Control) or O/A for 2 hours in the same drugs. Cells were then washed with PBS and recovered in KSFM containing DMSO or 20 nM OSW-1 for 24 hours. **j** Quantification of LDs from (**i**) (*n* = 75, 73, 73, 69 cells; one-way ANOVA with Tukey’s multiple comparisons test, **P = 0.006, ****P < 0.0001). **k** Cholesterol maintains the degradative capacity of lysosomes during clearance and recycling of dysfunctional mitochondria. Created in BioRender. Saheki, Y. (2026) https://BioRender.com/bt90qjl. All scale bars, 10 µm. All data in mean ± SEM and are pooled from three independent experiments.

To further investigate the requirement of degradative lysosomes in the formation of LDs, lysosomal pH was neutralized using bafilomycin A1 (BafA1) during O/A treatment. Consistent with the requirement of mitochondrial degradation in degradative lysosomes for LD formation, O/A-induced formation of LDs was significantly suppressed by co-treating cells with BafA1 (Fig. 7a, b). We also impaired the degradative function of lysosomes through genetic manipulation. Specifically, we used a CRISPR/Cas9-mediated gene-editing approach to knock out NPC1 (Fig. S7f), a protein essential for lysosomal cholesterol efflux. Its loss leads to cholesterol accumulation within the lysosomal lumen and disruption of lysosomal degradative functions that are required for effective autophagy and mitophagy^44,65,66^. NPC1 KO cells treated with O/A exhibited a marked reduction in LD formation, further confirming that the degradative function of lysosomes is a key requirement for LD biogenesis following mitochondrial depolarization (Fig. 7c, d).

Because efficient degradation of depolarized mitochondria and maintenance of lysosomal pH require cholesterol accumulation on degradative lysosomal membranes (Fig. 5a–d), we next examined whether cholesterol depletion impairs LD formation induced by mitochondrial depolarization. O/A-induced LD formation was markedly suppressed by mevastatin treatment in N/TERT cells, indicating that cholesterol is crucial for mitophagy-induced LD formation (Fig. 7e, f). Consistent with this result, treatment with UT-59, a SCAP inhibitor that blocks SREBP-2 cleavage and thereby inhibits cholesterol biosynthesis^67^, similarly suppressed O/A-induced LD formation (Fig. S7g, h). As a complementary approach, SREBP-2 expression was reduced using RNA interference (RNAi) (Fig. S8a). Treatment of cells with small interfering RNA (siRNA) targeting SREBP-2 for 72 hours resulted in significant suppression of both lysosomal cholesterol accumulation and LD formation induced by O/A treatment (Fig. S8a–e). These results further support the crucial role of SREBP-2-mediated cholesterol biosynthesis in mitophagy-induced LD formation. In a parallel experiment, HeLa cells were depleted of cholesterol by culturing them in lipoprotein-deficient serum (LPDS) together with mevastatin before O/A treatment. Compared with non-depleted control cells, cholesterol-depleted cells showed a strong reduction in O/A-induced LD formation (Fig. S8f, g). Together, these results suggest that cholesterol is required for LD formation during mitophagy and that this requirement is conserved across different cell types.

Finally, to test the role of PI4KIIα, and PI4P generated on lysosomal membranes by this kinase, in LD formation, we assessed LD formation in PI4KIIα KO cells. O/A-induced LD formation was markedly suppressed in PI4KIIα KO cells (Fig. 7g, h), supporting a model in which PI4P-driven delivery of ER cholesterol to lysosomes promotes efficient LD formation following mitophagy. Consistent with a role of PI4P-dependent activation of OSBP in driving cholesterol delivery to lysosomal membranes, pharmacological inhibition of OSBP by OSW-1 also reduced O/A-induced LD formation (Fig. 7i, j). Notably, treatment with OSW-1 alone modestly increased LD levels (Fig. 7i, j). This effect is consistent with the role of OSBP as a major lipid transfer protein that mediates cholesterol efflux from the ER, as OSBP inhibition likely leads to cholesterol accumulation in the ER, where excess cholesterol is esterified and stored in LDs. Similar results were obtained using siRNA targeting OSBP (Fig. S8a–e), supporting the crucial role of OSBP-mediated cholesterol transport in this process.

Taken together, these findings indicate that the primary source of FAs is the mitophagy-dependent breakdown of mitochondria-derived membrane lipids. While these results do not exclude the potential involvement of other mechanisms, including impaired β-oxidation, free FAs exported from degradative lysosomes likely contribute significantly to LD formation via their conversion to TAG (Fig. 6c).

In summary, these analyses reveal that the delivery of cholesterol from the ER to degradative lysosomes via PI4P-dependent activation of OSBP is essential for maintaining the degradative capacity of lysosomes, ensuring efficient clearance and recycling of dysfunctional mitochondria, which supports DGAT1-dependent LD formation to sequester the resulting FAs and TAG (Fig. 7k).

## Discussion

Our study uncovers cholesterol as a central player in coordinating the functional integrity of lysosomes during clearance and recycling of dysfunctional mitochondria. We found that mitophagy induces acute cholesterol delivery to degradative lysosomal membranes from the ER via PI4P-driven, OSBP-mediated cholesterol transport. This large efflux of cholesterol from the ER in turn activates SREBP-2, enhancing cholesterol production. Free FAs derived from degrading mitochondria are converted to TAG by DGAT1 and recycled and stored in newly formed LDs. Depletion of cholesterol impairs the functional integrity of lysosomes and reduces LD formation, highlighting the critical role of cholesterol in cellular homeostasis during organelle dysfunction (Fig. 7k). Our major findings are the following:

1. Using a highly sensitive cholesterol biosensor based on the GRAM domain of GRAMD1b (GRAM-W), we found that cholesterol accumulates on degradative lysosomal membranes upon mitochondrial damage.
2. We found that clearance of damaged mitochondria by degradative lysosomes is tightly coupled with the accumulation of PI4P on degradative lysosomal membranes via PI4KIIα activity. This, in turn, activates PI4P-driven, OSBP-mediated cholesterol transport, delivering cholesterol from the ER to the degradative lysosomal membranes.
3. This pool of cholesterol plays a critical role in maintaining the degradative capacity and membrane integrity of degradative lysosomes during the clearance of mitochondria. Accordingly, cholesterol depletion results in less efficient degradation of damaged mitochondria, lysosomal deacidification, and increased fragility of degradative lysosomes.
4. The efflux of cholesterol from the ER leads to upregulation of de novo cholesterol synthesis via enhanced SREBP-2 cleavage and the expression of its target genes, including HMGCR. Our experiments using cholesterol loading, SCAP KO, OSBP inhibition, and degron-induced OSBP degradation revealed that the activation of SREBP-2 is primarily driven by ER cholesterol depletion via OSBP-mediated cholesterol extraction and transport.
5. Using lipidomics, we found that mitochondrial depolarization-induced mitophagy results in a major change in cellular lipid composition, including increased levels of free FAs and TAG. This is accompanied by an increase in DGAT1-dependent LD formation. Inhibition of the degradative capacity of lysosomes, through neutralization of their pH, NPC1 knockout, or inhibition of cholesterol delivery to lysosomal membranes, suppresses LD formation.

Our findings suggest that accumulation of cholesterol on lysosomal membranes helps maintain lysosomal function and integrity. We find that this pool of cholesterol supports efficient degradative flux and lipid storage into LDs. In contrast, the accumulation of cholesterol within the lysosomal lumen has been reported in pathological states. For example, NPC1 deficiency, which leads to luminal cholesterol accumulation, impairs lysosomal integrity, degradative capacity, and mitophagy^66^. Moreover, luminal cholesterol accumulation is frequently associated with lysosomal dysfunction and the formation of multilamellar bodies (MLBs), which can be induced by mutations in GBA1^68,69^, overexpression of Mgat5 coupled with NPC1 inhibition^70^, or the loss of PLD3^71^. Together, these observations suggest that the site of cholesterol accumulation, whether at the limiting membranes or within the lumen, has profound functional implications for lysosomal homeostasis.

Previous studies have shown that cholesterol rapidly accumulates on lysosomal membranes following lysosomal membrane rupture through the phosphoinositide-initiated membrane tethering and lipid transport (PITT) pathway, facilitating membrane repair^45,46^. In contrast, we found that clearance of dysfunctional mitochondria is tightly coupled to cholesterol accumulation on lysosomal membranes without inducing lysosomal membrane damage. Mitochondria contain minimal amounts of cholesterol, making it unlikely that mitochondria-derived cholesterol is a major source of this new lysosomal cholesterol^23,72^. Instead, cholesterol accumulation is facilitated via activation of PI4P-driven, OSBP-mediated cholesterol transport from the ER to degradative lysosomes (like the PITT pathway). Our study further identifies a major upregulation of cholesterol biosynthesis through SREBP-2 cleavage/activation driven by OSBP-dependent depletion of ER cholesterol. Notably, we showed that SREBP-2 cleavage was also observed in cells upon lysosomal membrane rupture induced by LLOMe (Fig. S4c–f). Thus, the pathway identified in this study shares similarities with the PITT pathway, and the two may converge to maintain lysosomal functional integrity when lysosomes are stressed (e.g., conditions like cargo overload during mitophagy), regardless of the presence of membrane damage or rupture.

OSBP activity is tightly controlled by the levels of PI4P in cellular membranes^28–30,54,73^. PI4KIIα has previously been shown to localize to lysosomes to activate OSBP-related proteins (ORPs) in response to various conditions, including nutrient starvation-induced autophagy and LLOMe-induced membrane rupture^18,45,74^. We found that the knockout of PI4KIIα significantly, though not completely, suppressed the increase of PI4P on degradative lysosomal membranes during mitophagy (Fig. 2a, b). While our results confirm that PI4KIIα is the major kinase for PI4P synthesis in this process, we do not exclude the possibility that other enzymes, such as PI4KIIIβ^18,75,76^, may also contribute to the increase of PI4P on lysosomal membranes. Interestingly, while nutrient starvation has been reported to activate PI4KIIα on degradative lysosomes^18^, it did not induce major cholesterol accumulation on degradative lysosomes. These results suggest that the accumulation of cholesterol on lysosomal membranes occurs strongly during mitophagy, although we cannot exclude the possibilities that it might also occur in other forms of selective autophagy. Besides OSBP shown in this study, other LTPs involved in lysosomal membrane repair (e.g., ORP1L, ORP9, ORP10, ORP11, ATG2 and VPS13C^45,46,74,77^) may also play roles in maintaining the functional integrity of degradative lysosomes during clearance of dysfunctional mitochondria. Further studies are needed to understand how these LTPs may coordinate the transport of various lipids to lysosomes to maintain their degradative capacity.

In addition to the increase of cholesterol on lysosomal membranes, we also observed some increase of cholesterol at the PM, as assessed by EGFP-GRAM-W, following O/A treatment (Fig. 1a). Notably, this increase in PM cholesterol was not suppressed by OSBP inhibition (Fig. 2h), suggesting that such cholesterol accumulation at the PM is mediated by OSBP-independent mechanisms. This process may also contribute to the depletion of ER cholesterol and the subsequent activation of SREBP-2. Future studies will be required to elucidate the molecular mechanisms underlying this process.

Proper maintenance of lysosomal pH is essential for the degradative properties of lysosomes. Nutrient starvation-induced autophagy is coupled with acidification of lysosomes and increased lysosomal degradative capacity^18,78^. Our study identifies cholesterol as a key factor in maintaining lysosomal pH, as depletion of cellular cholesterol led to lysosomal deacidification (Fig. 5c, d). A recent study reported that cholesterol is important for stabilizing V-ATPase assembly at the TGN, which is necessary for maintaining its pH^57^. Similarly, cholesterol may help stabilize lysosomal V-ATPase to preserve lysosomal acidification during the clearance of dysfunctional mitochondria. Future studies should examine V-ATPase localization and function in O/A-treated cells in the presence or absence of cholesterol. Another factor important for maintaining the degradative capacity of lysosomes is lysosomal Ca^2+^ homeostasis. Growing evidence suggests that Ca^2+^ dysregulation can impair endolysosomal function^79–82^. Disruption of the Ca^2+^ gradient across lysosomal membranes may alter the electrochemical balance required for proton influx, thereby reducing the efficiency of lysosomal acidification^83^. However, defective lysosomal Ca^2+^ homeostasis has also been reported to occur in the absence of lysosomal pH defects^84^. Whether disruptions in cholesterol homeostasis affect lysosomal Ca^2+^ regulation remains an important question for future investigation. Importantly, cholesterol-rich membranes are more rigid and resistant to mechanical stress^85^. Accordingly, cholesterol depletion impaired the integrity of lysosomal membranes, as evidenced by their increased sensitivity to LLOMe. Altogether, cholesterol accumulation on degradative lysosomal membranes may reflect a requirement for this lipid in maintaining the degradative capacity and membrane integrity of lysosomes upon heightened digestive demand. Sustained cholesterol accumulation may also serve to protect lysosomes from rupture during the rapid membrane expansion caused by the influx of large cargo, such as damaged mitochondria.

A previous study reported that DGAT1-dependent LDs form during iron depletion, and that these LDs promote mitophagy^50^. During amino acid starvation-induced autophagy, LDs form in a DGAT1-dependent manner to preserve mitochondrial function^62^. Our results instead suggest that the degradation of dysfunctional mitochondria is a key cellular mechanism by which lipids, including FAs, are recycled and then converted to TAGs by DGAT1 for LD formation. In this way excess lipids are effectively stored, potentially suppressing lipotoxicity. Hence, LDs play a critical role in cellular stress adaptation^86,87^. Notably, despite an increase in LDs, cholesterol esters (CEs), another major LD component, are significantly reduced. This suggests that stored CEs undergo active hydrolysis or lipophagy to release free cholesterol to further maintain the functional integrity of lysosomes. In the future, it would be informative to track the movement of mitochondria-derived lipids during mitochondrial degradation using organelle-specific labelling approaches to gain further insights into the mechanisms of lipid recycling and/or storage.

The pathogenesis of neurodegenerative disorders is increasingly associated with the breakdown of inter-organelle communication^88,89^. Our findings highlight the critical importance of coordinated lipid transfer reactions at ER-lysosome contact sites in maintaining the degradative capacity of lysosomes for proper clearance of damaged mitochondria and subsequent recycling of their lipids into LDs. Failure of this process could potentially underlie neurodegeneration as impaired clearance of damaged mitochondria, lysosomal dysfunction, and defective LD formation have all been linked to various neurodegenerative disorders^3,90–92^. For example, dysfunction of key regulators of mitophagy, including PINK1 and Parkin, is tightly linked to PD^89,93–95^. Growing evidence suggests that lysosomal abnormalities have been associated with AD^96^, PD^92,97,98^, amyotrophic lateral sclerosis (ALS)^98^, and hereditary spastic paraplegia (HSP)^99^. Likewise, dysregulated cholesterol homeostasis and abnormal LD formation have been linked to PD and AD^91,100–105^. Finally, the dysfunction of the proteins involved in lipid transfer and / or the maintenance of ER-lysosome contact sites, including VPS13C, VAPB, and Protrudin, is implicated in several neurodegenerative disorders, including ALS^106–108^, HSP^109,110^, and PD^77,111,112^. Interestingly, accumulation of amyloid precursor protein, a primary driver of plaque formation in AD brain, has been reported to disrupt ER-late endosome/lysosome contact sites, suggesting that the instability of these contact sites may be an early event in AD^81^. Collectively, these findings underscore the vital role of inter-organelle communication at membrane contact sites in the maintenance of brain health.

In sum, our findings reveal cholesterol as a central regulator of lysosomal functional integrity and LD formation during clearance and recycling of dysfunctional mitochondria, offering important insights into cellular mechanisms that enable adaptation to organelle dysfunction.

## Methods

### Antibodies and chemicals

Primary and secondary antibodies, chemicals and other reagents used in this study are listed in Supplementary Table 2.

### DNA plasmids

DNA plasmids, the sequences of oligos and primers used are listed in Supplementary Table 2.

#### Cloning of pLJM1-EGFP-BlastR

cDNA of Blasticidin deaminase was digested from pLJC6-3XHA-TMEM192 and ligated into pLJM1-EGFP at BamHI and KpnI sites.

#### Cloning of pLJM1_EF1alpha_EGFP_BlastR

EF1α promoter sequence was amplified from pSH-EFIRES-B-Seipin-miniIAA7-mEGFP by PCR using the following primer set (NdeI-EF1alpha_F and EF1alpha-NheI_R). PCR product was ligated into pLJM1-EGFP-BlastR at NdeI and NheI sites.

#### Cloning of pLJM1_EF1alpha_dTomato-ORP9-PH_BlastR

cDNA encoding dTomato-ORP9-PH was digested from pCMV-dTomato-ORP9-PH and ligated into pLJM1_EF1alpha_EGFP_BlastR at EcoRI and XhoI sites.

#### Cloning of pLJM1-EF1alpha_mCherry-ADRP

cDNA of ADRP was digested from pEGFP-C1-ADRP and ligated into mCherry-C1 at NheI and EcoRI sites to generate cDNA encoding mCherry-ADRP. The mCherry-ADRP cDNA was then digested and ligated into pLJM1_EF1alpha_EGFP_BlastR at EcoRI and XhoI sites.

#### Cloning of pLJM1_EF1alpha_TOM20-EGFP-mScarlet_In-Fusion

cDNA of EGFP was amplified from pEGFP-C1 and ligated into pLJM1_EF1alpha_TOM20-mScarlet using In-Fusion® Snap Assembly Master Mix (Takara Bio) to generate cDNA encoding TOM20-EGFP-mScarlet.

#### Cloning of pLJM1_EF1alpha_LAMP1-miRFP703_BlastR and pLJM1_EF1alpha_TOM20-mScarlet_BlastR

cDNA encoding LAMP1-miRFP703 was digested from pLAMP1-miRFP703 and ligated into pLJM1_EF1alpha_EGFP_BlastR at NheI and EcoRI sites, generating pLJM1_EF1alpha_LAMP1-miRFP703_BlastR plasmid. cDNA encoding signal sequence of TOM20 was then amplified from TOM20-mCherry-GAI (1-92) by PCR using the following primer set (NheI-TOM20 F and TOM20-EcoRI R). PCR product was ligated into pLJM1_EF1alpha_LAMP1-703_BlastR plasmid at NheI and EcoRI sites, generating pLJM1_EF1alpha_TOM20-miRFP703_BlastR plasmid. cDNA of mScarlet was digested from pmScarlet_C1 and ligated into the TOM20-miRFP703 plasmid at EcoRI and SalI sites.

#### Cloning of pLJM1_EF1alpha_BFP-Sec61b_Blast

cDNA encoding sequence of BFP-Sec61β was amplified from BFP-Sec61 beta by PCR using the following primer set (5ex-NheI-kozak-BFP-Sec61b and 5ex-EcoRI-BFP-Sec61b). PCR product was ligated into pLJM1_EF1alpha_EGFP_Blast plasmid at NheI and EcoRI sites.

#### Cloning of pLJM1_EF1alpha_BFP-Parkin and pLJM1_EF1alpha_EGFP-FAPP1-PH_Blast

pLJM1_EF1alpha_EGFP_Blast plasmid was linearized by PCR amplification using the primer set (KM3-Gib-EcoRI-F and KM3-Gib-NheI-R). The PCR product was ligated with DNA sequences (IDT gBlock) of BFP-Parkin and EGFP-FAPP1-PH using GenBuilder™ Plus Cloning Kit (GenScript, L00744-10).

### Cell culture and transfection

HeLa (Gift from Pietro De Camilli), HEK293T (Gift from Nguan Soon Tan), U2OS (Gift from Wenting Zhao), and NIH3T3 (Gift from Lu Lei) cells were cultured in Dulbecco’s modified Eagle’s medium (DMEM) containing 10% fetal bovine serum (FBS) and 1% penicillin/streptomycin at 37 °C and 5% CO_2_. Keratinocytes (N/TERT-1 cells) (Gift from James G. Rheinwald)^113^ were cultured in Keratinocyte serum-free medium (KSFM) supplemented with 300 µM calcium chloride, human recombinant epidermal growth factor (rEGF), and bovine pituitary extract (BPE) as prepared according to manufacturer protocol, and 1% penicillin/streptomycin at 37 °C and 5% CO_2_. Transfection of plasmids was carried out with Lipofectamine™ 2000 (Thermo Fisher Scientific) 1 day before imaging. All cell lines were routinely verified as free of mycoplasma contamination at least every two months, using MycoGuard Mycoplasma PCR Detection Kit (Genecopoeia) or MycoStrip™ (Invivogen). No cell line used in this study was found in the database of commonly misidentified cell lines that are maintained by ICLAC and NCBI Biosample.

### siRNA-mediated knockdown in N/TERT cells

For siRNA-mediated knockdown of OSBP and SREBP-2, 6 × 10^5 ^N/TERT cells stably expressing EGFP-GRAM-W and mCherry-ADRP were suspended in P3 primary cell nucleofector solution and supplement (Lonza) together with either 30 pmol of non-targeting control siRNAs (siNC-1 from IDT), siRNAs against OSBP (hs.Ri.OSBP.13.1, hs.Ri.OSBP.13.2, and hs.Ri.OSBP.13.3 from IDT), or siRNAs against SREBP-2 (hs.Ri.SREBF2.13.1, hs.Ri.SREBF2.13.2, and hs.Ri.SREBF2.13.3 from IDT). Electroporation was carried out using the program “DS-138”. Cells were plated and experiments carried out after 72 hours. Knockdown of each target gene was confirmed by immunoblotting.

### Generation of CRISPR/Cas9-edited cell lines

Generation of SCAP KO HeLa cells, NPC1 KO N/TERT cells stably expressing EGFP-GRAM-W (N/TERT GW), and PI4KIIα KO N/TERT GW cells stably expressing EGFP-GRAM-W (N/TERT GW).

SCAP knockout (KO) HeLa cells, NPC1 KO N/TERT GW cells, and PI4KIIα KO N/TERT GW cells were generated using the Alt-R CRISPR-Cas9 System (IDT) and electroporation by the nucleofector system (Lonza) according to the manufacturer’s protocol. In brief, CRISPR gRNAs targeting SCAP genomic sequences, CTCACGCAGCCTTTCAGTCA (SCAP gRNA1) and CAAGGGCTCCATAGTGGTTA (SCAP gRNA2), CRISPR gRNAs targeting NPC1 genomic sequences, ACGCCTGTAATGCCACCAAC (NPC1 gRNA for exon5) and TACTGCGTGTTCGTCAGGCC (NPC1 gRNA for exon8), and CRISPR gRNAs targeting PI4K2α genomic sequences, CCCCGGGCCCGATCCAACAG (PI4K2A gRNA1) and CCAGGGCCAGACCCAAACCG (PI4K2A gRNA2) were designed based on previous studies^45,114^. CRISPR Cas9 ribonucleoprotein (RNP) complexes consisting of the sgRNAs (SCAP: SCAP gRNA1 and SCAP gRNA2; NPC1: NPC1 gRNA for exon 5 and NPC1 gRNA for exon 8; PI4K2α: PI4K2A gRNA1 and PI4K2A gRNA2), and Cas9 nucleases (IDT) were added to 6.5 × 10^5^ HeLa cells or N/TERT GW cells that were suspended in P3 primary cell nucleofector solution and supplement (Lonza). Electroporation was carried out using the program “CN-114” for HeLa cells, and the program “DS-138” for N/TERT GW cells. Either electroporated HeLa cells or N/TERT GW cells suspension were then plated, and single cell clones were isolated using BD FACSAria™ Fusion (BD Biosciences). KO of each target genes was confirmed by immunoblotting.

#### Generation of endogenously tagged HeLa cell lines

Endogenous tagging of HeLa cells was carried out using the ORANGE system^115^. In brief, cDNA corresponding to FKBP12_F36V_ and mScarlet-I fusion construct with a linker region was cloned into the HindIII and XhoI sites of pORANGE_intermediate_OSBP_2 (TN353), which was constructed in our previous study^30^, to generate pORANGE_FKBP12_F36V-mScarlet-I-OSBP_site_2. 5 days after transfection of pORANGE_FKBP12_F36V-mScarlet-I-OSBP_site_2, cells were imaged under spinning disc confocal (SDC) microscopy. Single fluorescent positive cells were then isolated using BD FACSAria™ Fusion (BD Biosciences) based on the expression of mScarlet-I. Expression of endoFKBP12_F36V_-mScarlet-I-OSBP was subsequently confirmed by microscopy and immunoblotting.

### Generation of N/TERT cell lines via lentiviral transduction

To generate lentiviruses, lentiviral helper plasmids (pMD2.G, pRSV-REV, and pMDLg/pRRE) (3.7 µg each) were transfected using Lipofectamine™ 2000 into 4.4 × 10^6^ HEK293T cells together with one of the following: pLJM1_EF1alpha_BFP-Sec61b_Blast, pLJM1_EF1alpha_dTomato-ORP9-PH_BlastR, pLJM1_EF1alpha_EGFP-FAPP1-PH_Blast, pLJM1_EF1alpha_LAMP1-miRFP703_BlastR, pLJM1_EF1alpha_TOM20-EGFP-mScarlet_In-Fusion, pLJM1_EF1alpha_TOM20-mScarlet_BlastR, pLJM1_EF1alpha_BFP-Parkin, pLJM1-EF1alpha_mCherry-ADRP_BlastR, pLJM1-FIRE-pHLy (7.4 µg each). Supernatant was collected 48 hours after transfection and filtered using a 0.45 µm filter unit to recover lentiviruses. Either N/TERT cells stably expressing EGFP-GRAM-W, wild-type N/TERT cells, or PI4KIIα KO N/TERT cells stably expressing EGFP-GRAM-W were seeded at 1.5 × 10^5^ cells and transduced with lentiviruses to generate N/TERT cells stably expressing EGFP-GRAM-W and BFP-Sec61β, EGFP-GRAM-W and dTomato-ORP9-PH, EGFP-GRAM-W together with dTomato-ORP9-PH and LAMP1-miRFP703, EGFP-GRAM-W and LAMP1-miRFP703, EGFP-GRAM-W and mCherry-ADRP, EGFP-GRAM-W and TOM20-mScarlet, BFP-Parkin, EGFP-FAPP1-PH and LAMP1-miRFP703, mTFP1-LAMP1-mCherry, or TOM20-EGFP-mScarlet, and PI4KIIα KO N/TERT cells stably expressing EGFP-GRAM-W and dTomato-ORP9-PH. After blasticidin or puromycin selection and cell sorting using flow-cytometry based on each fluorescence, protein expression was confirmed by microscopy.

### Live cell imaging and immunofluorescence with fluorescence microscopy

For imaging experiments, cells were plated onto 35 mm glass-bottom dishes at low density (MatTek Corporation).

#### Fixed cells

Cells were fixed with 4% formaldehyde solution (diluted from 16% Formaldehyde, Thermo Fisher Scientific, in PBS), washed in phosphate-buffered saline (PBS), and neutralized with PBS containing 50 mM glycine. For LC3B staining, cells were additionally fixed with ice-cold ethanol for 10 minutes. Cells were then permeabilized with PBS containing 0.1% Saponin and 1% bovine serum albumin (BSA), immunostained with designated antibodies or stained with 4’,6-diamidino-2-phenylindole (DAPI) in the same buffer and maintained in PBS. Primary antibodies were incubated at room temperature (RT) for 1 hour followed by incubation with Alexa Fluor-conjugated secondary antibodies at RT for 45 minutes. For filipin staining, fixed cells were stained with filipin in PBS without permeabilization. Fixed cell samples were imaged by SDC microscopy. Images from a mid-focal plane are shown.

#### Live cells

Cells were transfected 1 day before imaging. For detecting lipid droplets, cells were treated with 100 nM LipiBlue or 1 μM LipiRed for 30 minutes before the imaging. Cells were washed once before imaging with an SDC microscope. All types of microscopies were carried out at 37 °C.

Spinning disc confocal (SDC) microscopy (Figs. 1a, 2e, h, 3g, 4h, 5c, 6h, k, 7a, c, e, g, i, Figs. S1a, S6b–f, S7a, c, g, S8b, d, f) and SDC-structured illumination microscopy (SDC-SIM) (Figs. 1c–g, 2a, c, g, j, 5e, 6j, Figs. S1b, c, e–g, i, j, S2a, b, e, f, h and S5b) were performed on a setup built around a Nikon Ti2 inverted microscope equipped with a Yokogawa CSU-W1 confocal spinning head, a Plan-Apo objective (100×1.45-NA), a back-illuminated sCMOS camera (Prime 95B; Photometrics). Excitation light was provided by 405-nm/100 mW (Vortran) (for LipiBlue/ Filipin), 488-nm/150 mW (Coherent) (for GFP), 561-nm/100 mW (Coherent) (for dTomato/ mCherry/ mScarlet/ LipiRed/ Alexa 594) and 642-nm/110 mW (Vortran) (for miRFP703/Alexa 647) (power measured at optical fiber end) DPSS laser combiner (iLAS system; Gataca systems), and all image acquisition and processing were controlled by MetaMorph (Molecular Device) software. Images were acquired with exposure times in the 500 msec range.

### Correlative light and electron microscopy (CLEM)

N/TERT cells stably expressing EGFP-GRAM-W and TOM20-mScarlet were cultured on 35 mm gridded glass-bottom dishes (P35G-1.5-14-C-GRD, MatTek Corp., Ashland, MA) at very low density (3000-4000/dish) 3 days prior to treatment. Cells were treated with ethanol for 2 hours, or 10 μM oligomycin and 4 μM antimycin A (O/A) for 2 hours, or treated with O/A for 2 hours, washed with PBS, and recovered in KSFM for 24 hours, followed by staining with 50 nM LysoTracker™ Deep Red for 30 mins. Cells were then fixed with 4% formaldehyde in PBS for 15 minutes at RT and washed 2 times with PBS.

For light microscopy imaging, cells were imaged using a SDC-SIM with a 100x objective. Multidimensional image stacks were acquired using 488 nm, 561 nm, and 642 nm laser excitations at each z-plane with a step size of 0.2 µm.

Following confocal imaging, electron microscopy imaging was performed by Electron Microscopy Unit, National University of Singapore. The cells were further fixed with 2.5% glutaraldehyde (EMS) in PBS (pH 7.4) at 4 °C for 2 hours. Samples were processed using the osmium-thiocarbohydrazide-osmium (OTO) protocol, which included en bloc staining with 1% uranyl acetate in ultrapure water overnight at 4 °C. Subsequently, samples were stained with lead aspartate and dehydrated in a cold graded ethanol series (25%, 50%, 75%, 95%) for 5 min each, followed by two exchanges in 100% anhydrous ethanol (EMS) for 10 min each. Samples were then infiltrated with Araldite resin: Ethanol gradient (1:1, 3:1) for 1 hour each and Araldite resin: Ethanol gradient (6:1) for overnight. Samples were exchanged into fresh resin and incubated at 40 °C, 45 °C, 50 °C oven for 1 hour each. Resin-embedded samples were polymerised at 60 °C for 24 hours. Serial ultrathin sections (60 nm) were sectioned and mounted onto formvar and carbon coated 2 × 1 mm slot grids (EMS). Transmission electron microscopy (TEM) was performed using a Talos L120 (Thermo Scientific) operated at 100 kV. Electron micrographs were captured using a CETA 16 M 4 K CMOS camera. Correlation of confocal and EM images were carried out using ImageJ or Imaris Software (Oxford Instruments).

### Image analysis

All images were analyzed off-line using ImageJ (http://fiji.sc/wiki/index.php/Fiji). Statistics of quantified fluorescence signals were performed using Excel (Microsoft) and Prism 11 (GraphPad Software). All data are presented as mean ± SEM. In dot plots, each dot represents a value from a single cell with the bar as the mean.

For analysis of the number of cholesterol-rich vesicles via SDC microscopy (Figs. 1b, 2f, i, 3h and Fig. S8c), background subtraction was performed by subtracting two times the mean cytosolic intensity from the entire image of EGFP-GRAM-W to remove non-vesicular background signals. The number of vesicles remaining in the cells were manually counted and plotted.

For line-scan analysis of EGFP-GRAM-W, TOM20-mScarlet, α-LC3B, and α-LAMP1 signals on vesicular structures (Fig. 1c, d), a previously described protocol was followed^116^. Briefly, a line was manually drawn between the arrowheads. Normalized fluorescence intensity profiles for each channel were extracted along this line to assess the spatial distribution of the respective signals.

For analysis of lysosomal intensity of mCherry-D4H (Fig. S1d), an intensity threshold was applied to each image of α-LAMP1 to generate binary masks for the lysosomal regions. The mean fluorescence intensity of mCherry-D4H within the LAMP1-defined mask was measured after background subtraction and plotted.

For analysis of the recruitment of EGFP-GRAM-W to the vesicular structures (Fig. S1h), an intensity threshold was applied to each image to segment the cytosolic EGFP-GRAM-W-accumulating regions in respective cell lines. The total fluorescence intensities of EGFP-GRAM-W after background subtraction at the segmented region in the cytosol were measured and plotted (pixel size cut-off: 5-infinity).

For analysis of lysosome size via SDC microscopy (Fig. 1h), a Mexican hat filter was applied to each image of α-LAMP1, and the diameter of the largest lysosome in each cell was manually measured and plotted.

For analysis of the recruitment of dTomato-ORP9-PH to lysosomes via SDC microscopy (Fig. 2b), an intensity threshold was applied to each image of α-LAMP1 to generate binary masks for the lysosomal regions. The mean fluorescence intensity of dTomato-ORP9-PH within the LAMP1-defined mask was measured after background subtraction and plotted.

For analysis of lysosomal EGFP-GRAM-W and dTomato-ORP9-PH levels over time (Fig. 2d), an intensity threshold was applied to each image of LAMP1-miRFP703 to generate binary masks for the lysosomal regions. The mean fluorescence intensities of EGFP-GRAM-W and dTomato-ORP9-PH within the LAMP1-defined mask were measured after background subtraction and normalized using the intensity values at 0 minutes before being plotted.

For analysis of the recruitment of OSBP to lysosomes via SDC microscopy (Fig. 2k and Fig. S2g), an intensity threshold was applied to each image of α-LAMP1 to generate binary masks for the lysosomal regions. The mean fluorescence intensity of α-OSBP within the LAMP1-defined mask was measured after background subtraction and plotted.

For analysis of the number of PI4P-rich vesicles via SDC microscopy (Fig. S2c), the number of identifiable vesicles coated with EGFP-FAPP1-PH were manually counted and plotted.

For analysis of the fluorescence ratio between mTFP1 and mCherry using FIRE-pHLy (Fig. 5d), an intensity threshold was applied to each channel to generate independent binary masks for mTFP1 and mCherry. The mean fluorescence intensities of mTFP1 and mCherry within their respective masks were measured after background subtraction. For each cell, the ratio of mean mTFP1 intensity over mean mCherry intensity was calculated and plotted.

For analysis of the presence of galectin-3 on lysosomes via SDC microscopy (Fig. 5f), an intensity threshold was applied to each image of α-LAMP1 to generate binary masks for the lysosomal regions. The mean fluorescence intensity of α-Gal-3 within the LAMP1-defined mask was measured after background subtraction and plotted.

For analysis of the formation of lipid droplets via SDC microscopy (Figs. 6i, l, S6b–e, 7b, d, f, h, j, Fig. S7b, h, S8e, g), an intensity threshold was applied to each image of mCherry-ADRP, LipiBlue, or LipiRed after background subtraction to generate binary masks for the lipid droplet regions in HeLa, N/TERT, HEK293T, NIH3T3, and U2OS cells. The total area covered by LipiBlue or LipiRed masks, or the total intensity of mCherry-ADRP in each cell were measured and plotted.

For analysis of the fluorescence ratio between mScarlet and EGFP using TOM20-EGFP-mScarlet (Fig. S7d), an ImageJ plugin “mito-QC Counter” was used^117^. Briefly, an intensity threshold was applied to each channel to generate independent binary masks for mScarlet and EGFP. The mean fluorescence intensities of mScarlet and EGFP within their respective masks were measured after background subtraction. For each cell, the ratio of mean mScarlet intensity over mean EGFP intensity was calculated and plotted.

For non-linear regression analysis between lipid droplet formation and mitophagy (Fig. S7e), the mean values of the lipid droplet index (shown in Fig. S7b) and the mitophagy index (shown in Fig. S7d) were normalized to a 0–1 scale. A four-parameter logistic (4PL) variable slope curve fit was performed for each experimental group. Normalized data were plotted, and fitted curves were used to evaluate the relationship between lipid droplet formation and mitophagy.

### Biochemical analyses

#### Immunoblotting

HeLa cells and N/TERT cells were lysed in buffer containing 2% sodium dodecyl sulfate (SDS), 150 mM NaCl, 10 mM Tris (pH 8.0), and incubated at 60 °C for 20 minutes followed by additional incubation at 70 °C for 10 minutes. For immunoblotting against NPC1 and MTCO2, lysates were incubated at 37 °C for 20 minutes. The lysates were treated with benzonase nuclease (Sigma-Aldrich/Merck or SantaCruz) for 10–15 minutes at RT. The bicinchoninic acid assay (BCA assay) kit (Thermo Fisher Scientific) and Pierce™ Dilution-Free™ Rapid Gold BCA Protein Assay (Thermo Fisher Scientific) were used to measure protein concentration. Cell lysates were processed for SDS-PAGE and immunoblotting with standard procedure. All immunoblottings were developed by chemiluminescence using the SuperSignal West Dura reagents (Thermo Fisher Scientific). For the quantification of each protein level, the intensity of chemiluminescence signals of the protein of interest was measured and plotted as indicated.

#### Cholesterol measurement with Cholesterol/Cholesterol Ester-Glo™ assay kit

N/TERT cells were seeded in 10 cm dishes and cultured for two days prior to treatment. Cells were treated with either ethanol or O/A for 2 hours, followed by recovery in KSFM for 24 hours. Cells were detached using Accutase (Gibco) for 10 minutes at 37 °C, pelleted by centrifugation, and washed twice with ice-cold PBS. A total of 100,000 cells per condition were used for cholesterol quantification with the Cholesterol/Cholesterol Ester-Glo™ Assay Kit (Promega, J3190), according to the manufacturer’s instructions. Luminescence was measured using a microplate reader controlled by Gen5 3.09 software (BioTek) and the values are plotted.

### Lipidomics

Lipidomics analysis was performed by Singapore Phenome Center.

#### Sample preparation

225 µL of -20 °C pre-cooled methanol containing mixed internal standards (for internal standards information, see Supplementary Table 3) was added to cell samples and vortexed for 10 seconds. Add 750 µL -20 °C pre-cooled methyl tert-butyl ether (MTBE) and centrifuged at 20,000 x g for 10 minutes at 4 °C. The upper layer was taken out for drying and reconstituted in 80 µL dichloromethane/methanol (1:1) for LC-MS analysis (C18 and HILIC column).

500 µL of -20 °C pre-cooled isopropyl alcohol (IPA) (containing mixed internal standards) was added to cell samples and vortexed for 10 seconds. Add 88 µL water and centrifuged at 20,000 x g for 10 minutes at 4 °C. The upper layer was taken out for drying and reconstituted in 36 µL dichloromethane/methanol (1:1) with 0.1% formic acid for LC-MS analysis (EC C18 column).

Quality control (QC) was prepared by pooled sample from all samples, and six QCs were analyzed along with the samples in each batch to ensure the reliability of the method and the instrument stability.

#### LC-MS analysis

The samples were analyzed on quadrupole ion-trap-ABSciex QTRAP 6500 (SCIEX, Framingham, MA, USA) mass spectrometer with an ESI source, and using the multiple reaction monitoring (MRM) mode in both positive mode and negative mode. The instrument was connected in line with the liquid chromatography system (Agilent 1290 Infinity II). Mass spectrometry instrument parameters were as follows: the curtain gas (CUR) value was set at 40 psi; the collision gas (CAD) setting was medium; the ionspray voltage was ±4500 V and the source temperature was at 350 °C. The pressure for nebulization gas (GS1) and drying gas (GS2) were both set at 55 psi. The entrance potential (EP) was ±10 V and collision cell exit (CXP) was 10 V. For each group (Ethanol, and O/A), data were obtained from three independent experiments.

The MTBE extract was separated with Phenomenex Kinetex C18 (2.1*100 mm, 2.6 μm) (Stream2) and Waters BEH HILIC (2.1*100 mm, 1.7 μm) (Stream1) at 45 °C with the mobile phase (A1) water/acetonitrile (5:95, v/v) with 10 mM ammonium acetate and (B1) water/acetonitrile (1:1, v/v) with 10 mM ammonium acetate and 0.01% ammonia-water (28%); (A2) water/acetonitrile/methanol (1:1:1, v/v/v) with 7 mM ammonium acetate and (B2) IPA with 7 mM ammonium acetate.

The IPA extract was separated with Agilent Zorbax Eclipse Plus C18 (2.1 × 100 mm, 1.8 μm) at 50 °C with the mobile phase (A) water/acetonitrile/methanol (1:1:1, v/v/v) with 0.1% formic acid and 1 mM ammonium acetate and (B) IPA/acetonitrile (90:10, v/v) with 0.05% formic acid and 10 mM ammonium acetate.

Raw peak areas of the detected lipid species were normalized to the internal standard for each lipid class to account for instrument drift. All samples were analyzed in randomized order. The peak areas of the metabolites were integrated by SCIEX OS software (Version 1.4, AB Sciex Corporation, Framingham, MA, United States).

### mRNA-seq

5 × 10^5 ^N/TERT cells were plated onto 60 mm dishes two days prior to RNA extraction and cultured in KSFM supplemented with rEGF, BPE and 1% penicillin/streptomycin at 37 °C and 5% CO_2._ RNeasy mini kit (Qiagen) was used for the purification of total RNA from the cells. mRNA-seq and data analysis were performed by Genewiz / Azenta Life Sciences. For each group (Ethanol, and O/A), data were obtained from four independent experiments. In brief, 1 μg total RNA was used for library preparation and subsequent mRNA-seq experiments. mRNA isolation was performed using Oligo(dT) beads, and the mRNA fragmentation was performed using divalent cations and high temperature. First strand cDNA and second-strand cDNA were synthesized using random primers. The purified double-stranded cDNA was then treated to repair both ends and add a dA-tailing in one reaction, followed by a T-A ligation to add adaptors to both ends. Size selection of Adaptor-ligated DNA was then performed using DNA Clean Beads. Each sample was then amplified by PCR using P5 and P7 primers and the PCR products were validated. Then libraries with different indexes were multiplexed and loaded on an Illumina NovaSeq X 25B instrument for sequencing using a 2×150 paired-end (PE) configuration according to manufacturer’s instructions. Obtained data were cleaned and aligned to reference genome (Homo sapiens, Ensembl, GRCh38.p13) via software HISAT2 (v2.0.1). HTSeq (v0.6.1) was used to estimate gene and isoform expression levels from the pair-end clean data. Finally, DESeq2 Bioconductor package was used to perform differential expression analysis. The estimates of dispersion and logarithmic fold changes incorporate data-driven prior distributions, *P*adj of genes was set <= 0.05 to detect differential expressed ones.

### Statistics and reproducibility

No statistical method was used to predetermine sample size, and the experiments were not randomized for imaging. Sample size and information about replicates are described in the figure legends. All experiments were independently conducted at least 3 times to confirm reproducibility. The number of biological replicates is shown as the number of independent experiments within figure legends for each figure. Comparisons of data were carried out by the two-tailed unpaired Student’s t-test, Chi-square test and Odds Ratio test, one-way ANOVA followed by Tukey’s or Dunnett’s multiple comparisons, or two-way ANOVA followed by Šídák’s multiple comparisons as appropriate with Prism 11 (GraphPad software). Unless *P* < 0.0001, exact *P* values are shown within the figure legends for each figure. *P* > 0.05 was considered not significant. All data are presented as mean ± SEM unless otherwise noted.

### Reporting summary

Further information on research design is available in the Nature Portfolio Reporting Summary linked to this article.

## Supplementary information

Supplementary Information

Description of Additional Supplementary Files

Supplementary Movie 1

Reporting Summary

Transparent Peer Review file

## Source data

Source data

## Data Availability

The authors declare that the data supporting the findings of this study are available within the paper and its Supplementary Information file. Source Data file for Figs. 1b–d, h, 2b, d, f, i, k, 3a–c, e, f, h, 4a–f, i–l, 5a, b, d, f, 6a, b, d–g, i, l, 7b, d, f, h, j and Supplementary Figs. 1d, h, 2c, d, g, 3, 4a–f, 6a–e, 7b, d–f, h, 8a, c, e, g is provided with this paper. RNA sequencing data are available at Gene Expression Omnibus under the accession number GSE341178. Lipidomic data are available at MetaboLights under accession number MTBLS15181. Reagents and cell lines generated for this study are available directly from the authors upon request. Source data are provided with this paper.
