# Supplementary material for: Cholesterol maintains the degradative capacity of lysosomes during clearance and recycling of dysfunctional mitochondria
Source: Nat Commun. 2026 Sep 16;17:9647. doi: 10.1038/s41467-026-77423-1 (PMC13582898; doi:10.1038/s41467-026-77423-1)
Supplement: Supplementary file 1 [file 41467_2026_77423_MOESM1_ESM.pdf]

Supplementary Information

Cholesterol maintains the degradative capacity of lysosomes during clearance and recycling of dysfunctional mitochondria

Haoning Yang[1]**, Koji Matsuhisa[1, 5]**, Meng Wei[1], Wataru Nishi[1], Dylan Hong Zheng Koh[1], Aik Yong Sim[2], Isabelle Bonne[2,3], Yasunori Saheki[1,4]*.

[1]Lee Kong Chian School of Medicine, Nanyang Technological University, Singapore 308232, Singapore.

[2]Electron Microscopy Unit (EMU), Microscopy Cluster, Yong Loo Lin School of Medicine, National University of Singapore, Singapore 117549, Singapore.

[3]Department of Microbiology & Immunology, Yong Loo Lin School of Medicine, National University of Singapore, Singapore 117545, Singapore.

[4]Institute of Resource Development and Analysis, Kumamoto University, Kumamoto 860-0811, Japan.

[5]Present address: Department of Pharmacology and Therapeutic Innovation, Nagasaki University Graduate School of Biomedical Sciences, Nagasaki 852-8521, Japan.

*Address correspondence to:

yasunori.saheki@ntu.edu.sg (Y.S.); Telephone: +65 6592-3996; Fax: +65 6515-0417.

**These authors contributed equally: Haoning Yang, Koji Matsuhisa.

- **Supplementary Figures 1-8**
- **Supplementary Tables 1-3**

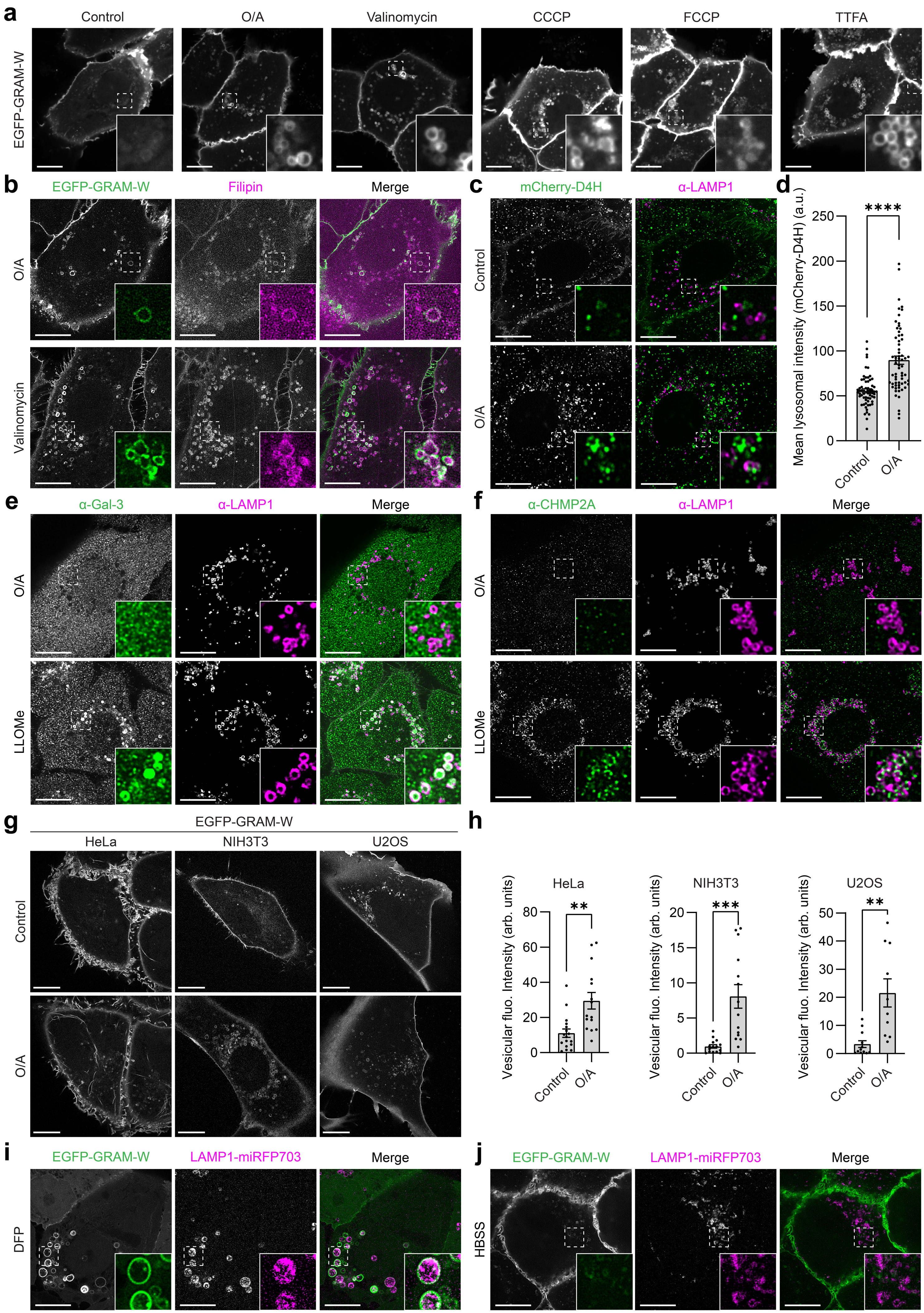

a
Control
O/A
Valinomycin
CCCP
FCCP
TTFA
EGFP-GRAM-W
b
EGFP-GRAM-W
Filipin
Merge
O/A
Valinomycin
c
mCherry-D4H
α-LAMP1
Control
O/A
d
Mean lysosomal intensity (mCherry-D4H) (a.u.)
****
250
200
150
100
50
0
Control
O/A
e
α-Gal-3
α-LAMP1
Merge
O/A
LLOMe
f
α-CHMP2A
α-LAMP1
Merge
O/A
LLOMe
g
EGFP-GRAM-W
HeLa
NIH3T3
U2OS
Control
O/A
h
HeLa
NIH3T3
U2OS
Vesicular fluo. Intensity (arb. units)
**
***
**
80
60
40
20
0
20
15
10
5
0
50
40
30
20
10
0
Control
O/A
i
EGFP-GRAM-W
LAMP1-miRFP703
Merge
DFP
j
EGFP-GRAM-W
LAMP1-miRFP703
Merge
HBSS

Supplementary Figure 1. Cholesterol accumulates in the membranes of degradative lysosomes when mitochondria are damaged. a. Confocal images of live N/TERT cells expressing EGFP-GRAM-W that were treated with ethanol (Control), 10 µM oligomycin and 4 µM antimycin A (O/A), 10 µM valinomycin, 10 µM carbonyl cyanide m-chlorophenyl hydrazone (CCCP), 10 µM carbonyl cyanide-p-trifluoromethoxyphenylhydrazone (FCCP), or 10 µM thenoyltrifluoroacetone (TTFA) for 2 hours. Insets show at higher magnification the regions indicated by white dashed boxes. **b.** SDC-SIM images of fixed N/TERT cells expressing EGFP-GRAM-W that were treated with O/A or 10 µM valinomycin for 2 hours. Cells were stained with filipin. **c.** SDC-SIM images of N/TERT cells that were treated with ethanol (Control) or O/A for 2 hours. Cells were fixed, cryo permeabilized, stained with purified mCherry-D4H protein, and immunolabeled against LAMP1. **d.** Quantification of lysosomal mCherry-D4H from (c) (mean ± SEM, n = 63 cells for Control and 64 cells for O/A; data are pooled from three independent experiments; Two-tailed unpaired t-test, ****$P < 0.0001$). **e-f.** SDC-SIM images of fixed N/TERT cells expressing EGFP-GRAM-W that were treated with O/A for 2 hours or 500 µM LLOMe for 1 hour. Cells were immunolabeled against galectin-3 (Gal-3) or CHMP2A, together with LAMP1. **g.** SDC-SIM images of live HeLa, NIH3T3, and U2OS cells expressing EGFP-GRAM-W that were treated with ethanol (Control) or O/A for 4 hours. **h.** Quantification of the signals of EGFP-GRAM-W on the vesicle-like structures, as shown in (g) (mean ± SEM, n = 17 and 15 cells for HeLa, n = 15 and 14 cells for NIH3T3, n = 12 and 10 cells for U2OS; representative data from one experiment are shown; Two-tailed unpaired t-test, **$P = 0.0011$ for HeLa, **$P = 0.001$ for U2OS, ***$P = 0.0002$ for NIH3T3). **i.** SDC-SIM images of live N/TERT cells stably expressing EGFP-GRAM-W and LAMP1-miRFP703 that were treated with 1 mM Deferiprone (DFP) for 24 hours. **j.** SDC-SIM images of live N/TERT cells stably expressing EGFP-GRAM-W and LAMP1-miRFP703 that were incubated in Hanks' Balanced Salt Solution (HBSS) for 24 hours. All scale bars, 10 µm.

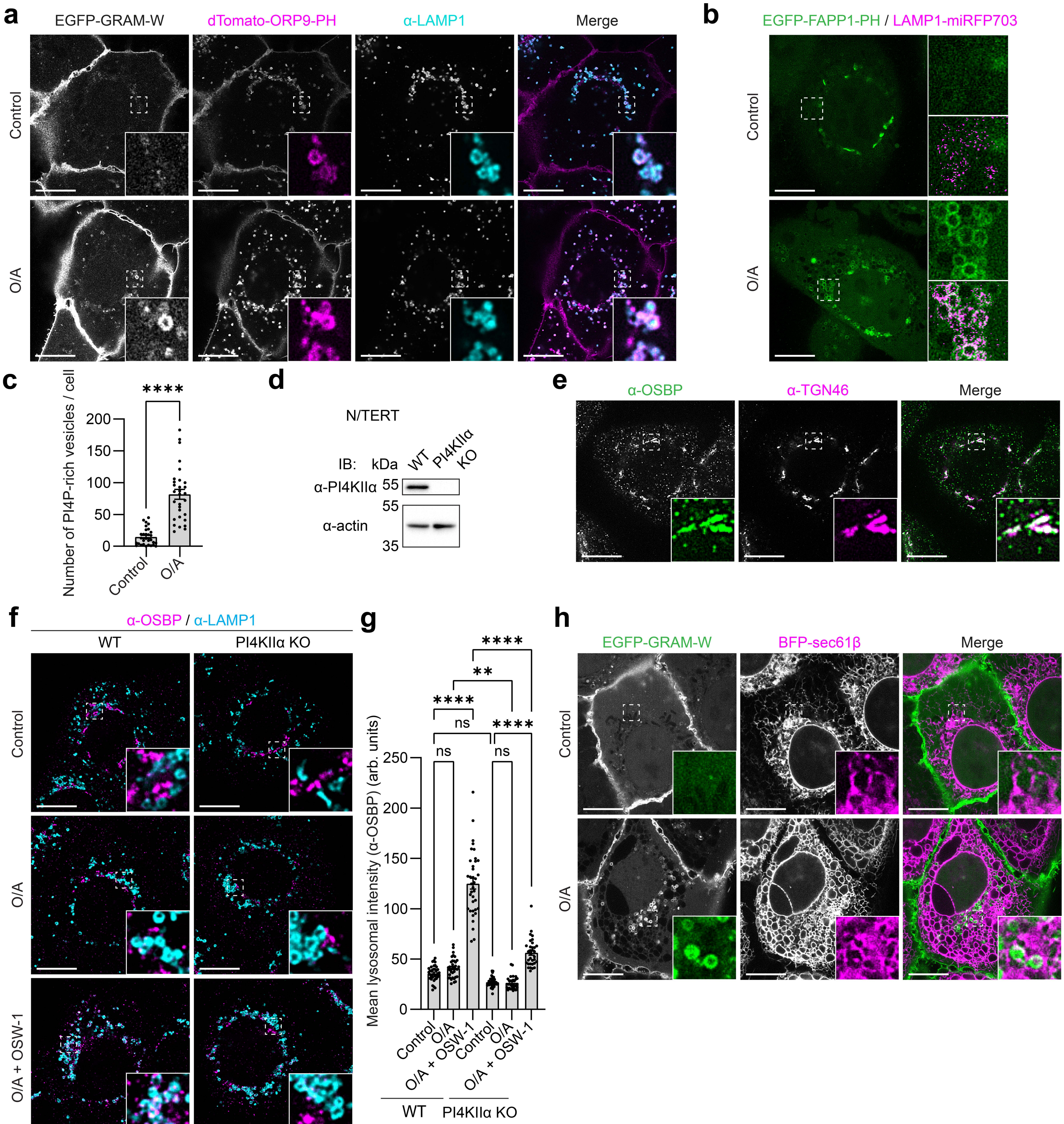

a
EGFP-GRAM-W
dTomato-ORP9-PH
α-LAMP1
Merge
Control
O/A
b
EGFP-FAPP1-PH / LAMP1-miRFP703
Control
O/A
c
Number of PI4P-rich vesicles / cell
****
200
150
100
50
0
Control
O/A
d
N/TERT
IB:
kDa
WT
PI4KIIα KO
α-PI4KIIα
55
55
α-actin
35
e
α-OSBP
α-TGN46
Merge
f
α-OSBP / α-LAMP1
WT
PI4KIIα KO
Control
O/A
O/A + OSW-1
g
Mean lysosomal intensity (α-OSBP) (arb. units)
****
**
****
ns
ns
ns
****
250
200
150
100
50
0
Control
O/A
O/A + OSW-1
Control
O/A
O/A + OSW-1
WT
PI4KIIα KO
h
EGFP-GRAM-W
BFP-sec61β
Merge
Control
O/A

Supplementary Figure 2. Cholesterol accumulates on degradative lysosomes through PI4P-driven, OSBP-mediated cholesterol transport. a. SDC-SIM images of fixed N/TERT cells expressing EGFP-GRAM-W and dTomato-ORP9-PH that were treated with ethanol (Control) or O/A for 2 hours. Cells were immunolabeled with antibodies against LAMP1. Insets show at higher magnification the regions indicated by white dashed boxes. Scale bars, 10 µm. **b.** SDC-SIM images of live N/TERT cells stably expressing EGFP-FAPP1-PH and LAMP1-miRFP703 that were treated with ethanol (Control) or O/A for 2 hours. Insets show at higher magnification the regions indicated by white dashed boxes. Scale bars, 10 µm. **c.** Quantification of the number of PI4P-rich vesicles (detected by EGFP-FAPP1-PH) as shown in (b) (mean ± SEM, n = 30 cells for each condition; data are pooled from three independent experiments; Two-tailed unpaired t-test, ****P < 0.0001). **d.** Lysates of N/TERT wild-type (WT) and PI4KIIα KO cells stably expressing EGFP-GRAM-W were processed for SDS-PAGE and immunoblotting (IB) with antibodies against PI4KIIα and actin. **e.** SDC-SIM images of fixed N/TERT cells expressing EGFP-GRAM-W. Cells were immunolabeled with antibodies against OSBP and TGN46. Insets show at higher magnification the regions indicated by white dashed boxes. Scale bars, 10 µm. **f.** SDC-SIM images of fixed wild-type (WT) and PI4KIIα KO N/TERT cells expressing EGFP-GRAM-W, that were treated with ethanol (Control), or O/A for 2 hours, or pre-treated with O/A for 1.5 hours, and then treated with 20 nM OSW-1 for 0.5 hours in the presence of O/A. Cells were immunolabeled with antibodies against OSBP and LAMP1. Insets show at higher magnification the regions indicated by white dashed boxes. Scale bars, 10 µm. **g.** Quantification of α-OSBP signals on lysosomal membranes (labeled with antibodies against LAMP1) as shown in (f) (mean ± SEM, n = 38, 36, 37, 36, 33, 33 cells for each condition; data are pooled from three independent experiments; Tukey's multiple comparisons test, ns, not significant, **P = 0.0017, ****P < 0.0001). **h.** SDC-SIM images of live N/TERT cells stably expressing EGFP-GRAM-W and BFP-Sec61β that were treated with ethanol (Control) or O/A for 2 hours. Insets show at higher magnification the regions indicated by white dashed boxes. Scale bars, 10 µm.

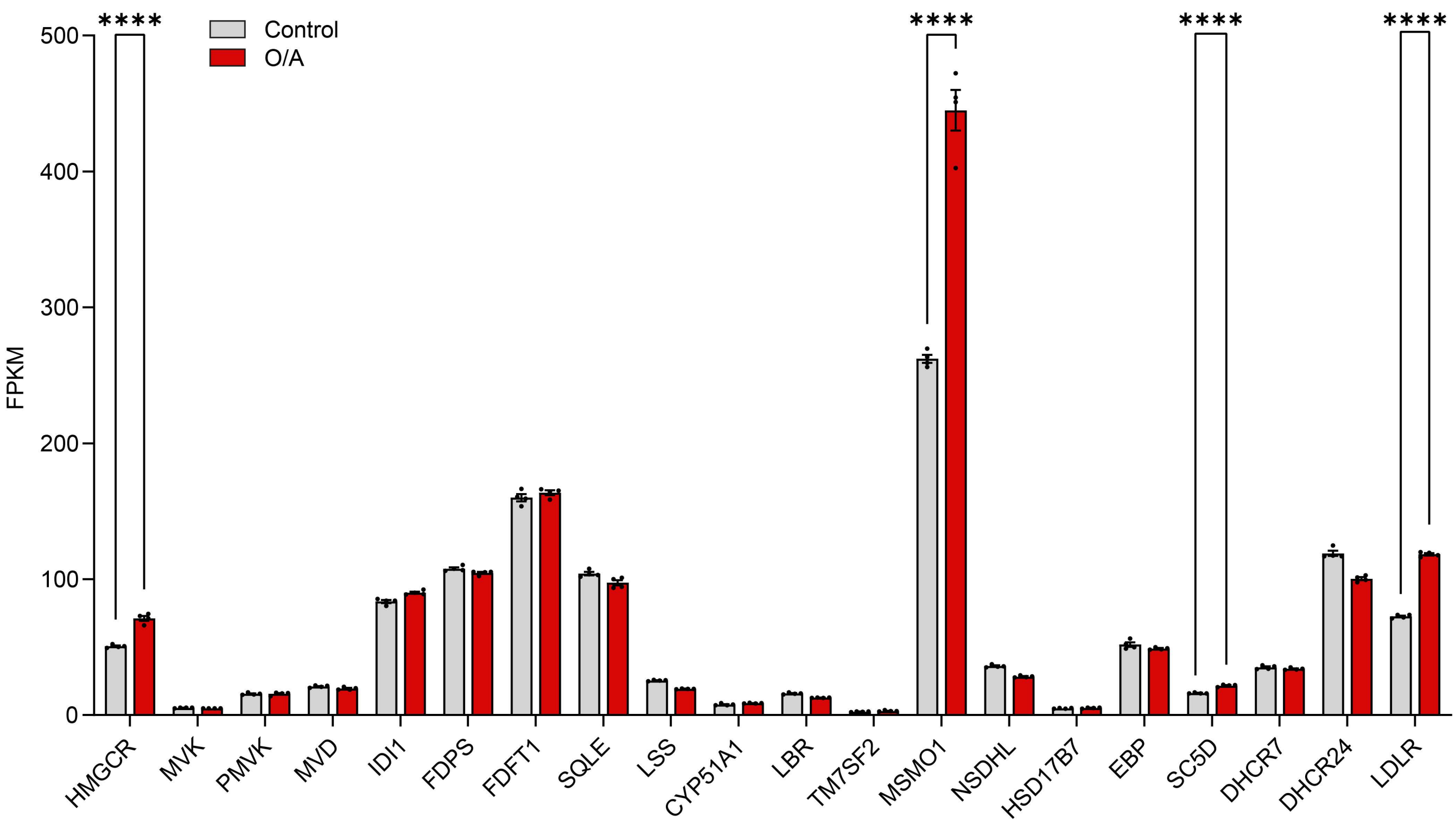

Supplementary Figure 3. Cholesterol accumulation on degradative lysosomal membranes is accompanied by SREBP-2 activation and enhanced cholesterol biosynthesis. Fragments per kilo bases per million reads (FPKM) values of genes involved in the mevalonate pathway and cholesterol uptake in N/TERT cells treated with ethanol (control) or O/A as shown in Figure 3d and 3e. Source data are provided as a Source Data file (mean ± SEM, n = 4 replicates, Two-tailed unpaired t-test, ****P < 0.0001).

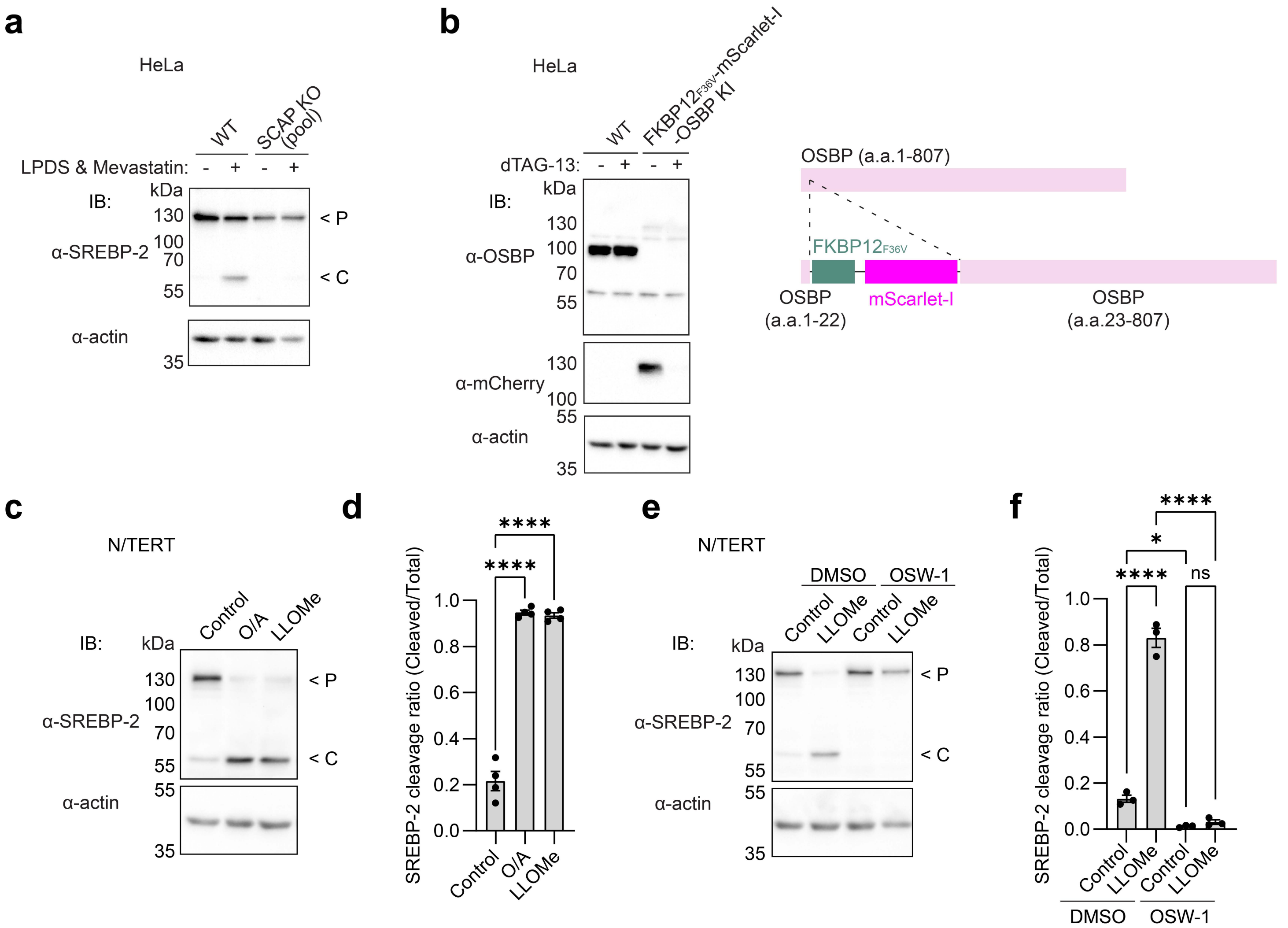

Supplementary Figure 4. Activation of SREBP-2 is driven by OSBP-mediated cholesterol transport from the ER to the lysosome. a. Lysates of wild-type (WT) and SCAP knockout (KO) HeLa cells incubated overnight in normal medium or cholesterol depletion medium (DMEM supplemented with LPDS and 50 µM mevastatin) were processed for SDS-PAGE and IB with antibodies against SREBP-2 and actin. Precursor (P) and cleaved (C) forms of SREBP-2 are indicated. **b.** Left: lysates of WT and FKBP12F36V-mScarlet-I-OSBP knock-in (KI) HeLa cells treated with DMSO or 10 µM dTAG-13 for 24 hours were processed for SDS-PAGE and IB with antibodies against OSBP, mCherry, and actin. Right: schematic gene map of FKBP12F36V-mScarlet-I-OSBP KI. FKBP12F36V and mScarlet-I are inserted between amino acid (a.a.) 22 and 23 of OSBP. **c.** Lysates of N/TERT cells treated with ethanol (Control) for 4 hours, O/A for 4 hours, or 500 µM LLOMe for 1 hour were processed for SDS-PAGE and IB with antibodies against SREBP-2 and actin. Precursor (P) and cleaved (C) forms of SREBP-2 are indicated. **d.** Quantification of the ratio of cleaved SREBP-2 to total SREBP-2 as shown in (c) (mean ± SEM, n = 4 lysates for each condition; Tukey's multiple comparisons test, ****P < 0.0001). **e.** Lysates of N/TERT cells pre-treated with DMSO or 20 nM OSW-1 for 3 hours, followed by treatment with DMSO or 500 µM LLOMe for 1 hour in the continued presence of the same drug were processed for SDS-PAGE and IB with antibodies against SREBP-2 and actin. Precursor (P) and cleaved (C) forms of SREBP-2 are indicated. **f.** Quantification of the ratio of cleaved SREBP-2 to total SREBP-2 as shown in (e) (mean ± SEM, n = 3 lysates for each condition; Tukey's multiple comparisons test, *P = 0.0255, ****P < 0.0001).

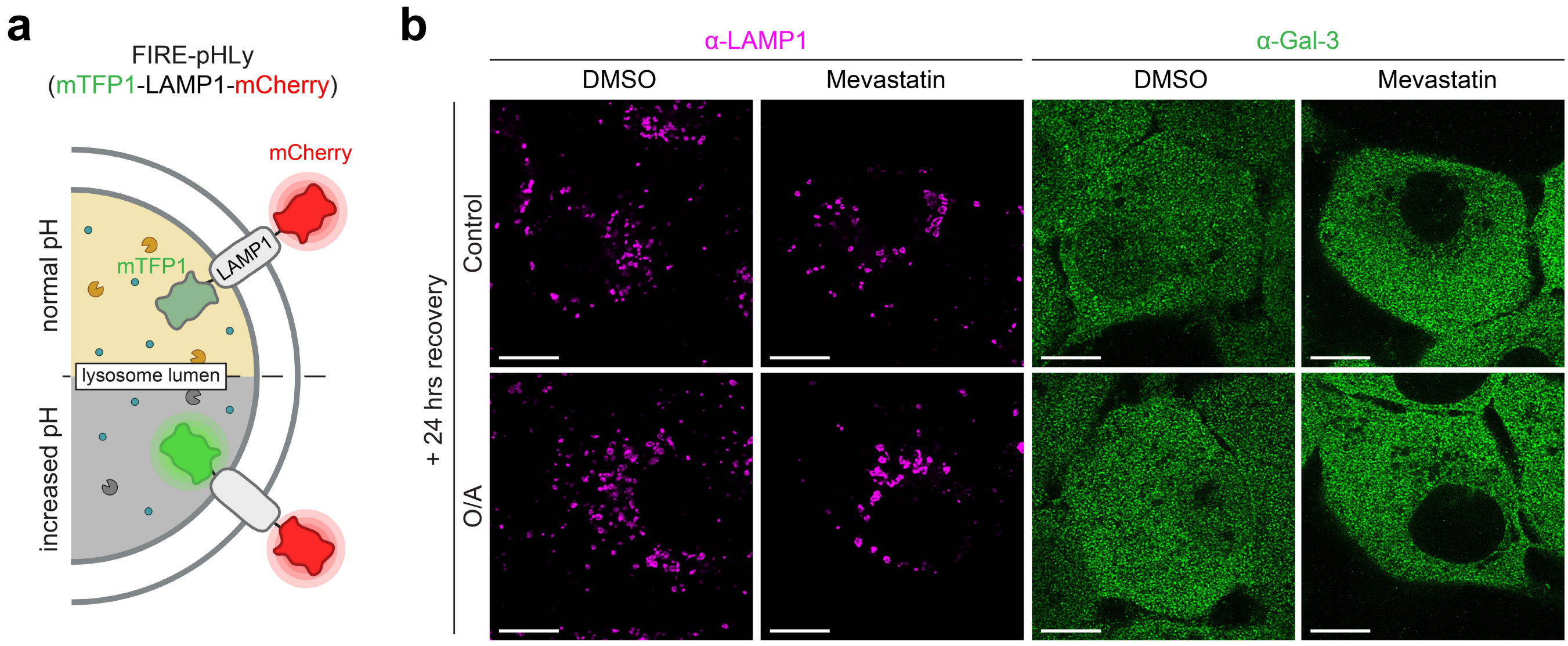

Supplementary Figure 5. Cholesterol contributes to the degradative capacity and membrane integrity of degradative lysosomes. a. Schematic representation of FIRE-pHLy (mTFP1-LAMP1-mCherry) used for the assessment of lysosomal pH. The pH-sensitive mTFP1 is located inside the lumen of lysosomes whereas mCherry is located in the cytosol. The fluorescence of mTFP1 is weak in the acidic environment (normal lysosomal pH) and increases as lysosomal pH increases, whereas mCherry fluorescence remains unaffected. Created in BioRender. Saheki, Y. (2026) https://BioRender.com/57ub7my. **b.** SDC-SIM images of fixed N/TERT cells that were pre-treated with DMSO or 5 μM mevastatin for 16 hours and then treated with ethanol (Control) or O/A for 2 hours. Cells were then washed with PBS and recovered in KSFM containing DMSO or 5 μM mevastatin for 24 hours. Cells were immunolabeled with antibodies against LAMP1 and galectin-3 (Gal-3). Scale bars, 10 μm.

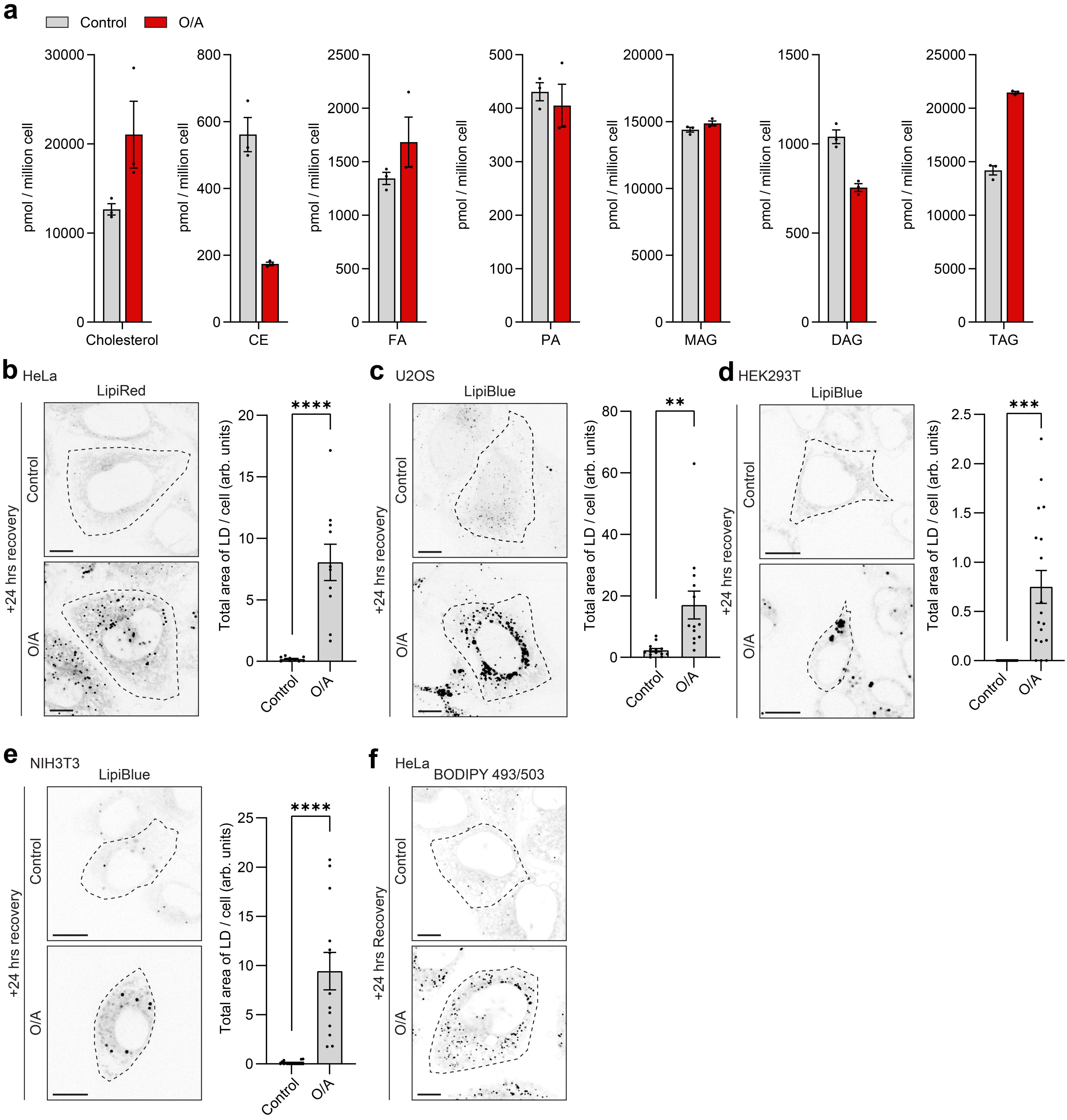

Supplementary Figure 6. Depolarization of mitochondria is followed by the formation of DGAT1-dependent lipid droplets. a. Amount of the indicated lipids per million cells in ethanol-treated (Control) and O/A-treated N/TERT cells as shown in Figure 6a, 6b, and 6d-6g (mean ± SEM, n = 3 biological replicates; data are pooled from three independent experiments). **b-e.** Left: confocal images of indicated live cells that were treated with ethanol (Control) or O/A for 2 hours, washed with PBS, and recovered in DMEM for 24 hours followed by staining using LipiRed or LipiBlue as indicated. Dotted lines indicate cell boundaries. Scale bars, 10 μm. Right: quantification of the total area of lipid droplet (LD) in each cell, as shown on the left panel [mean ± SEM, n = 10 cells for each condition for (b); n = 14 cells for Control and 13 cells for O/A for (c); n = 17 cells for Control and 18 cells for O/A for (d); n = 17 cells for Control and 13 cells for O/A for (e); representative data from one experiment are shown; Two-tailed unpaired t-test, **P = 0.0026, ***P = 0.0001, ****P < 0.0001]. **f.** Confocal images of live HeLa cells that were treated with ethanol (Control) or O/A for 2 hours, washed with PBS, and recovered in DMEM for 24 hours followed by staining using BODIPY 493/503. Dotted lines indicate cell boundary. Scale bars, 10 μm.

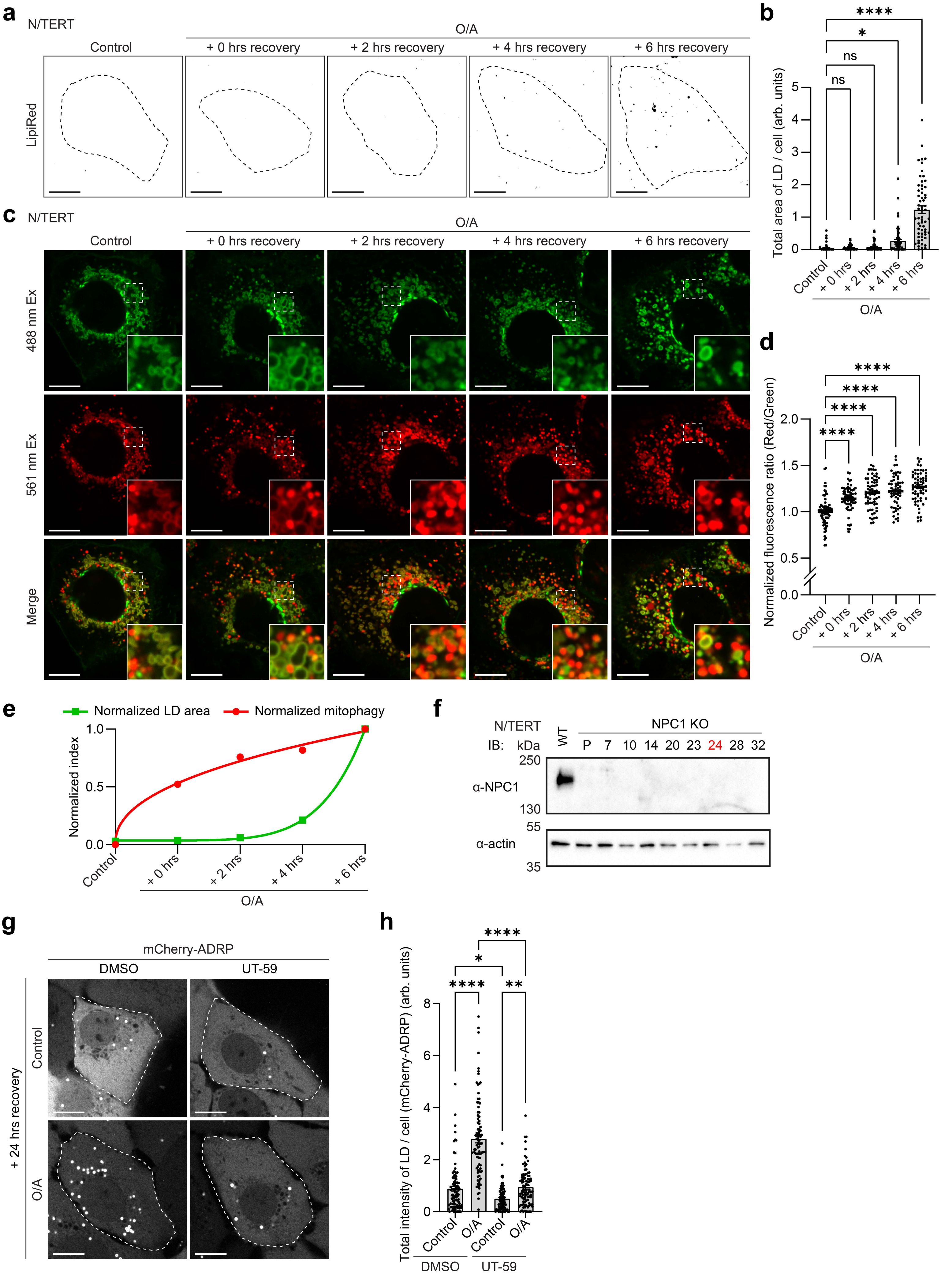
a
N/TERT
O/A
Control
+ 0 hrs recovery
+ 2 hrs recovery
+ 4 hrs recovery
+ 6 hrs recovery
LipiRed
b
Total area of LD / cell (arb. units)
****
*
ns
ns
Control
+ 0 hrs
+ 2 hrs
+ 4 hrs
+ 6 hrs
O/A
c
N/TERT
488 nm Ex
561 nm Ex
Merge
d
Normalized fluorescence ratio (Red/Green)
e
Normalized LD area
Normalized mitophagy
Normalized index
f
N/TERT
IB:
kDa
WT
NPC1 KO
P
7
10
14
20
23
24
28
32
α-NPC1
α-actin
250
130
55
35
g
mCherry-ADRP
DMSO
UT-59
Control
O/A
+ 24 hrs recovery
h
Total intensity of LD / cell (mCherry-ADRP) (arb. units)
****
*
**
DMSO
UT-59

Supplementary Figure 7. Degradative capacity of lysosomes is essential for lipid droplet formation during the clearance of depolarized mitochondria. a. Confocal images of live N/TERT cells that were treated with O/A for 2 hours, washed with PBS, and recovered in KSFM for the indicated hours. Control cells are not treated. Cells were stained with LipiRed. Dotted lines indicate cell boundaries. **b.** Quantification of lipid droplets (LDs) from (a) (mean ± SEM, n = 60 cells for each condition; data are pooled from three independent experiments; Dunnett's multiple comparisons test, ns denotes not significant, *P = 0.0248, ****P < 0.0001). **c.** Confocal images of live N/TERT cells stably expressing TOM20-EGFP-mScarlet that were treated with O/A for 2 hours, washed with PBS, and recovered in KSFM for the indicated hours. Control cells are not treated. **d.** Quantification of the ratio of total fluorescence intensity of mScarlet to that of EGFP from (c) (mean ± SEM, n = 71, 64, 66, 56, 64 cells for each condition; data are pooled from three independent experiments; Dunnett's multiple comparisons test, ****P < 0.0001). **e.** Non-linear regression analysis of normalized LD area and mitophagy index / fluorescence ratio (red/green) as analyzed in (b) and (d). **f.** Lysates of N/TERT wild-type (WT) and NPC1 knockout (KO) cells stably expressing EGFP-GRAM-W were processed for SDS-PAGE and IB with antibodies against NPC1 and actin. P, parental pool. Clone numbers are indicated. Clone 24, which is indicated in red, is used in this study. **g.** Confocal images of live N/TERT cells stably expressing mCherry-ADRP that were pre-treated with DMSO or 5 μM UT-59 for 24 hours and then treated with ethanol (Control) or O/A for 2 hours in the continued presence of the same drug. Cells were then washed with PBS and recovered in KSFM containing DMSO or 5 μM UT-59 for 24 hours. Dotted lines indicate the cell boundaries. **h.** Quantification of LD from (g) (mean ± SEM, n = 96, 92, 92, 92 cells for each condition; data are pooled from three independent experiments; Tukey's multiple comparisons test, *P = 0.0358, **P = 0.0099, ****P < 0.0001). All scale bars, 10 μm.

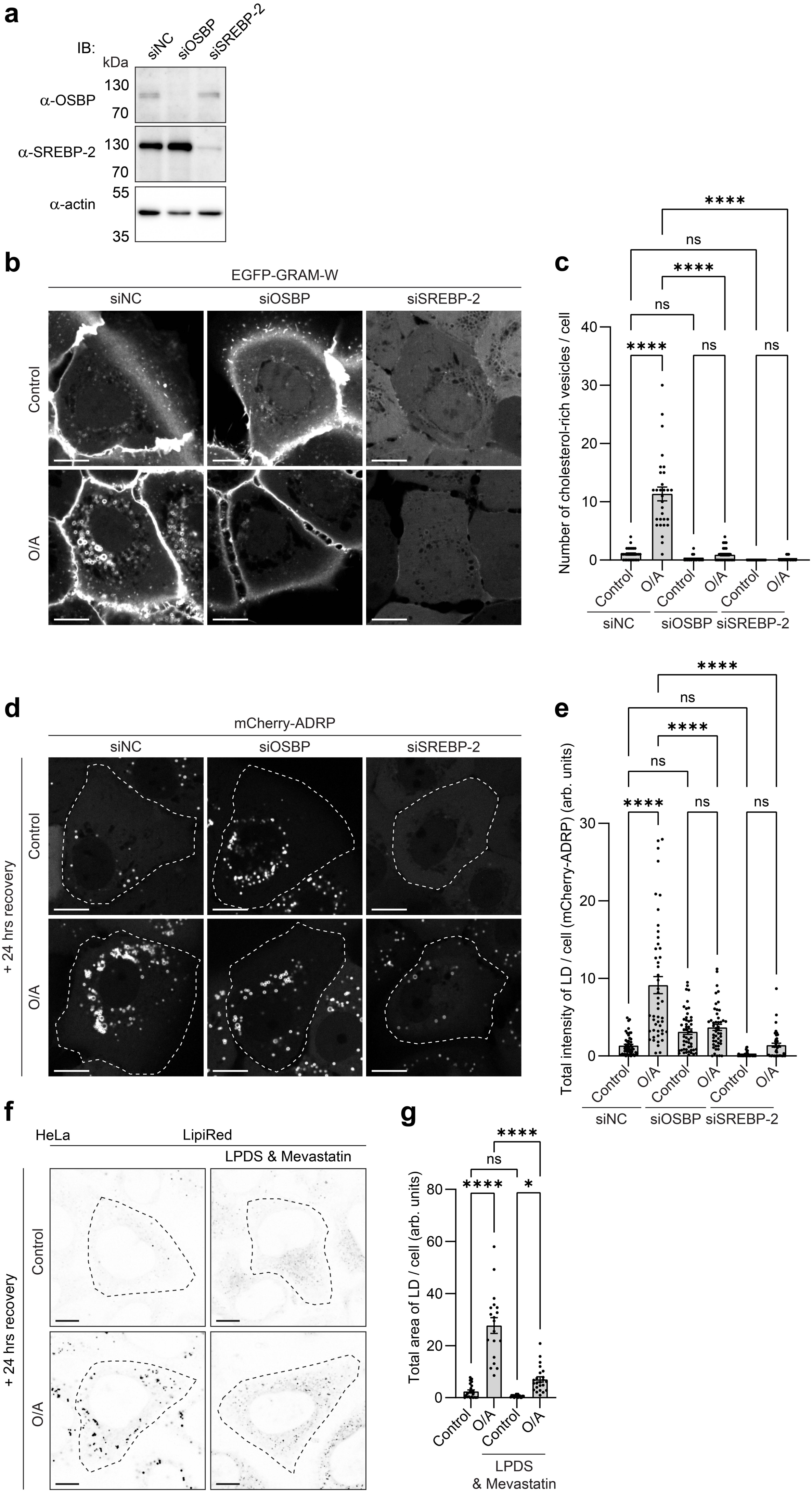

a
IB:
kDa
siNC
siOSBP
siSREBP-2
α-OSBP
α-SREBP-2
α-actin
130
70
130
70
55
35
b
EGFP-GRAM-W
siNC
siOSBP
siSREBP-2
Control
O/A
c
Number of cholesterol-rich vesicles / cell
40
30
20
10
0
****
ns
****
ns
ns
ns
****
ns
Control
O/A
Control
O/A
Control
O/A
siNC
siOSBP
siSREBP-2
d
mCherry-ADRP
siNC
siOSBP
siSREBP-2
+ 24 hrs recovery
Control
O/A
e
Total intensity of LD / cell (mCherry-ADRP) (arb. units)
30
20
10
0
****
ns
****
ns
ns
ns
****
ns
Control
O/A
Control
O/A
Control
O/A
siNC
siOSBP
siSREBP-2
f
HeLa
LipiRed
LPDS & Mevastatin
+ 24 hrs recovery
Control
O/A
g
Total area of LD / cell (arb. units)
80
60
40
20
0
****
ns
****
*
Control
O/A
Control
O/A
LPDS
& Mevastatin

Supplementary Figure 8. Cholesterol transport to degradative lysosomal membranes is essential for lipid droplet formation during mitophagy. a. Lysates of N/TERT cells stably expressing EGFP-GRAM-W and mCherry-ADRP treated with either non-targeting siRNA (siNC), siRNA targeting OSBP (siOSBP), or siRNA targeting SREBP-2 (siSREBP-2) for 72 hours were processed for SDS-PAGE and IB with antibodies against OSBP, SREBP-2, and actin. Note the efficient depletion of OSBP and SREBP-2 upon treatment with siOSBP and siSREBP-2, respectively. **b.** Confocal images of live N/TERT cells stably expressing EGFP-GRAM-W and mCherry-ADRP that were treated with siRNAs as indicated for 72 hours and then treated with ethanol (Control) or O/A for 2 hours. Scale bars, 10 μm. **c.** Quantification of the number of cholesterol-rich vesicles (detected by EGFP-GRAM-W) as shown in (b) (mean ± SEM, n = 30 cells for each condition; data are pooled from four independent experiments; Tukey's multiple comparisons test, ns, not significant, ****P<0.0001). **d.** Confocal images of live N/TERT cells stably expressing EGFP-GRAM-W and mCherry-ADRP that were treated with siRNAs as indicated for 72 hours and then treated with ethanol (Control) or O/A for 2 hours, washed with PBS and recovered in KSFM for 24 hours. Dotted lines indicate the cell boundaries. Scale bars, 10 μm. **e.** Quantification of the total intensity of LDs in each cell (labeled by mCherry-ADRP) as shown in (d) (mean ± SEM, n = 48, 49, 50, 48, 50, 48 cells for each condition; data are pooled from four independent experiments; Tukey's multiple comparisons test, ns, not significant, ****P < 0.0001). **f.** Confocal images of live HeLa cells that were pre-incubated in normal medium or cholesterol depletion medium (DMEM supplemented with LPDS and 50 μM mevastatin) for 16 hours and then treated with ethanol (Control) or O/A for 2 hours, washed with PBS, and recovered in respective medium for 24 hours. Cells were then stained with LipiRed. Dotted lines indicate cell boundaries. Scale bars, 10 μm. **g.** Quantification of the total area of LD in each cell, as shown in (f) (mean ± SEM, n = 21, 19, 18, 23 cells for each condition; representative data from one experiment are shown; Tukey's multiple comparisons test, ns, not significant, *P = 0.0233, ****P < 0.0001).

Supplementary Table 1. Association between TOM20-mScarlet and EGFP-GRAM-W signals on lysosomes.

Table showing the distribution of TOM20-positive and TOM20-negative lysosomes according to GRAM-W signal status. N/TERT cells expressing EGFP-GRAM-W and TOM20-mScarlet were treated with O/A for 2 hours. Cells were then immunolabeled with antibodies against LAMP1 as shown in Figure 1d. Values represent the number of lysosomes, with percentages relative to the total number of lysosomes analyzed. TOM20-positive lysosomes showed a higher frequency of GRAM-W positivity than TOM20-negative lysosomes (72.3% vs 64.9%; odds ratio = 1.41, 95% CI 1.16–1.72; χ^2 test, $P = 0.0007$) ($n = 1,831$ lysosomes from 18 cells across three independent experiments).

	GRAM-W positive	GRAM-W negative	Total
TOM20-positive	743 (40.58%)	285 (15.57%)	1028 (56.14%)
TOM20-negative	521 (28.45%)	282 (15.40%)	803 (43.86%)
Total	1264 (69.03%)	567 (30.97%)	1831 (100%)

Supplementary Table 2. List of reagents used in this study.

REAGENT or RESOURCE	SOURCE	IDENTIFIER
**Antibodies**		
Anti-Actin (1:1,000 dilution for WB)	EMD Millipore	RRID: AB_2223041
Anti-CHMP2A (1:500 dilution for IF)	Proteintech	RRID: AB_2079470
Anti-Galectin-3 (1:500 dilution for IF)	Proteintech	RRID: AB_2136768
Anti-LAMP1 (1:100 dilution for IF)	Developmental Studies Hybridoma Bank	RRID: AB_2296838
Anti-LC3B (1:500 dilution for IF)	Cell Signaling Technology	RRID: AB_2137707
Anti-mCherry (1:1,000 dilution for WB)	Proteintech	RRID: AB_2876881
Anti-MTCO2 (Anti-COXII) (1:300 dilution for WB)	Abcam	RRID: AB_10887758
Anti-NPC1 (1:1,000 dilution for WB)	Proteintech	RRID: AB_2152050
Anti-OSBP (1:1,000 dilution for WB)	Proteintech	RRID: AB_2158220
Anti-OSBP (1:100 dilution for IF)	Sigma-Aldrich/Merck	RRID: AB_2676401
Anti-Parkin (1:1,000 dilution for WB)	Cell Signaling Technology	RRID: AB_2159920
Anti-PI4KIIα (1:1,000 dilution for WB)	Santa Cruz Biotechnology	RRID: AB_3676719
Anti-SREBP-2 (1:500 dilution for WB)	Santa Cruz Biotechnology	RRID: AB_2194250
Anti-SREBP-2 (22D5) (1:1,000 dilution for WB) (used in Figure S8a only)	MilliporeSigma	Cat# MABS1988
Anti-TGN46 (1:500 dilution for IF)	Proteintech	RRID: AB_10597396
Goat anti-Mouse IgG (H+L) Secondary Antibody, Alexa Fluor 594 conjugate (1:500 dilution for IF)	Thermo Fisher Scientific	RRID: AB_2534091
Goat anti-Mouse IgG (H+L) Secondary Antibody, Alexa Fluor 647 conjugate (1:500 dilution for IF)	Thermo Fisher Scientific	RRID: AB_2535804
Goat Anti-Mouse IgG (H+L)-HRP Conjugate (1:5,000 dilution for WB)	Bio Rad	RRID: AB_11125547
Goat anti-Rabbit IgG (H+L) Secondary Ab, Alexa Fluor 647 conjugate	Thermo Fisher Scientific	RRID: AB_2535813
Goat Anti-Rabbit IgG (H+L)-HRP Conjugate (1:5,000 dilution for WB)	Bio Rad	RRID: AB_11125142
**Chemicals, Peptides, and Recombinant Proteins**		
2-Thenoyltrifluoroacetone (TTFA)	Sigma-Aldrich/Merck	T27006
Alt-R™ S.p. Cas9 Nuclease V3	IDT	1081058
Antimycin A	Sigma-Aldrich/Merck	A8674
Bafilomycin A1	Sigma-Aldrich/Merck	B1793
Benzonase® Nuclease	Santa Cruz Biotechnology	sc-202391
Blasticidin S HCl	Gibco	A1113903
BODIPY™ 493/503 (4,4-Difluoro-1,3,5,7,8-Pentamethyl-4-Bora-3a,4a-Diaza-s-Indacene)	Thermo Fisher Scientific	D3922
Carbonyl cyanide 4-(trifluoromethoxy)phenylhydrazone (FCCP)	Sigma-Aldrich/Merck	C2920
Carbonyl Cyanide m-Chlorophenylhydrazone (CCCP)	Sigma-Aldrich/Merck	C2759
Cholesterol	Sigma-Aldrich/Merck	C8667

Deferiprone	Sigma-Aldrich/Merck	Y0001976
dTAG-13	MedChemExpress	HY-114421
Fetal Bovine Serum	Gibco	10270-106
Filipin III ready made solution	Sigma-Aldrich/Merck	SAE0087
Hank's balanced salt solution (HBSS)	Gibco	14025-092
Hyclone DMEM/High Glucose w L-Glu & Phenol red w/o Na Pyruvate and HEPES	Hyclone	SH30022.01
Keratinocyte SFM (1X) Kit	Thermo Fisher Scientific	17005042
LipiBlue	Dojindo	LD01
LipiRed	Dojindo	LD03
Lipofectamine™ 2000 Reagent	Thermo Fisher Scientific	11668-019
Lipoprotein Deficient Serum from fetal calf	Sigma-Aldrich/Merck	S5394
L-leucyl-L-leucine methyl ester hydrobromide (LLOMe)	Sigma-Aldrich/Merck	L7393
LysoTracker™ Deep Red	Thermo Fisher Scientific	L12492
mCherry-D4H protein	Koh et. al. (2023) https://doi.org/10.1038/s41467-023-42498-7	
Methyl-β-cyclodextrin (MCD)	Sigma-Aldrich/Merck	C4555
Mevastatin	Santa Cruz Biotechonology	sc-200853A
Oligomycin	Sigma-Aldrich/Merck	O4876
Opti-MEM™ I Reduced Serum Medium	Thermo Fisher Scientific	31985070
OSW-1	Cayman Chemical	30310
Penicillin-Streptomycin (10,000 U/mL)	Thermo Fisher Scientific	15140122
Pierce™ 16% Formaldehyde (w/v), Methanol-free	Thermo Fisher Scientific	28908
Puromycin	Stemcell	73342
T863 (DGAT1 inhibitor)	Sigma-Aldrich/Merck	SML0539
UT-59	DC Chemicals	DC74218
Valinomycin	Sigma-Aldrich/Merck	V0627
**Critical Commercial Assays**		
BCA Protein Assay Kit	Thermo Fisher Scientific	23225
Pierce™ Dilution-Free™ Rapid Gold BCA Protein Assay	Thermo Fisher Scientific	A55860
Cholesterol/Cholesterol Ester-Glo™ Assay	Promega	J3190
NEBuilder HiFi DNA Assembly Cloning Kit	NEB	E5520S
P3 Primary Cell 4D-Nucleofector™ X Kit S	Lonza	V4XP-3032
Q5 Site-Directed Mutagenesis Kit	NEB	E0552S

Experimental Models: Cell Lines		
HEK293T	Gift from Nguan Soon Tan	
HEK293T stably expressing EGFP-GRAM-W	Koh et al. (2023) https://doi.org/10.1038/s41467-023-42498-7	
HeLa cells expressing endo-FKBP12F36V-mScarlet-I-OSBP	This paper	
HeLa M	Gift from Pietro De Camilli	
N/TERT cells stably expressing EGFP-GRAM-W	Koh et al. (2023) https://doi.org/10.1038/s41467-023-42498-7	
N/TERT cells stably expressing EGFP-GRAM-W and BFP-sec61b	This paper	
N/TERT cells stably expressing EGFP-GRAM-W and dTomato-ORP9-PH	This paper	
N/TERT cells stably expressing EGFP-GRAM-W together with dTomato-ORP9-PH and LAMP1-miRFP703	This paper	
N/TERT cells stably expressing EGFP-GRAM-W and LAMP1-miRFP703	This paper	
N/TERT cells stably expressing EGFP-GRAM-W and mCherry-ADRP	This paper	
N/TERT cells stably expressing EGFP-GRAM-W and TOM20-mScarlet	This paper	
N/TERT cells stably expressing BFP-Parkin	This paper	
N/TERT cells stably expressing EGFP-FAPP1-PH and LAMP1-miRFP703	This paper	
N/TERT cells stably expressing FIRE-pHLy	This paper	
N/TERT cells stably expressing TOM20-EGFP-mScarlet	This paper	
NIH3T3 stably expressing EGFP-GRAM-W	Koh et al. (2023) https://doi.org/10.1038/s41467-023-42498-7	
NPC1 KO N/TERT cells stably expressing EGFP-GRAM-W	This paper	
PI4KIIα KO N/TERT cells stably expressing EGFP-GRAM-W	This paper	
PI4KIIα KO N/TERT cells stably expressing EGFP-GRAM-W and dTomato-ORP9-PH	This paper	
SCAP KO HeLa cells	This paper	
U2OS	Gift from Wenting Zhao	
**Oligonucleotides**		
TCTAGAAGCTTCCGCCGCCGCCAAGTGCTGCAATGCGGAGTGCAGGTGGAAACCATC	This paper	HindIII-OSBPgRNArev-FKBP12_F
AGGATCCTCGAGATTGCAGCACTTGGCGGCGGCGGGCGCTACCTGAACCCTTGTACAGCTCGTCCATGCCGC	Naito et al (2023) https://doi.org/10.1038/s41467-023-41213-w	XhoI_pORANGE_mScarlet-OSBP_site_2_AS
GATCATATGGCTCCGGTGCCCGTCAG	This paper	NdeI-EF1alpha_F

AGTGCTAGCGTACGTCACGACACCTGAAATGGAAG	This paper	EF1alpha-NheI_R
AGTGCTAGCCACCATGGTGGGTCGGAAC	This paper	NheI-TOM20 F
CTGAGAATTCGTTGGGGTCACTTCGTCTTTTG	This paper	TOM20-EcoRI R
GACGTACGCTAGCGCTACCGGTCGCCACCATGGTGAGCAAG	This paper	HNP-180 7ex-NheI-AD297_F
GGTCGAGAATTCTCATCCTGAATCCAAACCTTGAAGCTGAAGAG	This paper	HNP-178 6ex-EcoRI-AD295_R
ATGGTGAGCAAGGGCGAGGCAG	This paper	HNP-169 IF_HN070_KM8_F
GTTGGGGTCACTTCGTCTTTTGC	This paper	HNP-170 IF_HN070_KM8_R
CGAAGTGACCCCAACGCTGGAATGGTGAGCAAGGGCGAGGAGCTG	This paper	HNP-171 IF_HN070_EGFP_F
GCCCTTGCTCACCATTCCAGCGGACTTGTACAGCTCGTCCATG	This paper	HNP-172 IF_HN070_EGFP_R
GATCCGCTAGCGGTCGCCACCATGAGCGAGCTGATTAAGGAGAACATGC	This paper	5ex-NheI-kozak-BFP-sec61b
CTGCAGAATTCCTACGAACGAGTGTACTTGCCCCAAATGTGCAAC	This paper	5ex-EcoRI-BFP-sec61b
GCATATGCTATTGCTAGCGCCACCATGAGCGAGCTGATTAAGG	This paper	12ex-NheI-kozak-BFP-parkin
GCATATGAATATGAATTCCTACACGTCGAACCAGTGGTCC	This paper	12ex-EcoRI-TAG-BFP-parkin
GAATTCTCGACCTCGA	This paper	KM3-IF-EcoRI-F
GTACGTCACGACACC	This paper	KM3-IF-NheI-R
CTGAGAATTCTCGACCTCGAGACAAATGGCAGTATTCATCCAC	This paper	KM3-Gib-EcoRI-F
GTGGCGCTAGCGTACGTCACGACACCTGAAATGGAAG	This paper	KM3-Gib-NheI-R
mC*mU*mC*rArCrGrCrArGrCrCrUrUrUrCrArGrUrCrArGrUrUrUrUrArGrArGrCrUrArGrArArArUrArGrCrArArGrUrUrArArArArUrArArGrGrCrUrArGrUrCrCrGrUrUrArUrCrArArCrUrUrGrArArArArArGrUrGrGrCrArCrCrGrArGrUrCrGrGrUrGrCmU*mU*mU*rU	This paper	SCAP gRNA1
mC*mA*mA*rGrGrGrCrUrCrCrArUrArGrUrGrGrUrUrArGrUrUrUrUrArGrArGrCrUrArGrArArArUrArGrCrArArGrUrUrArArArArUrArArGrGrCrUrArGrUrCrCrGrUrUrArUrCrArArCrUrUrGrArArArArArGrUrGrGrCrArCrCrGrArGrUrCrGrGrUrGrCmU*mU*mU*rU	This paper	SCAP gRNA2
mA*mC*mG*rCrCrUrGrUrArArUrGrCrCrArCrCrArArCrGrUrUrUrUrArGrArGrCrUrArGrArArArUrArGrCrArArGrUrUrArArArArUrArArGrGrCrUrArGrUrCrCrGrUrUrArUrCrArArCrUrUrGrArArArArArGrUrGrGrCrArCrCrGrArGrUrCrGrGrUrGrCmU*mU*mU*rU	This paper	NPC1 gRNA for exon5
mU*mA*mC*rUrGrCrGrUrGrUrUrCrGrUrCrArGrGrCrCrGrUrUrUrUrArGrArGrCrUrArGrArArArUrArGrCrArArGrUrUrArArArArUrArArGrGrCrUrArGrUrCrCrGrUrUrArUrCrArArCrUrUrGrArArArArArGrUrGrGrCrArCrCrGrArGrUrCrGrGrUrGrCmU*mU*mU*rU	This paper	NPC1 gRNA for exon8
mC*mC*mC*rCrGrGrGrCrCrCrGrArUrCrCrArArCrArGrGrUrUrUrUrArGrArGrCrUrArGrArArArUrArGrCrArArGrUrUrArArArArUrArArGrGrCrUrArGrUrCrCrGrUrUrArUrCrArArCrUrUrGrArArArArArGrUrGrGrCrArCrCrGrArGrUrCrGrGrUrGrCmU*mU*mU*rU	This paper	PI4K2A gRNA1
mC*mC*mA*rGrGrGrCrCrArGrArCrCrCrArArArCrCrGrGrUrUrUrUrArGrArGrCrUrArGrArArArUrArGrCrArArGrUrUrArArArArUrArArGrGrCrUrArGrUrCrCrGrUrUrArUrCrArArCrUrUrGrArArArArArGrUrGrGrCrArCrCrGrArGrUrCrGrGrUrGrCmU*mU*mU*rU	This paper	PI4K2A gRNA2

GGAGTGCAGGTGGAAACCATCTCCCCAGGAGACGGG<br>CGCACCTTCCCCAAGCGCGGCCAGACCTGCGTGGTGC<br>ACTACACCGGGATGCTTGAAGATGGAAAGAAAGTTGAT<br>TCCTCCCGGGACAGAAACAAGCCCTTTAAGTTTATGCT<br>AGGCAAGCAGGAGGTGATCCGAGGCTGGGAAGAAGG<br>GGTTGCCCAGATGAGTGTGGGTCAGAGAGCCAAACTG<br>ACTATATCTCCAGATTATGCCTATGGTGCCACTGGGCA<br>CCCAGGCATCATCCCACCACATGCCACTCTCGTCTTCG<br>ATGTGGAGCTTCTAAAACTGGAAGGCGGCGGCGTAGT<br>CGGGCACGTCGTAGGGGTAGGCTATCCGTATGATGTC<br>CCGGACTATGCAGGTTCTGGATCGGGTGTGAGCAAGG<br>GCGAGGCAGTGATCAAGGAGTTCATGCGGTTCAAGGT<br>GCACATGGAGGGCTCCATGAACGGCCACGAGTTCGAG<br>ATCGAGGGCGAGGGCGAGGGCCGCCCCTACGAGGGC<br>ACCCAGACCGCCAAGCTGAAGGTGACCAAGGGTGGCC<br>CCCTGCCCTTCTCCTGGGACATCCTGTCCCCTCAGTTC<br>ATGTACGGCTCCAGGGCCTTCATCAAGCACCCCGCCG<br>ACATCCCCGACTACTATAAGCAGTCCTTCCCCGAGGG<br>CTTCAAGTGGGAGCGCGTGATGAACTTCGAGGACGGC<br>GGCGCCGTGACCGTGACCCAGGACACCTCCCTGGAG<br>GACGGCACCCTGATCTACAAGGTGAAGCTCCGCGGCA<br>CCAACTTCCCTCCTGACGGCCCCGTAATGCAGAAGAA<br>GACAATGGGCTGGGAAGCGTCCACCGAGCGGTTGTAC<br>CCCGAGGACGGCGTGCTGAAGGGCGACATTAAGATGG<br>CCCTGCGCCTGAAGGACGGCGGCCGCTACCTGGCGG<br>ACTTCAAGACCACCTACAAGGCCAAGAAGCCCGTGCA<br>GATGCCCGGCGCCTACAACGTCGACCGCAAGTTGGAC<br>ATCACCTCCCACAACGAGGACTACACCGTGGTGGAAC<br>AGTACGAACGCTCCGAGGGCCGCCACTCCACCGGCG<br>GCATGGACGAGCTGTACAAGGGTTCAGGTAGC	This paper	FKBP_F36V-Scarlet-I

GCATATGCTATTGCTAGCGCCACCATGAGCGAGCTGAT<br>TAAGGAGAACATGCACATGAAGCTGTACATGGAGGGC<br>ACCGTGGACAACCATCACTTCAAGTGCACATCCGAGG<br>GCGAAGGCAAGCCCTACGAGGGCACCCAGACCATGA<br>GAATCAAGGTGGTCGAGGGCGGCCCTCTCCCCTTCGC<br>CTTCGACATCCTGGCTACTAGCTTCCTCTACGGCAGCA<br>AGACCTTCATCAACCACACCCAGGGCATCCCCGACTTC<br>TTCAAGCAGTCCTTCCCTGAGGGCTTCACATGGGAGA<br>GAGTCACCACATACGAAGACGGGGGCGTGCTGACCGC<br>TACCCAGGACACCAGCCTCCAGGACGGCTGCCTCATC<br>TACAACGTCAAGATCAGAGGGGTGAACTTCACATCCAA<br>CGGCCCTGTGATGCAGAAGAAAACACTCGGCTGGGAG<br>GCCTTCACCGAGACGCTGTACCCCGCTGACGGCGGCC<br>TGGAAGGCAGAAACGACATGGCCCTGAAGCTCGTGGG<br>CGGGAGCCATCTGATCGCAAACATCAAGACCACATATA<br>GATCCAAGAAACCCGCTAAGAACCTCAAGATGCCTGG<br>CGTCTACTATGTGGACTACAGACTGGAAAGAATCAAGG<br>AGGCCAACAACGAGACCTACGTCGAGCAGCACGAGGT<br>GGCAGTGGCCAGATACTGCGACCTCCCTAGCAAACTG<br>GGGCACAAGCTGAACTCAGGACTCAGATCTCGAGCTC<br>AAGCTTTAATAGTGTTTGTCAGGTTCAACTCCAGCCAT<br>GGTTTCCCAGTGGAGGTCGATTCTGACACCAGCATCTT<br>CCAGCTCAAGGAGGTGGTTGCTAAGCGACAGGGGGTT<br>CCGGCTGACCAGTTGCGTGTGATTTTCGCAGGGAAGG<br>AGCTGAGGAATGACTGGACTGTGCAGAATTGTGACCT<br>GGATCAGCAGAGCATTGTTCACATTGTGCAGAGACCGT<br>GGAGAAAAGGTCAAGAAATGAATGCAACTGGAGGCGA<br>CGACCCCAGAAACGCGGCGGGAGGCTGTGAGCGGGA<br>GCCCCAGAGCTTGACTCGGGTGGACCTCAGCAGCTCA<br>GTCCTCCCAGGAGACTCTGTGGGGCTGGCTGTCATTC<br>TGCACACTGACAGCAGGAAGGACTCACCACCAGCTGG<br>AAGTCCAGCAGGTAGATCAATCTACAACAGCTTTTATG<br>TGTATTGCAAAGGCCCCTGTCAAAGAGTGCAGCCGGG<br>AAAACTCAGGGTACAGTGCAGCACCTGCAGGCAGGCA<br>ACGCTCACCTTGACCCAGGGTCCATCTTGCTGGGATG<br>ATGTTTTAATTCCAAACCGGATGAGTGGTGAATGCCAA<br>TCCCCACACTGCCCTGGGACTAGTGCAGAATTTTTCTT<br>TAAATGTGGAGCACACCCCACCTCTGACAAGGAAACAT<br>CAGTAGCTTTGCACCTGATCGCAACAAATAGTCGGAAC<br>ATCACTTGCATTACGTGCACAGACGTCAGGAGCCCCG<br>TCCTGGTTTTCCAGTGCAACTCCCGCCACGTGATTTGC<br>TTAGACTGTTTCCACTTATACTGTGTGACAAGACTCAAT<br>GATCGGCAGTTTGTCCACGACCCTCAGCTTGGCTACTC<br>CCTGCCTTGTGTGGCTGGCTGTCCCAACTCCTTGATTA<br>AAGAGCTCCATCACTTCAGGATTCTGGGAGAAGAGCA<br>GTACAACCGGTACCAGCAGTATGGTGCAGAGGAGTGT<br>GTCCTGCAGATGGGGGGCGTGTTATGCCCCCGCCCTG<br>GCTGTGGAGCGGGGCTGCTGCCGGAGCCTGACCAGA<br>GGAAAGTCACCTGCGAAGGGGGCAATGGCCTGGGCT<br>GTGGGTTTGCCTTCTGCCGGGAATGTAAAGAAGCGTA<br>CCATGAAGGGGAGTGCAGTGCCGTATTTGAAGCCTCA<br>GGAACAACTACTCAGGCCTACAGAGTCGATGAAAGAG<br>CCGCCGAGCAGGCTCGTTGGGAAGCAGCCTCCAAAGA<br>AACCATCAAGAAAACCACCAAGCCCTGTCCCCGCTGC<br>CATGTACCAGTGGAAAAAAATGGAGGCTGCATGCACAT<br>GAAGTGTCCGCAGCCCCAGTGCAGGCTCGAGTGGTGC<br>TGGAACTGTGGCTGCGAGTGGAACCGCGTCTGCATGG<br>GGGACCACTGGTTCGACGTGTAGGAATTCATATTCATA<br>TGC	This paper	BFP-Parkin_gblock

CGTGACGTACGCTAGCGCCACCATGGTGAGCAAGGGC<br>GAGGAGCTGTTCACCGGGGTGGTGCCCATCCTGGTCG<br>AGCTGGACGGCGACGTAAACGGCCACAAGTTCAGCGT<br>GTCCGGCGAGGGCGAGGGCGATGCCACCTACGGCAA<br>GCTGACCCTGAAGTTCATCTGCACCACCGGCAAGCTG<br>CCCGTGCCCTGGCCCACCCTCGTGACCACCCTGACCT<br>ACGGCGTGCAGTGCTTCAGCCGCTACCCCGACCACAT<br>GAAGCAGCACGACTTCTTCAAGTCCGCCATGCCCGAA<br>GGCTACGTCCAGGAGCGCACCATCTTCTTCAAGGACG<br>ACGGCAACTACAAGACCCGCGCCGAGGTGAAGTTCGA<br>GGGCGACACCCTGGTGAACCGCATCGAGCTGAAGGG<br>CATCGACTTCAAGGAGGACGGCAACATCCTGGGGCAC<br>AAGCTGGAGTACAACTACAACAGCCACAACGTCTATAT<br>CATGGCCGACAAGCAGAAGAACGGCATCAAGGTGAAC<br>TTCAAGATCCGCCACAACATCGAGGACGGCAGCGTGC<br>AGCTCGCCGACCACTACCAGCAGAACACCCCCATCGG<br>CGACGGCCCCGTGCTGCTGCCCGACAACCACTACCTG<br>AGCACCCAGTCCGCCCTGAGCAAAGACCCCAACGAGA<br>AGCGCGATCACATGGTCCTGCTGGAGTTCGTGACCGC<br>CGCCGGGATCACTCTCGGCATGGACGAGCTGTACAAG<br>GGCGGTGGCGGTTCTATGGAGGGGGTGTTGTACAAGT<br>GGACCAACTATCTCACAGGCTGGCAGCCTCGTTGGTTT<br>GTTTTAGATAATGGAATCTTATCCTACTATGATTCACAA<br>GATGATGTTTGCAAAGGGAGCAAAGGAAGCATAAAGAT<br>GGCAGTTTGTGAAATTAAAGTTCATTCAGCAGACAACA<br>CAAGAATGGAATTAATCATTCCTGGAGAGCAGCATTTC<br>TACATGAAGGCAGTGAATGCAGCTGAAAGACAGAGGT<br>GGCTGGTCGCTCTGGGGAGCTCCAAAGCATGTTTGAC<br>TGATACAAGGACTAAGTAGGAATTCTCGACCTCGA	This paper	EGFP-FAPP1-PH_gblock
hs.Ri.OSBP.13.1-SEQ1:<br>rGrUrGrCrArArUrGrArCrUrUrGrArUrArGrCrUrArArGrCAT<br>hs.Ri.OSBP.13.1-SEQ2:<br>rArUrGrCrUrUrArGrCrUrArUrCrArArGrUrCrArUrUrGrCrArCrGrU	This paper	hs.Ri.OSBP.13.1
hs.Ri.OSBP.13.2-SEQ1:<br>rGrGrUrGrGrArArUrArArArGrUrCrArCrArUrGrArArArACA<br>hs.Ri.OSBP.13.2-SEQ2:<br>rUrGrUrUrUrUrCrArUrGrUrGrArCrUrUrUrArUrUrCrCrArCrCrUrG	This paper	hs.Ri.OSBP.13.2
hs.Ri.OSBP.13.3-SEQ1:<br>rGrArArArUrUrGrArUrArUrUrGrUrGrArArUrCrArCrArAGA<br>hs.Ri.OSBP.13.3-SEQ2:<br>rUrCrUrUrGrUrGrArUrUrCrArCrArArUrArUrCrArArUrUrUrCrGrC	This paper	hs.Ri.OSBP.13.3
hs.Ri.SREBF2.13.1-SEQ1:<br>rGrGrCrCrArUrUrGrArUrUrArCrArUrCrArArArUrArCrUTG<br>hs.Ri.SREBF2.13.1-SEQ2:<br>rCrArArGrUrArUrUrUrGrArUrGrUrArArUrCrArArUrGrGrCrCrUrU	This paper	hs.Ri.SREBF2.13.1
hs.Ri.SREBF2.13.2-SEQ1:<br>rArUrCrGrArGrGrArCrUrUrUrArArUrCrArGrArArUrGrUCC<br>hs.Ri.SREBF2.13.2-SEQ2:<br>rGrGrArCrArUrUrCrUrGrArUrUrArArArGrUrCrCrUrCrGrArUrCrU	This paper	hs.Ri.SREBF2.13.2
hs.Ri.SREBF2.13.3-SEQ1:<br>rArUrUrGrGrUrCrUrGrUrUrGrGrGrGrUrArUrUrArArArUGA<br>hs.Ri.SREBF2.13.3-SEQ2:<br>rUrCrArUrUrUrArArUrArCrCrCrCrArArCrArGrArCrCrArArUrUrG	This paper	hs.Ri.SREBF2.13.3
**Recombinant DNA**		

BFP-sec61 beta	Addgene	49154
EGFP-GRAM1b (G187W) (EGFP-GRAM-W)	Ercan et al. (2021) https://doi.org/10.15252/embj.2020106524	D60
mCherry-C1	Pietro De Camilli Lab	YS300
pCMV-dTomato-ORP9-PH	Addgene	214268
pEGFP-C1-ADRP	Addgene	87161
pLJM1_EF1alpha_BFP-Sec61b_Blast	This paper	HN080
pLJM1_EF1alpha_dTomato-ORP9-PH_BlastR	This paper	HN075
pLJM1_EF1alpha_EGFP_BlastR	This paper	KM3
pLJM1_EF1alpha_EGFP-FAPP1-PH_Blast	This paper	HN083
pLJM1_EF1alpha_LAMP1-miRFP703_BlastR	This paper	KM4
pLJM1_EF1alpha_TOM20-EGFP-mScarlet_In-Fusion	This paper	HN070
pLJM1_EF1alpha_TOM20-miRFP703_BlastR	This paper	KM6
pLJM1_EF1alpha_TOM20-mScarlet_BlastR	This paper	KM8
pLJM1_EF1alpha_BFP-Parkin	This paper	HN082
pLJM1-EF1alpha_mCherry-ADRP_BlastR	This paper	PCT4
pLJM1-EGFP	Addgene	19319
pLJM1-EGFP-BlastR	This paper	MY5
pLJM1-EGFP-GRAM1b (G187W) (pLJM1-EGFP-GRAM-W)	Koh et al. (2023) https://doi.org/10.1038/s41467-023-42498-7	D150
pLJM1-FIRE-pHLy	Addgene	170775
pmCherry-ADRP	This paper	KM26
pMD2.G	Addgene	12259
pMDLg/pRRE	Ch'ng Toh Hean Lab	D45
pmScarlet_C1	Addgene	85042
pORANGE_FKBP12_F36V-mScarlet-I-OSBP_site_2	This paper	KM37
pORANGE_intermediate_OSBP_2	Naito et al. (2023) https://doi.org/10.1038/s41467-023-41213-w	TN353
pRSV-Rev	Ch'ng Toh Hean Lab	D46
TOM20-mCherry-GAI(1-92)	Addgene	37316
**Software**		
Prism	Graph Pad	
ImageJ Fiji	Schneider et al (2012)	https://imagej.nih.gov/ij/
Imaris 9.1	Oxford Instruments	

Supplementary Table 3. List of internal standards used for lipidomic analysis.

Internal Standards	Name	IS con (μmol/L)
CE (16:0)-d7 (IS)	CE	20.582
CE (16:1)-d7 (IS)		20.648
CE (18:1)-d7 (IS)		86.674
CE (18:2)-d7 (IS)		218.114
CE (20:3)-d7 (IS)		22.006
CE (20:4)-d7 (IS)		26.485
CE (20:5)-d7		26.564
CE (22:6)-d7		31.267
Cer-NS (d18:1/16:0)-d9	Cer-NS	0.366
DG (16:0/16:0)-d9	DG	0.672
DG (16:0/18:0)-d9		0.801
DG (16:0/18:1)-d9		0.964
DG (16:0/18:2)-d9		0.806
DG (16:0/18:3)-d9		0.218
DG (16:0/20:4)-d9		0.233
DG (16:0/20:5)-d9		0.226
DG (16:0/22:6)-d9		2.389
Cer-NDS (d18:0/16:0)-d9	Cer-NDS	0.146
HexCer-NS (d18:1/16:0)-d9	HexCer-NS	0.423
Hex_2Cer (d18:1/16:0)-d9	Hex_2Cer	0.344
LPC (16:0)-d9	LPC	19.814
LPE (18:0)-d5	LPE	1.027
PC (16:0/16:1)-d9	PC	7.182
PC (16:0/18:1)-d9		30.387
PC (16:0/18:2)-d9		30.768
PC (16:0/18:3)-d9		7.864
PC (16:0/20:3)-d9		8.461
PC (16:0/20:4)-d9		32.467
PC (16:0/20:5)-d9		8.211
PC (16:0/22:4)-d9		8.474
PC (16:0/22:5)-d9		8.778
PC (16:0/22:6)-d9		16.465
PE (18:0/18:1)-d5	PE	0.673
PE (18:0/18:2)-d5		0.675
PE (18:0/18:3)-d5		0.142
PE (18:0/20:3)-d5		0.176
PE (18:0/20:4)-d5		0.654

PE (18:0/20:5)-d5		0.144
PE (18:0/22:5)-d5		0.152
PE (18:0/22:6)-d5		0.634
SM (d18:1/16:0)-d7	SM	4.721
SM (d18:1/18:1)-d7		4.554
SM (d18:1/24:0)-d7		4.077
SM (d18:1/24:1)-d7		3.646
TG (50:1)-FA16:0-d9	TG	7.644
TG (52:1)-FA18:0-d9		7.967
TG (52:2)-FA18:1-d9		7.986
TG (52:3)-FA18:2-d9		8.004
TG (52:4)-FA18:3-d9		2.292
TG (54:4)-FA20:3-d9		2.220
TG (54:5)-FA20:4-d9		2.225
TG (56:7)-FA22:6-d9		2.058
MG (17:1)	MG (17:1)	74.450
PG (15:0/15:0)	Diluted PG (15:0/15:0)	0.070
PI (17:0/20:4)	PI (17:0/20:4)	1.093
PI (8:0/8:0)	PI (8:0/8:0)	0.828
PI(17:0/14:1)	PI(17:0/14:1)	1.259
PA (17:0/17:0)	Diluted PA (17:0/17:0)	0.908
PS (17:0/17:0)	Diluted PS (17:0/17:0)	0.032
Sphingosine-1-phosphate (d17:1)	Diluted Sphingosine-1-phosphate (d17:1)	0.268
Sphingosine (d17:1)	Diluted Sphingosine (d17:1)	0.120
LPI (17:1)	Diluted LPI (17:1)	0.203
LPS (17:1)	Diluted LPS (17:1)	0.096
LPA (17:1)	LPA (17:1)	4.300
GM_3 (d18:1/18:0)-d5	GM3 (d18:1/18:0)-d5	0.831
LPG (17:1)	Diluted LPG (17:1)	0.482
ACar (16:0)-d3	Diluted ACar (16:0)-d3	0.241
Cholesterol-d7	Cholesterol-d7	365.764
FFA (16:0)-d9	FFA (16:0)-d9	18.834
