# Supplementary material for: Cholesterol maintains the degradative capacity of lysosomes during clearance and recycling of dysfunctional mitochondria
Source: Nat Commun. 2026 Sep 16;17:9647. doi: 10.1038/s41467-026-77423-1 (PMC13582898; doi:10.1038/s41467-026-77423-1)
Supplement: Supplementary file 2 [file 41467_2026_77423_MOESM2_ESM.pdf]

Description of Additional Supplementary Files

Supplementary Movie 1. Progressive accumulation of cholesterol on degradative lysosomes containing depolarized mitochondria.

A live N/TERT cell stably expressing EGFP-GRAM W (left) and TOM20-mScarlet (middle) was imaged using SDC-SIM. Merged images are shown on the right. Imaging started 30 minutes after O/A treatment, with images acquired every 2 minutes. The region around the plasma membrane is shown. Image size, 3.3 μm x 9.9 μm.
