# Supplementary material for: Cholesterol maintains the degradative capacity of lysosomes during clearance and recycling of dysfunctional mitochondria
Source: Nat Commun. 2026 Sep 16;17:9647. doi: 10.1038/s41467-026-77423-1 (PMC13582898; doi:10.1038/s41467-026-77423-1)
Supplement: Supplementary file 3 [file 41467_2026_77423_MOESM4_ESM.pdf]

nature portfolio

Corresponding author(s): Yasunori Saheki

Last updated by author(s): Aug 2, 2026

Reporting Summary

Nature Portfolio wishes to improve the reproducibility of the work that we publish. This form provides structure for consistency and transparency in reporting. For further information on Nature Portfolio policies, see our Editorial Policies and the Editorial Policy Checklist.

Statistics

For all statistical analyses, confirm that the following items are present in the figure legend, table legend, main text, or Methods section.

n/a	Confirmed	
☐	☒	The exact sample size (*n*) for each experimental group/condition, given as a discrete number and unit of measurement
☐	☒	A statement on whether measurements were taken from distinct samples or whether the same sample was measured repeatedly
☐	☒	The statistical test(s) used AND whether they are one- or two-sided<br>*Only common tests should be described solely by name; describe more complex techniques in the Methods section.*
☒	☐	A description of all covariates tested
☐	☒	A description of any assumptions or corrections, such as tests of normality and adjustment for multiple comparisons
☐	☒	A full description of the statistical parameters including central tendency (e.g. means) or other basic estimates (e.g. regression coefficient) AND variation (e.g. standard deviation) or associated estimates of uncertainty (e.g. confidence intervals)
☐	☒	For null hypothesis testing, the test statistic (e.g. *F*, *t*, *r*) with confidence intervals, effect sizes, degrees of freedom and *P* value noted<br>*Give P values as exact values whenever suitable.*
☒	☐	For Bayesian analysis, information on the choice of priors and Markov chain Monte Carlo settings
☒	☐	For hierarchical and complex designs, identification of the appropriate level for tests and full reporting of outcomes
☒	☐	Estimates of effect sizes (e.g. Cohen's *d*, Pearson's *r*), indicating how they were calculated

Our web collection on statistics for biologists contains articles on many of the points above.

Software and code

Policy information about availability of computer code

Data collection	MetaMorph (SDC microscope) was used for the image acquisition. Gen5 3.09 was used for collecting data for Cholesterol Glo assay. Image Lab was used for collecting data for immunoblotting.
Data analysis	Fiji (ImageJ) software and Imaris was used for image processing, Prism 11 (Graphpad) was used for statistical analysis.

For manuscripts utilizing custom algorithms or software that are central to the research but not yet described in published literature, software must be made available to editors and reviewers. We strongly encourage code deposition in a community repository (e.g. GitHub). See the Nature Portfolio guidelines for submitting code & software for further information.

Data

Policy information about availability of data

All manuscripts must include a data availability statement. This statement should provide the following information, where applicable:

- Accession codes, unique identifiers, or web links for publicly available datasets
- A description of any restrictions on data availability
- For clinical datasets or third party data, please ensure that the statement adheres to our policy

The authors declare that the data supporting the findings of this study are available within the paper and its supplementary information file. Source Data file for Figures 1b-1d, 1h, 2b, 2d, 2f, 2i, 2k, 3a-3c, 3e, 3f, 3h, 4a-4f, 4i-4l, 5a, 5b, 5d, 5f, 6a, 6b, 6d-6g, 6i, 6l, 7b, 7d, 7f, 7h, and 7j, and Supplementary Figures 1d, 1h, 2c, 2d, 2g, 3, 4a-4f, 6a-6e, 7b, 7d-7f, 7h, 8a, 8c, 8e, and 8g is provided with this paper. RNA sequencing data are available at Gene Expression Omnibus under the accession

number GSE341178 [https://www.ncbi.nlm.nih.gov/geo/query/acc.cgi?acc=GSE341178]. Lipidomic data are available at MetaboLights under accession number MTBLS15181 [https://www.ebi.ac.uk/metabolights/MTBLS15181]. Reagents and cell lines generated for this study are available directly from the authors upon request.

Research involving human participants, their data, or biological material

Policy information about studies with human participants or human data. See also policy information about sex, gender (identity/presentation), and sexual orientation and race, ethnicity and racism.

Reporting on sex and gender	Not applicable.
Reporting on race, ethnicity, or other socially relevant groupings	Not applicable.
Population characteristics	Not applicable.
Recruitment	Not applicable.
Ethics oversight	Not applicable.

Note that full information on the approval of the study protocol must also be provided in the manuscript.

Field-specific reporting

Please select the one below that is the best fit for your research. If you are not sure, read the appropriate sections before making your selection.

☒ Life sciences ☐ Behavioural & social sciences ☐ Ecological, evolutionary & environmental sciences

For a reference copy of the document with all sections, see nature.com/documents/nr-reporting-summary-flat.pdf

Life sciences study design

All studies must disclose on these points even when the disclosure is negative.

Sample size	No statistical method was used to predetermine sample size. For imaging experiments, we analyzed a reasonable number of cells per condition in each experiment (14–99 independent cells; most conditions included more than 60 cells). For biochemical, transcriptomic, and lipidomic assays, we analyzed at least 3 independent experiments. These sample sizes were based on standard practice in the field and were sufficient to reveal consistent, statistically significant differences between groups.
Data exclusions	No data exclusion was performed for data analysis.
Replication	All experiments were independently conducted at least three times to confirm reproducibility. All replication attempts were successful.
Randomization	Randomization is not relevant to this study because the cells / samples in a particular condition did not exhibit differences in appearances. All cells / samples were analyzed in the same way.
Blinding	Blinding is not relevant to this study because the cells / samples in a particular condition did not exhibit differences in appearances. All cells / samples were analyzed in the same way.

Reporting for specific materials, systems and methods

We require information from authors about some types of materials, experimental systems and methods used in many studies. Here, indicate whether each material, system or method listed is relevant to your study. If you are not sure if a list item applies to your research, read the appropriate section before selecting a response.

Materials & experimental systems

n/a	Involved in the study	
☐	☒	Antibodies
☐	☒	Eukaryotic cell lines
☒	☐	Palaeontology and archaeology
☒	☐	Animals and other organisms
☒	☐	Clinical data
☒	☐	Dual use research of concern
☒	☐	Plants

Methods

n/a	Involved in the study	
☒	☐	ChIP-seq
☒	☐	Flow cytometry
☒	☐	MRI-based neuroimaging

Antibodies

Antibodies used	Anti-Actin, immunoblotting (IB), EMD Millipore, RRID: AB_2223041<br>Anti-CHMP2A, immunolabelling (IF), Proteintech, RRID: AB_2079470<br>Anti-Galectin-3 (IF), Proteintech, RRID: AB_2136768<br>Anti-LAMP1 (IF), Developmental Studies Hybridoma Bank, RRID: AB_2296838<br>Anti-LC3B (IF), Cell Signaling Technology, RRID: AB_2137707<br>Anti-mCherry (IB), Proteintech, RRID: AB_2876881<br>Anti-MTCO2 (Anti-COXII) (IB), Abcam, RRID: AB_10887758<br>Anti-NPC1 (IB), Proteintech, RRID: AB_2152050<br>Anti-OSBP (IB), Proteintech, RRID: AB_2158220<br>Anti-OSBP (IF), Sigma, RRID: AB_2676401<br>Anti-Parkin (IB), Cell Signaling Technology, RRID: AB_2159920<br>Anti-PI4KIIα (IB), Santa Cruz Biotechnology, RRID: AB_3676719<br>Anti-SREBP-2 (IB), Santa Cruz Biotechnology, RRID: AB_2194250<br>Anti-SREBP-2 (IB), MilliporeSigma, Cat# MABS1988<br>Anti-TGN46 (IF), Proteintech, RRID: AB_10597396<br>Goat anti-Mouse IgG (H+L) Secondary Antibody, Alexa Fluor 594 conjugate (IF), Thermo Fisher Scientific, RRID: AB_2534091<br>Goat anti-Mouse IgG (H+L) Secondary Antibody, Alexa Fluor 647 conjugate (IF), Thermo Fisher Scientific, RRID: AB_2535804<br>Goat Anti-Mouse IgG (H+L)-HRP Conjugate (IB), Bio Rad, RRID: AB_11125547<br>Goat anti-Rabbit IgG (H+L) Secondary Ab, Alexa Fluor 647 conjugate (IF), Thermo Fisher Scientific, RRID: AB_2535813<br>Goat Anti-Rabbit IgG (H+L)-HRP Conjugate (IB), Bio Rad, RRID: AB_11125142
Validation	Anti-Actin, immunoblotting (IB), EMD Millipore, https://www.antibodyregistry.org/AB_2223041<br>Anti-CHMP2A, immunolabelling (IF), Proteintech, https://www.antibodyregistry.org/AB_2079470<br>Anti-Galectin-3 (IF), Proteintech, https://www.antibodyregistry.org/AB_2136768<br>Anti-LAMP1 (IF), Developmental Studies Hybridoma Bank, https://www.antibodyregistry.org/AB_2296838<br>Anti-LC3B (IF), Cell Signaling Technology, https://www.antibodyregistry.org/AB_2137707<br>Anti-mCherry (IB), Proteintech, https://www.antibodyregistry.org/AB_2876881<br>Anti-MTCO2 (Anti-COXII) (IB), Abcam, https://www.antibodyregistry.org/AB_10887758<br>Anti-NPC1 (IB), Proteintech, https://www.antibodyregistry.org/AB_2152050<br>Anti-OSBP (IB), Proteintech, https://www.antibodyregistry.org/AB_2158220<br>Anti-OSBP (IF), Sigma, https://www.antibodyregistry.org/AB_2676401<br>Anti-Parkin (IB), Cell Signaling Technology, https://www.antibodyregistry.org/AB_2159920<br>Anti-PI4KIIα (IB), Santa Cruz Biotechnology, https://www.antibodyregistry.org/AB_3676719<br>Anti-SREBP-2 (IB), Santa Cruz Biotechnology, https://www.antibodyregistry.org/AB_2194250<br>Anti-SREBP-2 (IB), MiliporeSigma, https://www.sigmaaldrich.com/SG/en/product/mm/mabs1988?<br>srsltid=AfmBOopWEx9NrVoxgc6LW3oJjEQ_9NnuRdpW1uaBw_i8XhVsgUhO9qLo<br>Anti-TGN46 (IF), Proteintech, https://www.antibodyregistry.org/AB_10597396

Eukaryotic cell lines

Policy information about cell lines and Sex and Gender in Research

Cell line source(s)	HeLa (ATCC®); COS-7 (ATCC®); HEK293T (ATCC®); U2OS (ATCC®); NIH3T3 (ATCC®); N/TERT-1 (Jim Rheinwald).
Authentication	The cell line showed characteristic morphology. No further authentication was performed.
Mycoplasma contamination	All cell lines used in this study were tested negative for mycoplasma contamination.
Commonly misidentified lines (See ICLAC register)	None.

Plants

Seed stocks	Not applicable.
Novel plant genotypes	Not applicable.
Authentication	Not applicable.
