# Supplementary material for: Cholesterol maintains the degradative capacity of lysosomes during clearance and recycling of dysfunctional mitochondria
Source: Nat Commun. 2026 Sep 16;17:9647. doi: 10.1038/s41467-026-77423-1 (PMC13582898; doi:10.1038/s41467-026-77423-1)
Supplement: Supplementary file 4 [file 41467_2026_77423_MOESM5_ESM.pdf]

nature portfolio

Peer Review File

Cholesterol maintains the degradative capacity of lysosomes during clearance and recycling of dysfunctional mitochondria

Corresponding Author: Dr Yasunori Saheki

This file contains all reviewer reports in order by version, followed by all author rebuttals in order by version.

Version 0:

Reviewer comments:

Reviewer #1

(Remarks to the Author)
In this manuscript, the authors investigate the role of cholesterol in lysosomal function during mitophagy. They report that cholesterol accumulates on lysosomal membranes in response to mitophagy, a process mediated by PI4K2A-driven production of PI4P. This PI4P pool facilitates OSBP-dependent cholesterol transfer from the ER to lysosomes. The authors propose that this cholesterol accumulation enhances lysosomal membrane stability, thereby supporting the efficient degradation of damaged mitochondria. Additionally, they show that lipid droplets form as a downstream consequence of mitochondrial breakdown, attributed to the release and storage of free fatty acids. The authors conclude that cholesterol plays a crucial role in preserving the degradative capacity and structural integrity of lysosomes during mitophagy, with potential implications for cellular homeostasis and neurodegenerative disease.
While the study is conceptually interesting, several key aspects lack novelty. The role of cholesterol in maintaining lysosomal membrane integrity has been previously reported, and the idea that lysosomal lipid composition affects degradative function is not new. Furthermore, while lysosomal cholesterol accumulation is demonstrated, the degradation of mitochondria itself is not shown robustly enough to support the strength of the conclusions. Additional evidence is needed to demonstrate that mitophagy proceeds fully and that mitochondrial clearance is indeed dependent on the proposed cholesterol–PI4K2A axis.

Major points:

1. One of the main conceptual concerns is the rationale behind cholesterol accumulation on lysosomes that are not damaged. Why would intact lysosomes require additional cholesterol? Is this a mitophagy-specific phenomenon, or does it occur more broadly during other forms of selective autophagy? Please address and discuss. To strengthen the specificity of the findings, I recommend that the authors include an additional, mechanistically distinct mitophagy model, such as deferiprone (DFP) treatment, in addition to the ones presented in the figure S1.
2. The authors propose that mitophagy induction with O/A leads to cholesterol accumulation on lysosomes that are not damaged, via a PI4K2A–PI4P–OSBP-mediated pathway. However, the mechanistic basis of this signaling cascade remains unclear. Specifically, how do depolarized mitochondria communicate with the ER to trigger cholesterol transfer to lysosomes? What kickstarts this inter-organelle signaling, and what cues ensure that only non-damaged lysosomes receive cholesterol? Clarifying this pathway is essential to support the proposed model.
3. Please demonstrate cholesterol accumulation in lysosomes directly by isolating lysosomes and performing lipidomic analyses.
4. To ensure robustness and specificity of your findings, please use multiple independent lipid sensors for cholesterol and PI4P. This will strengthen the conclusions and address potential probe-specific artifacts. Co-staining with lysosomal and mitochondrial markers is also essential for subcellular localization.
5. Figures 1c–d- To support the claim that dysfunctional mitochondria are degraded within functional lysosomes, please perform live-cell imaging with high-resolution time-lapse to visualize mitochondrial degradation events. Quantify the events. Additionally, include quantification of colocalization between relevant markers. Notably, in the lower left cell of panel 1c, there appears to be no clear EGFP-GRAM-W signal despite strong Tom20 staining. This discrepancy should be addressed.
6. Figure 1d- Please report the percentage of LAMP1-positive lysosomes that contain engulfed mitochondria to support the claim of lysosomal degradation of mitochondria.
7. Figure 1f- The interpretation here appears overextended. Please apply stereological analysis to quantify CLEM images in an unbiased and reproducible manner.

8. The statement in figure legends that "data are pooled from one experiment" is unclear and concerning. If this means that an experiment was performed only once, the findings may be overinterpreted. Please clarify this and ensure that all key conclusions are based on biological replicates from several independent experiments.
9. Since the manuscript discusses temporal regulation of lipid signaling and organelle interactions, live-cell imaging using multiple markers (e.g., for PI4P, lysosomes, mitochondria) is necessary. For example, in Fig. S2b, EGFP-GRAM-W signal appears reduced after 20 min of O/A treatment. Please quantify the number and intensity of vesicles over time and relate this to lysosome–mitochondria dynamics.
10. Figure 2a- PI4P appears recruited in PI4K2A KO cells upon O/A treatment. Please explain this observation. What is the source of PI4P if PI4K2A is reported to be the sole contributor?
11. Please evaluate lysosomal pH over time after O/A treatment and not just recovery using the pH-sensitive probe in live cells. This is essential to support claims regarding lysosomal functional integrity.
12. GAL3 is a well-established marker of large-scale lysosomal rupture, but it may miss subtle membrane damage. To capture early and small-scale damage, please include CHMP4B localization as an ESCRT-III marker with faster recruitment kinetics (see Skowyra et al., Science, 2018). This additional analysis would clarify whether the lysosomes are truly undamaged or undergoing sub-lethal repair.
13. Fig.5- Authors report that lysosomal pH increases after O/A treatment (+24 h recovery). However, LLOMe requires acidic pH for activation. This raises the possibility that low levels of lysosomal damage in O/A + LLOMe conditions may result from impaired LLOMe activity due to elevated pH. Please address this potential confounding factor.
14. Given the importance of ER–lysosome contact sites and OSBP-mediated lipid transfer, it is essential to examine how ER morphology and dynamics respond to O/A treatment. Please include live-cell imaging of ER structure over time following O/A exposure.

Minor points:

1. Figure 1- At later points following O/A treatment, particularly after 2h and 4h washout, cholesterol appears to accumulate at the plasma membrane. Please clarify this observation and discuss its potential relevance.
2. 3. Figure 1b- Quantify the number of vesicles per cell to provide a clearer picture of vesicular dynamics following O/A treatment.
3. 10. Figure 2e- Include quantitative colocalization analysis between PI4P and lysosomal markers to support your interpretations.

Reviewer #2

(Remarks to the Author)
In their manuscript, Yang and co-workers describe an interesting new mechanism in which cholesterol build-up in lysosomal membranes enables the upkeep of their degradative capacity and membrane integrity during mitochondrial degradation. Their data point to an initial PI4P production that activates OSBP for cholesterol to be transported from the ER to the lysosomes, causing the ER to get depleted from cholesterol. This, in turn, activates SREBP2-linked de novo cholesterol synthesis. When lysosomes function well, the mitochondrial lipids are effectively broken down in fatty acids which they show are converted to TAG for storage in lipid droplets. While some mechanistic aspects of the paper have been described before, the authors succeeded in connecting/dissecting in much mechanistic detail to explain how lysosomes maintain their integrity/function while 'consuming' an overload of mitochondrial membranes. I am not aware of any direct prior work that would have shown a similar complete proposed axis related to mitochondrial degradation. Overall the technical and experimental quality of the paper is high and very well controlled by independent and/or orthogonal approaches making it a convincing story suitable for publication. I have only some limited remarks and suggestions that the authors must address.

Major remarks:
- The authors performed some TEM analysis of the cells (Fig. 1f, 6j). However, none of these are showing or connecting to the lipid droplet (LD) phenotype, being the proposed end-stage of the mitochondrial lipid content by the authors. In this regard, different studies have detected that the accumulation of cholesterol might be linked to dysfunctional lysosomes in neurodegenerative diseases and described the formation of multilamellar bodies (MLB) instead, also specifically linked to mitochondrial/mitophagy defects (PMID: 29368986; PMID: 15840653, PMID: 37225734). Would there be a threshold of beneficial and detrimental cholesterol accumulation, leading to either LD or MLB? Can the authors discuss or investigate?
- Of note in this regard: "Fig. 6j. Transmission electron microscopy images of N/TERT cells with indicated treatment. Yellow arrowheads indicate lipid droplets. N, nucleus. PM, plasma membrane. Scale bars, 1 µm." To my opinion the arrowheads rather indicate degradative mature lysosomes (typically very dark to black content at the EM level), not lipid droplets. Lipid droplets typically appear as white to fuzzy light-grey organelles with a lipid monolayer on transmission electron microscopy (TEM), not as electron-dense vesicles as indicated here. Different examples can be found in literature, e.g. PMID: 31959936, showing both lipid droplets and lysosomes. Could the authors provide broader images of the cells as currently the case and use insets to highlight clear/correctly identified lipid droplets, if present? There is also no dedicated description on how the TEM was done to visualize LDs in the M&M section (only CLEM is described). Please include more details on the EM processing.
- Fig. 5C. It was recently shown that calcium dysregulations would be upstream of acidification defects (DOI/10.1083/jcb.201201076DOI: 10.7554/eLife.15887, DOI: 10.1016/j.devcel.2024.03.030, DOI: 10.1073/pnas.2211999119). Changes in the levels of calcium and disruptions in the calcium-gradient can hinder the acidity of lysosomes by reducing the force that drives the influx of hydrogen ions, thus preventing the restoration of lysosomal function. (DOI: 10.3390/cells9122655). Some papers further detect calcium defects without pH changes e.g. in Niemann-Pick disease type C1 (DOI: 10.1038/nm.1876). Given the importance of calcium to the ER (and lysosome) and the link to

cholesterol, could the authors investigate to what extent lysosomal Calcium is affected and could contribute to the observed defects. Currently the mechanism is limited to a potential role of cholesterol in the assembly of V-ATPase.
- The discussion section primarily includes a list of five major conclusions, which are then repeated in the next paragraphs. This results in repetition and a lack of integration with the existing literature; for example, the final paragraph on page 16 (refs. 71-85) mentions multiple research articles without completely contextualizing them. The discussion might benefit from less repetition and a more thorough integration of the findings into the larger scientific context. For instance, several aspects of this mechanistic cascade may fail (for different reasons) in the context of neurodegenerative diseases, including Alzheimer's and Parkinson (lysosomal dyshomeostasis, mitochondrial dysfunction, lipid metabolism dysregulations), and wherein lysosomal abnormalities are increasingly taking in a central position (also based on genetics with many risk genes related to endolysosomal/autophagy functions in AD, PD as well as ALS and spastic paraglegia (SP)). Several risk/causal proteins are also linked to ER-Lys MCSs, such as VAPB (ALS), protrudin (SP), VPS13C (PD/DLB; doi.org/10.1186/s40478-021-01121-w; doi.org/10.1016/j.ajhg.2016.01.014) and recently APP (doi.org/10.1016/j.devcel.2024.03.030) suggesting failing MCSs could be a hub for initiating lysosomal dyshomeostasis (reviewed recently in DOI: 10.1002/bies.202400023).

Small remarks:
- The authors should indicate the specific statistical values instead of working with ****
- Fig. 1D. Could the authors quantify if lysosomes containing mitochondria have a higher cholesterol/GRAM signal? As this would provide more strength to the causality claim. If not, it could be a downstream effect. As with Fig. 2a, please conduct the analysis within each condition individually, rather than limiting the comparison to control versus O/A.
- Fig. S2b: "Importantly, this PI4P accumulation preceded that of cholesterol, indicating that PI4P may drive the accumulation of cholesterol on degradative lysosomal membranes during mitophagy (Figure S2b).". Can the authors provide zooms to clearly substantiate the claim? At 20, we can already see a green spot right upper quadrant that is first green before magenta, idem for a vesicle left below corner at 40. Maybe this is just less clear without zooms provided.
- Is it possible that figure 6, panels k and l are not discussed/referred to in the Result section?
- Could the authors list which reference genome (version) was used for the mRNA-seq?
- Data availability. The authors should deposit their RNA and lipidomics data to open repositories like MetaboLights or the Metabolomics Workbench.
- Could the authors provide information on which internal standards were used (types, quantities) for the lipidomic analysis?

Reviewer #3

(Remarks to the Author)
In this study, Yang and colleagues identified cholesterol as a critical regulator maintaining lysosomal functional integrity during the clearance and recycling of dysfunctional mitochondria. They found that mitochondrial depolarization triggers acute cholesterol delivery from the ER to degradative lysosomal membranes, mediated by PI4P-driven, OSBP-dependent transport. This massive cholesterol efflux from the ER activates SREBP-2, driving cholesterol biosynthesis and ultimately lipid droplet (LD) formation—revealing cholesterol's pivotal role in maintaining cellular homeostasis during organelle stress. The data mostly supports the conclusions and there are a number of additional experiments I think would strengthen the conclusions:

Major points:
1. Most quantifications are not properly done: statistics should be performed between independent samples with the sample size to at least 50 cells per group per biological replicate. The experiments should be repeated at lease three times and statistics performed between experiments (using the mean of several cells quantified per experiment).
2. In Figure 1e, they demonstrated that EGFP-GRAM-W progressively accumulates in TOM20-mSc-labeled mitochondria, yet the majority of cholesterol pools showed distinct localization patterns, what functional roles might these non-mitochondrial cholesterol pools play.
3. In Figure 2, genetic ablation of PI4P and OSBP would help determine whether O/A-induced cholesterol enrichment on lysosomal membranes depends on this transport pathway. They should also verify PI4P levels regulated by OSBP. A comparative analysis of cholesterol and PI4P distribution between trans-Golgi and lysosomes, both pre- and post-OA treatment, would reveal potential trafficking dynamics.
4. In Figure 3, genetic knockout of SREBP-2 should be performed to validate its role in regulating both cholesterol content and PI4P expression on lysosomal membranes. Whether PI4KIIα/PI4P KO inhibited SREBP-2 cleavage?
5. In Figure 4, they used a dTAG system to induced OSBP degradation, why not directly knockout the OSBP gene? Is there molecular evidence for direct interaction between OSBP and SREBP2?
6. In Figure 5, while lysosomal pH measurement provides one indicator of degradative capacity, additional assays would provide more comprehensive functional characterization.
7. In Figure 6, the study demonstrates O/A-induced LD accumulation, but the functional relationships between these LDs and (1) lysosomal cholesterol/PI4P dynamics, and (2) SREBP2 activation remain unclear. Importantly, does OSBP inhibition attenuate LD biogenesis, potentially linking these pathways.
8. In Figure 7, they used NPC1 KO cells to verify LD formation, further confirming that the degradative function of lysosomes is a key requirement for LD biogenesis following mitochondrial depolarization, PI4P/OSBP/PI4KIIα-KO cells should also be used to verify LD formation.

Reviewer #4

(Remarks to the Author)
I co-reviewed this manuscript with one of the reviewers who provided the listed reports. This is part of the Nature Communications initiative to facilitate training in peer review and to provide appropriate recognition for Early Career Researchers who co-review manuscripts.

Version 1:

Reviewer comments:

Reviewer #1

(Remarks to the Author)
The authors have adequately addressed my key concerns raised during review, and I consider the manuscript suitable for publication.

Reviewer #2

(Remarks to the Author)
Yang et al have submitted a revised manuscript on the role of cholesterol in maintaining lysosomal degradative capacity during mitophagy. In general the authors have substantially improved their paper with the addition of multiple novel datasets, based on the reviewers' comments. The novel data further strengthen the proposed working mechanism of cholesterol. I am very satisfied on how the authors addressed all critiques and improved their manuscript both qualitatively and quantitatively with these new data. I have only a few small remarks that the authors should comment or adjust in their manuscript, but which would otherwise not question my recommendation for publication.
- In Fig. 1e (and suppl movie 1), an example is shown for the accumulation of chol in degradative lysosomal membranes. The authors state that chol accumulates from the moment mitophagy is initiated, but this is an example of a degradative lysosome that already contains Tomm20 staining at the beginning of O/A treatment (so, chol should be already on it). Is there no example where a degradative lyso becomes positive for tomm20 (does receives mito for degradation) and next accumulates chol?
- Related to Fig. 5e-f, the text states that 'additional treatment of cells with O/A or mevastatin dramatically enhanced lysosomal membrane rupture', but this doesn't differentiate for the protective role of chol in control cells with LLOME treatment. From the data it is clear that in control cells LLOME increased fragility in O/A treated cells but this was more dramatically when cells were depleted from cholesterol. The authors could adjust the text to highlight this difference.
- In Fig. 7, the authors demonstrate that LD formation is reduced in O/A treated cells when SCAP is inhibited, in mevastatin treated cells, when PI4KIIa is knocked out, or following inhibition of OSBP by OSW-1, showing that chol is crucial for mitophagy-induced LD formation. However, when control cells are treated with OSW-1, this also resulted in increased LD levels: in this case, the authors argue that instead of storage of FAs, this is caused by chol accumulation in the ER where 'excess chol is esterified and stored in LDs'. If true, these LDs should be positive for a cholesterol marker (GRAM-W of filipin)? And second, if a blockade in chol transport to lyso increases LDs, shouldn't that be the case as well for the other treatments under O/A conditions? Or is the flux of FAs more dominant compared to chol in these cases. Could the authors comment or clarify this further?
- Ref doi: 10.3389/fnins.2022.900338 is a more updated review on MCSs in neurodegenerative diseases.

Reviewer #3

(Remarks to the Author)
Overall, the revised manuscript is rigorous, complete, and provides significant new insights. It is suitable for potential publication. I have four suggestions for further strengthening the study:
(1) The authors report that complete loss of OSBP, PI4P, and SREBP-2 is lethal. Would transient siRNA knockdown also be lethal? If viability is retained, such knockdowns could serve as orthogonal, genetically defined validation of the key pharmacological findings.
(2) The authors show cholesterol depletion impairs lysosomal function. Restoring cellular cholesterol levels could test whether enhanced cholesterol availability rescues lysosomal degradative capacity.
(3) The functional role of LD formation during mitophagy remains incompletely defined. Does LD accumulation act as a regulatory node—for instance, by sequestering excess cholesterol and thereby attenuating lysosomal cholesterol overload—or does it represent a passive, stoichiometric sink? Clarifying whether LDs exert active feedback on lysosomal cholesterol dynamics would critically inform their proposed role in modulating the mitophagy–lysosome axis.
(4) Direct visualization and tracking of lipid trafficking would substantially strengthen the proposed model of inter-organellar lipid recycling. Specifically, pulse-labeling of mitochondrial membrane lipids could enable quantitative assessment of their transfer to lysosomes and subsequent incorporation into LDs. Similarly, ER-localized cholesterol could be selectively labeled to trace its mobilization to lysosomal membranes. Such approaches would provide definitive, spatially resolved evidence for the hypothesized lipid flux pathway.

Reviewer #4

(Remarks to the Author)
I co-reviewed this manuscript with one of the reviewers who provided the listed reports. This is part of the Nature Communications initiative to facilitate training in peer review and to provide appropriate recognition for Early Career Researchers who co-review manuscripts.

Version 2:

Reviewer comments:

Reviewer #2

(Remarks to the Author)
The comments raised in the previous round of review have been adequately addressed by the authors. Additional experiments were added in the revised manuscript to support the central conclusions . The authors gave clear and appropriate answers to the remaining questions . The few points that could not be addressed experimentally are sufficiently acknowledged and justified as technical limitations. In conclusion, I think the manuscript has been significantly improved and is now ready for publication in Nature Communications.

Reviewer #3

(Remarks to the Author)
All my concerns have been addressed, I have no further questions.

Reviewer #4

(Remarks to the Author)
I co-reviewed this manuscript with one of the reviewers who provided the listed reports. This is part of the Nature Communications initiative to facilitate training in peer review and to provide appropriate recognition for Early Career Researchers who co-review manuscripts.

Open Access This Peer Review File is licensed under a Creative Commons Attribution 4.0 International License, which permits use, sharing, adaptation, distribution and reproduction in any medium or format, as long as you give appropriate credit to the original author(s) and the source, provide a link to the Creative Commons license, and indicate if changes were made.
In cases where reviewers are anonymous, credit should be given to 'Anonymous Referee' and the source.
The images or other third party material in this Peer Review File are included in the article's Creative Commons license, unless indicated otherwise in a credit line to the material. If material is not included in the article's Creative Commons license and your intended use is not permitted by statutory regulation or exceeds the permitted use, you will need to obtain permission directly from the copyright holder.
To view a copy of this license, visit https://creativecommons.org/licenses/by/4.0/

Nature Communications manuscript NCOMMS-25-49554-T

REBUTTAL TO THE COMMENTS RAISED BY THE REVIEWERS

We thank the editor and the reviewers for the constructive comments and suggestions. We have carried out extensive new experimentation to address all the concerns and made necessary changes to the texts. We have now:

1) Repeated all our major imaging experiments, conducted rigorous quantification, and significantly expanded biological replicates and sample size, following suggestions by Reviewers #1 and #3.
2) Performed detailed analysis of 1,831 lysosomes labelled with or without the cholesterol biosensor EGFP-GRAM-W and the mitochondrial marker Tom20-mScarlet (**new Table S1**), providing evidence for the delivery of damaged mitochondria into lysosomes and supporting that lysosomes containing damaged mitochondria are more frequently associated with elevated membrane cholesterol (for Reviewers #1 and #2).
3) Performed additional correlative light and electron microscopy (CLEM) imaging to confirm the identity of degradative lysosomes (**new Figure 1f**) and lipid droplets (**new Figure 6j**) (for Reviewers #1 and #2).
4) Included additional probes to examine cholesterol (purified mCherry-D4H; **new Supplementary Figures 1c and 1d**) and PI4P (PH domain of FAPP1; **new Supplementary Figures 2b and 2c**) to strengthen our conclusions (for Reviewer #1).
5) Provided functional evidence supporting the role of cholesterol in mitochondrial degradation during mitophagy by using Parkin overexpression and showing that degradation of MTCO2 (COXII), an inner mitochondrial protein degraded through autophagy during depolarization-induced mitophagy, is less efficient when cellular cholesterol levels are lowered (**new Figures 5a and 5b**) (for Reviewers #1 and #3).
6) Provided additional evidence supporting the importance of lysosomal membrane cholesterol in lipid droplet formation following mitophagy by demonstrating that lipid droplet formation is suppressed by PI4KIIα knockout, inhibition of SREBP-2 cleavage, or inhibition of OSBP (**Figure 7 and Supplementary Figure 7**) (for Reviewer #3).

A specific list of changes is indicated below, followed by a point-by-point rebuttal from page 3.

<u>Additional replicates and increased sample sizes, as suggested by the reviewers, are provided for the following figures (A list of detailed changes is included in the response to Reviewer #1, Comment 8 and Reviewer #3, Comment 1):</u>

Figure 1b, Figure 1h, Figure 2b, Figure 2f (previously Figure 2d), Figure 2i (previously Figure 2g), Figure 2k (previously Figure 2i), Figure 3h, Figure 5d, Figure 5f, Figure 6i, Figure 6l, Figure 7b, Figure 7d, Figure 7f, Supplementary Figure 7b, Supplementary Figure 7d, and Supplementary Figure 7e

<u>The following data are newly included:</u>

CLEM to confirm the identities of degradative lysosomes and lipid droplets

- **Figure 1f:** Replaced with new CLEM images showing a degradative lysosome additionally labelled with LysoTracker Deep Red. Additional examples are provided in the response to Reviewer #1, Comment 7.
- **Figure 6j:** Replaced with new CLEM images showing increased lipid droplets following O/A treatment using N/TERT cells stably expressing mCherry-ADRP.

Data using alternative cholesterol and PI4P probes

- **Supplementary Figures 1c and 1d:** Showing the increase of cholesterol levels on lysosomal membranes in O/A-treated cells, as detected by purified mCherry-D4H proteins.

- **Supplementary Figures 2b and 2c:** Showing the increase of PI4P levels on lysosomes in O/A-treated cells, using EGFP-tagged PH domain of FAPP1.

Immunoblot analysis of mitochondrial degradation following mitophagy induction

- **Figures 5a and 5b:** Establishing N/TERT cells stably expressing BFP-tagged Parkin and showing that MTCO2 degradation is less efficient under cholesterol-depleted conditions compared with non-depleted conditions, replacing our previous data based on Magic Red.

Dependency of O/A-induced lipid droplet formation on PI4P-OSBP-cholesterol axis

- **Figures 7g and 7h:** Showing the suppression of O/A-induced lipid droplet formation in PI4KIIα knock out cells.
- **Figures 7i and 7j:** Showing the suppression of O/A-induced lipid droplet formation in cells treated with the OSBP inhibitor, OSW-1.
- **Supplementary Figures 7i and 7j:** Showing the suppression of O/A-induced lipid droplet formation in cells treated with the SCAP inhibitor, UT-59, which blocks SREBP-2 cleavage.

Additional data to further strengthen our conclusions

- **Supplementary Figure 1f:** Showing the absence of lysosomal membrane damage in O/A-treated cells, as assessed using an anti-CHMP2A antibody.
- **Supplementary Figures 1i and 1j:** Showing the increase of cholesterol levels on lysosomal membranes in DFP-treated cells, but not in HBSS-treated cells, as detected using EGFP-GRAM-W.
- **Figures 2c and 2d:** Showing a time-course analysis of the increase in cholesterol and PI4P on lysosomes following O/A treatment, as assessed using EGFP-GRAM-W and dTomato-ORP9-PH, respectively, with lysosomes labelled by LAMP1-miRFP703.
- **Supplementary Figures 2b and 2c:** Showing the accumulation of PI4P on lysosomes following O/A treatment, as assessed using another PI4P biosensor, EGFP-FAPP1-PH, with lysosomes labelled by LAMP1-miRFP703.
- **Supplementary Figures 2f and 2g:** Showing the dependence of mitophagy-dependent OSBP recruitment to lysosomes on PI4KIIα.
- **Supplementary Figure 2h:** Showing the morphological changes of the endoplasmic reticulum upon O/A treatment.

The following data are provided for the reviewers' reference only.

- **Appendix Figure 1-1:** Showing that at 2 hours of treatment, only O/A, but not DFP or nutrient starvation, induced accumulation of cholesterol on lysosomal membranes.
- **Appendix Figure 1-2:** Showing the distribution of cholesterol and PI4P in cells with markers for lysosomes (LAMP1) and mitochondria (TOM20).
- **Appendix Figure 1-3:** Showing additional CLEM images of degradative lysosomes.
- **Appendix Figure 1-4:** Showing lysosomal pH over time following O/A treatment.
- **Appendix Figure 2-1:** Showing measurements of lysosomal Ca^{2+} levels in cells treated with O/A in the presence or absence of mevastatin.
- **Appendix Figure 3-1:** Showing increased lysosomal PI4P levels upon OSBP inhibition.
- **Appendix Figure 3-2:** Showing cholesterol and PI4P levels on lysosomal membranes and the TGN before and after O/A treatment.
- **Appendix Figure 3-3:** Showing cholesterol and PI4P levels on lysosomal membranes upon treatment with the SCAP inhibitor, UT-59.
- **Appendix Figure 3-4:** Showing a comparison of O/A-induced SREBP-2 cleavage in wild-type and PI4KIIα KO cells.

-Sentences from the reviewers' comments are *in italics*
-Our responses are in blue

Reviewer #1 (Remarks to the Author):

In this manuscript, the authors investigate the role of cholesterol in lysosomal function during mitophagy. They report that cholesterol accumulates on lysosomal membranes in response to mitophagy, a process mediated by PI4K2A-driven production of PI4P. This PI4P pool facilitates OSBP-dependent cholesterol transfer from the ER to lysosomes. The authors propose that this cholesterol accumulation enhances lysosomal membrane stability, thereby supporting the efficient degradation of damaged mitochondria. Additionally, they show that lipid droplets form as a downstream consequence of mitochondrial breakdown, attributed to the release and storage of free fatty acids. The authors conclude that cholesterol plays a crucial role in preserving the degradative capacity and structural integrity of lysosomes during mitophagy, with potential implications for cellular homeostasis and neurodegenerative disease. While the study is conceptually interesting, several key aspects lack novelty. The role of cholesterol in maintaining lysosomal membrane integrity has been previously reported, and the idea that lysosomal lipid composition affects degradative function is not new. Furthermore, while lysosomal cholesterol accumulation is demonstrated, the degradation of mitochondria itself is not shown robustly enough to support the strength of the conclusions. Additional evidence is needed to demonstrate that mitophagy proceeds fully and that mitochondrial clearance is indeed dependent on the proposed cholesterol–PI4K2A axis.

Thank you very much for all the constructive comments, which have helped us further improve our manuscript. In the revised manuscript, we addressed the issue of membrane damage by using an additional marker, CHMP2A, and confirmed that lysosomal membrane damage is not induced during mitophagy (**new Figure S1f**), in contrast to previous studies examining lysosomal membrane integrity, which involves lysosomal membrane rupture induced by LLOMe treatment or other related methods. We would also like to clarify that, although the role of some lipids, including phosphoinositides, in regulating lysosomal degradative function has been previously reported (as discussed in the Introduction of our original manuscript), the role of cholesterol in this process has remained elusive. As detailed below in our response, there are technical challenges in directly detecting the degradation of damaged mitochondria using live cell imaging. Nevertheless, we now provide additional evidence demonstrating the delivery of damaged mitochondria into lysosomes and show that lysosomes containing damaged mitochondria are more frequently associated with elevated membrane cholesterol (**new Table S1**). Using Parkin overexpression, we further showed that degradation of COXII, an inner mitochondrial protein targeted by autophagy during depolarization-induced mitophagy, is reduced under conditions of cholesterol depletion (**new Figures 5e and 5f**). Collectively, these findings support our model in which enrichment of cholesterol on degradative lysosomal membranes promotes the efficient clearance of damaged mitochondria.

We provide detailed point-by-point responses to each of the reviewer's comments below.

Major points:

1. One of the main conceptual concerns is the rationale behind cholesterol accumulation on lysosomes that are not damaged.

Why would intact lysosomes require additional cholesterol?

Reply: Thank you very much for these comments. As discussed in our original manuscript, the additional delivery of cholesterol to lysosomal membranes may strengthen their structure and maintain the degradative function of lysosomes during mitophagy. To support this model, we included the following data in the original manuscript. First, as shown in **Figures 5e and**

5f, cholesterol is required for maintaining lysosomal membrane integrity: in its absence, lysosomal membranes become fragile and more susceptible to damage induced by LLOMe. Second, as shown in **Figures 5c and 5d**, cholesterol contributes to the maintenance of lysosomal pH during mitophagy. As discussed in the original manuscript, this effect may be mediated through regulation of V-ATPase assembly at lysosomal membranes, as cholesterol has been reported to influence V-ATPase assembly at the Golgi (PMID: 40841558) (page 17). The precise mechanisms underlying cholesterol-dependent regulation of lysosomal pH are beyond the scope of the current study.

Is this a mitophagy-specific phenomenon, or does it occur more broadly during other forms of selective autophagy? Please address and discuss. To strengthen the specificity of the findings, I recommend that the authors include an additional, mechanistically distinct mitophagy model, such as deferiprone (DFP) treatment, in addition to the ones presented in the figure S1.

Reply: Thank you very much for these suggestions. To examine specificity, we tested nutrient starvation using Hanks' balanced salt solution (HBSS) to induce nutrient deprivation-dependent autophagy. In contrast to cells treated with oligomycin and antimycin for 2 hours (a condition that induces depolarization-induced mitophagy and leads to robust cholesterol accumulation on degradative lysosomal membranes labelled by LAMP1-miRFP703), cells treated with HBSS for 2 hours did not show cholesterol accumulation on degradative lysosomal membranes (**Appendix Figure 1-1**). Cells treated with HBSS for 24 hours displayed some cholesterol accumulation on vesicular structures; however, these structures were not labelled by LAMP1-miRFP703, suggesting that cholesterol primarily accumulated on non-lysosomal compartments under these conditions (**new Figure S1j**). We also tested DFP treatment, as suggested by the reviewer, to induce a mechanistically distinct form of mitophagy. Similar to HBSS, cells treated with DFP for 2 hours did not show major cholesterol accumulation on degradative lysosomes (**Appendix Figure 1-1**). Notably, cells treated with DFP for 24 hours showed robust cholesterol accumulation on massively enlarged lysosomes that were labelled by LAMP1-miRFP703 (**new Figure S1i**). Taken together, these results suggest that cholesterol accumulation on lysosomal membranes occurs strongly during mitophagy in general, although we cannot fully exclude the possibilities that it may also occur in other forms of selective autophagy.

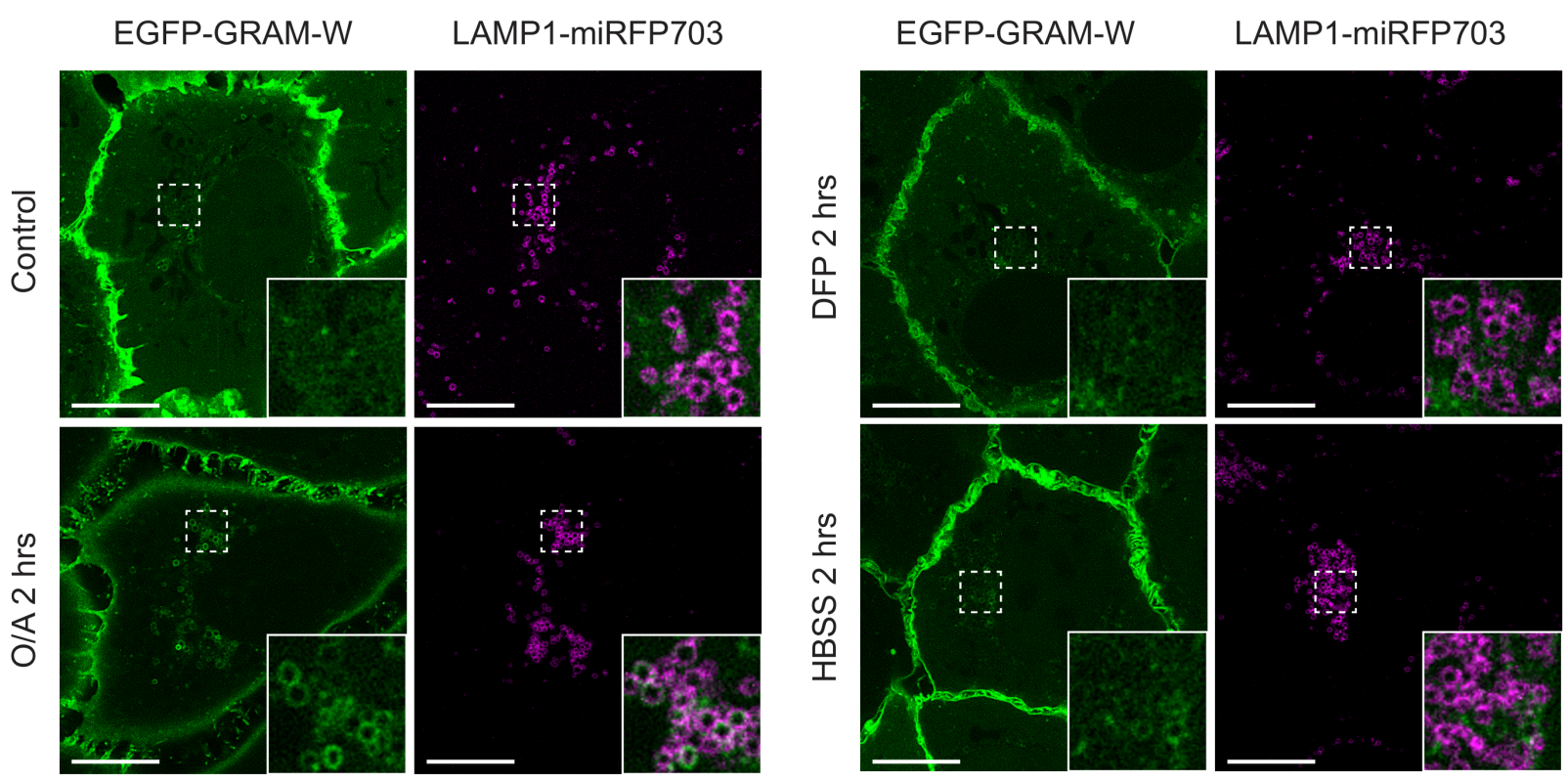

Appendix Figure 1-1
SDC-SIM images of N/TERT cells stably expressing EGFP-GRAM-W and LAMP1-miRFP703. Cells were untreated (Control) or treated with 10 µM oligomycin and 4 µM antimycin (O/A), 1 mM deferiprone (DFP), or cultured in Hank's balanced salt solution (HBSS) for 2 hours. Insets show at higher magnification the regions indicated by white dashed boxes. Scale bars, 10 µm.

We have added the following sentences in the Results section to describe the new data (Page 6):

> *"To determine whether the observed cholesterol accumulation is specific to depolarization-induced mitophagy, deferiprone (DFP) was used to induce a mechanistically distinct form of mitophagy via iron-depletion[52]. N/TERT cells treated with DFP for 24 hours showed major accumulation of cholesterol on enlarged lysosomes that were labelled by LAMP1-miRFP703 (Figure S1i), suggesting that*

mitophagy triggers cholesterol accumulation on degradative lysosomal membranes irrespective of the upstream mechanism.

Finally, we investigated whether lysosomal cholesterol accumulation is a general feature of autophagy. To this end, N/TERT cells were starved using Hank's balanced salt solution (HBSS) to trigger nutrient deprivation-dependent autophagy. Cells treated with HBSS for 24 hours showed some accumulation of cholesterol on vesicular structures. However, they were not labelled with LAMP1-miRFP703, suggesting that the cholesterol accumulation in this condition primarily occurred on non-lysosomal compartments (Figure S1j).”

We have added the following sentences in the Discussion section (Page 16):

“Interestingly, while nutrient starvation has been reported to activate PI4KIIα on degradative lysosomes, it did not induce major cholesterol accumulation on degradative lysosomes[19]. These results suggest that the accumulation of cholesterol on lysosomal membranes occurs strongly during mitophagy, although the possibilities that it might also occur in other forms of selective autophagy are not fully excluded.”

2. The authors propose that mitophagy induction with O/A leads to cholesterol accumulation on lysosomes that are not damaged, via a PI4K2A–PI4P–OSBP-mediated pathway. However, the mechanistic basis of this signaling cascade remains unclear. Specifically, how do depolarized mitochondria communicate with the ER to trigger cholesterol transfer to lysosomes? What kickstarts this inter-organelle signaling, and what cues ensure that only non-damaged lysosomes receive cholesterol? Clarifying this pathway is essential to support the proposed model.

Reply: Thank you very much for these comments. We would like to clarify that our model is that lysosomal PI4P, acutely generated during mitophagy on degradative lysosomal membranes by PI4KIIα, stimulates OSBP activity at ER-lysosome contact sites. We do not propose that depolarized mitochondria directly communicate with the ER to trigger cholesterol transfer to lysosomes. Rather, our data identify PI4P generated by PI4KIIα as the key signal that recruits OSBP to ER-lysosome contact sites to promote cholesterol delivery to degradative lysosomes.

In our original manuscript, we showed that PI4KIIα knock-out (KO) supresses the mitophagy-induced increase of PI4P and cholesterol on lysosomal membranes (**Figures 2a-2d**). In the revised manuscript, we further examined the impact of PI4KIIα KO in the recruitment of OSBP to lysosomal membranes (**new Figures S2f and S2g**).

We showed in our original manuscript that OSBP can be trapped at sites where it is active by treatment with OSW-1, a chemical inhibitor of OSBP that blocks its counter-transport function; using OSW-1, we showed that OSBP was trapped on degradative lysosomal membranes during depolarization-induced mitophagy (**Figures 2j and 2k in the revised manuscript**). In our new experiments, we treated PI4KIIα KO cells with O/A and OSW-1 and found that OSBP was significantly less trapped on degradative lysosomal membranes compared to wild-type cells (**new Figures S2f and S2g**). These results are consistent with our proposed model, where PI4P generated by PI4KIIα is the major trigger to recruit and activate OSBP at ER-lysosome contact sites to promote cholesterol delivery to degradative lysosomes.

PI4KIIα has been reported to be activated on damaged lysosomal membranes (PMID: 36071159). Since O/A treatment does not induce lysosomal damage (**Figure S1e and new Figure S1f**), our results indicate that PI4KIIα can also be activated on intact lysosomal membranes. The mechanisms by which PI4KIIα is activated on these non-damaged membranes during mitophagy remains unclear and warrants future investigation.

We have added the following sentences in the Results section to describe the new data:

> *"Importantly, OSBP was significantly less trapped on degradative lysosomal membranes in PI4KIIα KO cells compared to wild-type cells (Figures S2f and S2g). These results suggest that PI4P generated by PI4KIIα is the major trigger to recruit and activate OSBP at ER-lysosome contact sites."*

3. Please demonstrate cholesterol accumulation in lysosomes directly by isolating lysosomes and performing lipidomic analyses.

Reply: Thank you very much for this suggestion. We encountered technical difficulties in conducting the suggested experiments. Despite our best efforts to generate N/TERT cells stably expressing the constructs required for Lyso-IP (3xHA-tagged TMEM192 and TMEM192 tagged with mRFP and 3xHA), we were unable to establish these cell lines, as stable expression of these proteins appeared to be lethal in N/TERT cells. Therefore, instead of performing Lyso-IP and lipidomics, we used an alternative, well-established cholesterol biosensor, D4H, to provide additional evidence of cholesterol accumulation on lysosomal membranes. Using purified mCherry-D4H proteins (PMID: 28377464; PMID: 31548609), we detected increased cholesterol on degradative lysosomal membranes in O/A-treated cells, consistent with the results obtained with EGFP-GRAM-W (**new Figures S1c and S1d**).

We have added the following sentences in the Results section to describe the new data:

> *"A significant increase in cholesterol on lysosomal membranes was also detected using purified mCherry-tagged D4H proteins, another widely used probe for accessible cholesterol[45,46] (Figures S1c and S1d)."*

4. To ensure robustness and specificity of your findings, please use multiple independent lipid sensors for cholesterol and PI4P. This will strengthen the conclusions and address potential probe-specific artifacts.

Reply: Thank you very much for these suggestions. For the cholesterol probes, in addition to GRAM-W and filipin (**Figure S1b**), we performed additional experiments using purified mCherry-D4H proteins (PMID: 28377464; PMID: 31548609). Using purified mCherry-D4H proteins, we similarly detected increased cholesterol on degradative lysosomes in O/A-treated cells (**new Figures S1c and S1d**).

We have added the following sentences in the Results section to describe the new data:

> *"A significant increase in cholesterol on lysosomal membranes was also detected using purified mCherry-tagged D4H proteins, another widely used probe for accessible cholesterol[45,46] (Figures S1c and S1d)."*

For PI4P, we initially tested mCherry-P4M (SidM) (PMID: 24711504); however, we were unable to obtain reliable results because stable expression of mCherry-P4M led to pronounced nuclear aggregation in N/TERT cells. We therefore used the PH domain of FAPP1 (PH-FAPP1) as an alternative PI4P biosensor (PMID: 15107860; PMID: 15635101).

We generated N/TERT cells stably expressing EGFP-tagged PH-FAPP1 (EGFP-PH-FAPP1) and imaged them with or without O/A treatment. In control cells, EGFP-PH-FAPP1 localized predominantly on the Golgi, as previously reported, with minimal association with vesicular structures. In contrast, in O/A-treated cells, EGFP-PH-FAPP1 was prominently detected on vesicular structures that were positive for the lysosomal marker, LAMP1 (**new Figures S2b and S2c**). These new results, together with the results obtained using the PH domain of ORP9

in our original manuscript, confirm an increase in PI4P on lysosomal membranes during mitophagy.

We have added the following sentences in the Results section to describe the new data:

> *"A significant increase in PI4P on lysosomal membranes was also detected using EGFP-tagged PH domain of FAPP1, another biosensor for PI4P[54,55] (Figures S2b and S2c)."*

Co-staining with lysosomal and mitochondrial markers is also essential for subcellular localization.

Reply: Thank you very much for the suggestion. To visualize the levels of cholesterol and PI4P on lysosomal and mitochondrial membranes, we performed new immunostaining experiments using N/TERT cells stably expressing the cholesterol biosensor EGFP-GRAM-W and the PI4P biosensor dTomato-ORP9-PH. Cells were fixed and immunostained with antibodies against mitochondrial (TOM20) and lysosomal (LAMP1) markers. Cells were treated with ethanol (Control) or O/A for 2 hours, followed by fixation and immunostaining. In control cells, little accumulation of cholesterol or PI4P was detected on lysosomes or mitochondria. In contrast, in O/A-treated cells, we observed a marked accumulation of both cholesterol and PI4P on lysosomal membranes, consistent with our original findings. Notably, no increase in cholesterol or PI4P was detected on mitochondrial membranes, confirming that the O/A-induced elevation of cholesterol and PI4P occurs primarily on degradative lysosomes (**Appendix Figure 1-2**).

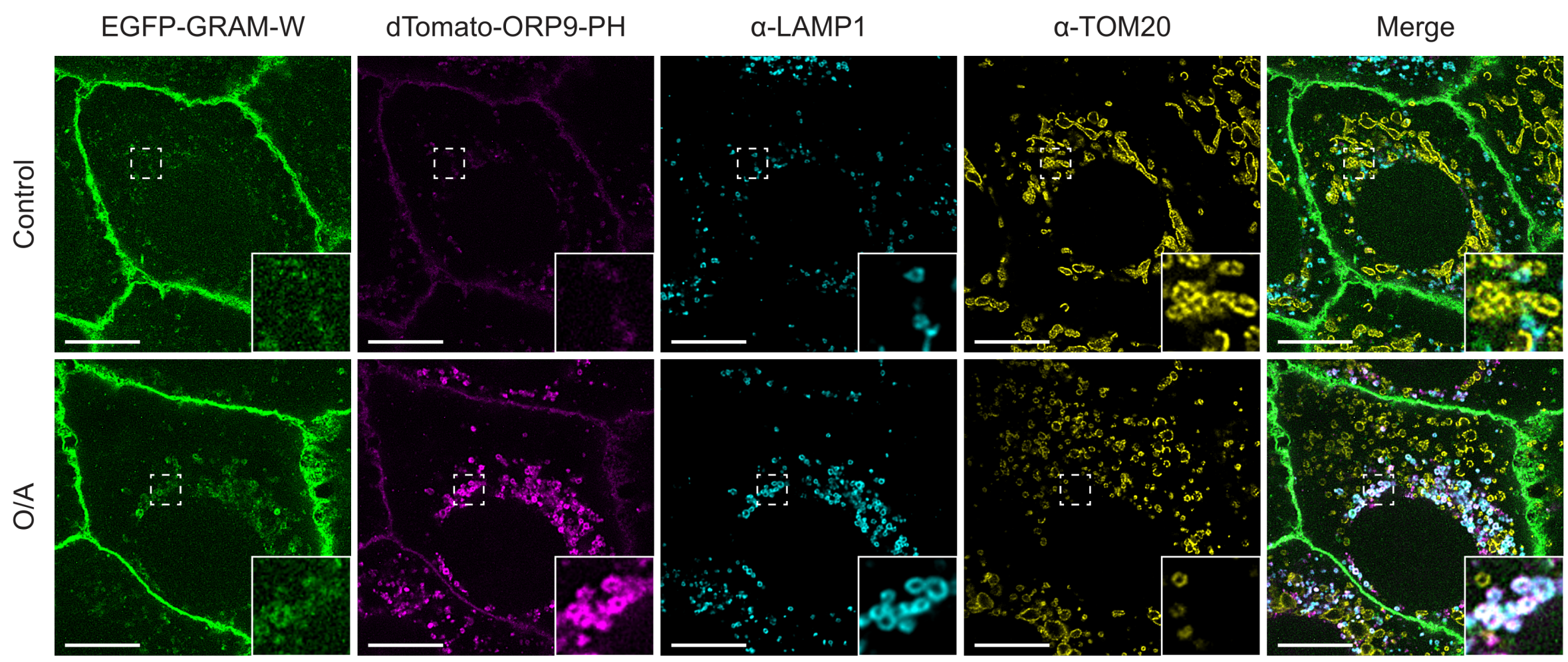

Appendix Figure 1-2
SDC-SIM images of N/TERT cells stably expressing EGFP-GRAM-W and dTomato-ORP9-PH. Cells were treated with ethanol (Control) or O/A for 2 hours and immunostained using antibodies against LAMP1 and TOM20. Insets show at higher magnification the regions indicated by white dashed boxes. Scale bars, 10 μm.

Please note that TOM20-tagged with mScarlet (Tom20-mSc) was detected inside degradative lysosomes after 2 hours of O/A treatment, as these lysosomes contained mScarlet-labelled damaged mitochondria (**Figures 1c and 1d**). Thus, mScarlet labelling allows the detection of damaged mitochondria within lysosomes, which is difficult to achieve using antibody-based approaches, likely due to the loss or alternation of immunogenicity in the lysosomal lumen.

5. Figures 1c–d- To support the claim that dysfunctional mitochondria are degraded within functional lysosomes, please perform live-cell imaging with high-resolution time-lapse to

visualize mitochondrial degradation events. Quantify the events. Additionally, include quantification of colocalization between relevant markers.

Reply: Thank you very much for the suggestion. We would like to clarify that we do not claim that mitochondria are fully degraded after 2 hours of O/A treatment. Rather, we believe that with 2 hours of O/A treatment, a subset of depolarized mitochondria is delivered to degradative lysosomes, whereas their complete degradation occurs at much later time points. We agree with the reviewer that directly visualizing mitochondrial degradation events in degradative lysosomes during mitophagy would be highly informative. However, this is technically challenging because mitophagy is a slow and gradual process; even under conditions of enforced mitophagy (e.g., Parkin overexpression combined with sustained mitochondrial depolarization), complete mitochondrial depletion can require up to 24 to 48 hours (PMID: 28005069). As a result, observing mitochondrial degradation events, such as the reduction of Tom20-tagged with mScarlet (Tom20-mSc) fluorescence within degradative lysosomes, by live imaging over a short time window of a few hours is not feasible. This is further complicated by the continuous influx of damaged mitochondria into lysosomes and the prolonged time required for their complete degradation.

As an alternative approach, we generated N/TERT cells stably overexpressing BFP-tagged Parkin and examined mitochondrial depolarization-dependent degradation of MTCO2 (COXII), an inner mitochondrial protein known to be targeted by mitophagy, using Western blotting, a well-established method in the field (PMID: 26266977; PMID: 27864321). Consistent with observations in other cell lines, Parkin overexpression dramatically enhanced O/A-dependent degradation of MTCO2 compared to control cells (**new Figures 5a and 5b**). To further test whether mitochondrial clearance depends on cholesterol, we examined O/A-dependent MTCO2 degradation in the presence of mevastatin to deplete cholesterol. Mevastatin treatment resulted in reduced MTCO2 degradation (**new Figures 5a and 5b**), providing additional evidence that mitochondrial clearance relies on cholesterol enrichment on degradative lysosomal membranes. We now replaced our previous data based on Magic Red with this new data to support the functional evidence of the role of cholesterol in mitochondrial degradation.

We have added the following sentences in the Results section to describe the new data:

> *"We first asked whether efficient degradation of depolarized mitochondria requires cholesterol. To address this, we generated N/TERT cells stably expressing BFP-tagged Parkin and examined mitophagy-dependent degradation of mitochondrial depolarization-dependent degradation of MTCO2 (COXII) by immunoblotting lysates from cells treated with O/A for 2 hours, followed by a 24-hour recovery, in the absence or presence of mevastatin to deplete cholesterol. In wild-type control cells, MTCO2 remained largely detectable, whereas BFP-Parkin-expressing cells showed robust MTCO2 degradation, consistent with previous reports[44,58] (Figure 5a). Notably, MTCO2 clearance was significantly reduced in cells treated with mevastatin, indicating that cholesterol is required for efficient degradation of damaged mitochondria (Figures 5a and 5b)."*

Notably, in the lower left cell of panel 1c, there appears to be no clear EGFP-GRAM-W signal despite strong Tom20 staining. This discrepancy should be addressed.

Reply: Thank you very much for the comment. Although mitochondria are depolarized, not all of them are delivered to degradative lysosomes within 2 hours. Instead, this process occurs gradually over an extended period, with cell-to-cell variation. The Tom20-mScarlet fluorescent signal referenced in the lower left cell of **Figure 1c** corresponds to damaged mitochondria that remain in the cytosol and have not yet been delivered to degradative lysosomes.

6. Figure 1d- Please report the percentage of LAMP1-positive lysosomes that contain engulfed mitochondria to support the claim of lysosomal degradation of mitochondria.

Reply: Thank you very much for the suggestion. We quantified the number of lysosomes labelled with LAMP1 in each cell and determined the proportion containing degrading mitochondria, marked by Tom20-mScarlet (**new Table S1**). Quantification of 18 cells across three independent experiments identified a total of 1,831 lysosomes, of which 1,028 lysosomes contained Tom20-mScarlet, supporting that mitochondria is delivered to lysosomes for degradation.

We next determined the proportion of lysosomes decorated by EGFP-GRAM-W and assessed how many of these GRAM-W positive lysosomes contained Tom20-mScarlet. The distribution of the 1,831 lysosomes is summarized in the table below.

	GRAM-W positive	GRAM-W negative	Total
Tom20 positive	743 (40.58%)	285 (15.57%)	1028 (56.14%)
Tom20 negative	521 (28.45%)	282 (15.40%)	803 (43.86%)
Total	1264 (69.03%)	567 (30.97%)	1831 (100%)

As shown in the table, Tom20-positive structures were more frequently observed in GRAM-W-positive lysosomes (743 events) than in GRAM-W-negative lysosomes (285 events). Statistical analysis using a chi-square test revealed a significant association between Tom20 signal and GRAM-W status ($\chi^2(1) = 11.5$, $p = 0.0007$), with an odds ratio of 1.41 (95% CI: 1.15-1.72). These data are consistent with a modest enrichment of Tom20-positive structures in GRAM-W-positive lysosomes.

Collectively, these results provide additional evidence for the delivery of damaged mitochondria into lysosomes and further support that lysosomes containing damaged mitochondria are more frequently associated with elevated membrane cholesterol.

We have added the following sentences in the Results section to describe the new data:

> *"Quantification of 18 cells across three independent experiments identified a total of 1,831 lysosomes, among which 56% of lysosomes contained Tom20-mScarlet (Supplementary Table 1). We also determined the proportion of lysosomes decorated by EGFP-GRAM-W and assessed how many of these GRAM-W positive lysosomes contained Tom20-mSc. Among the Tom20-positive lysosomes, 72% of those lysosomes were GRAM-W positive. These results provide additional evidence for the delivery of damaged mitochondria into lysosomes and further support that lysosomes containing damaged mitochondria are more frequently associated with elevated membrane cholesterol."*

7. Figure 1f- The interpretation here appears overextended. Please apply stereological analysis to quantify CLEM images in an unbiased and reproducible manner.

Reply: Thank you very much for the comments. We have toned down the relevant statement in our revised manuscript (page 6):

Original sentences:

> *"Finally, correlative light electron microscopy (CLEM) was performed to observe the ultrastructure of EGFP-GRAM-W-positive structures that contained Tom20-mSc*

(Figure 1f). These structures wrapped around elements reminiscent of membranes undergoing digestion, further supporting their identity as degradative lysosomes."

Revised sentences:

"Finally, correlative light electron microscopy (CLEM) was performed to examine the ultrastructure of vesicles that were positive for both EGFP-GRAM-W and LysoTracker^{TM} Deep Red and that contained Tom20-mSc (Figure 1f). These vesicles contained electron dense elements, further supporting their identity as degradative lysosomes."

As described above in new sentences, we included LysoTracker Deep Red (Cyan) to identify degradative lysosomes that were positive for cholesterol (EGFP-GRAM-W: Green) and mitochondria (Tom20-mSc: Magenta). Accordingly, we have replaced **Figure 1f** with a new CLEM image. While we appreciate the importance of quantitative analysis, despite our best efforts, it was challenging to apply stereological analysis to quantify the CLEM images. To support the reproducibility of our observations, we have included additional CLEM images for the reviewer below (**Appendix Figure 1-3**)

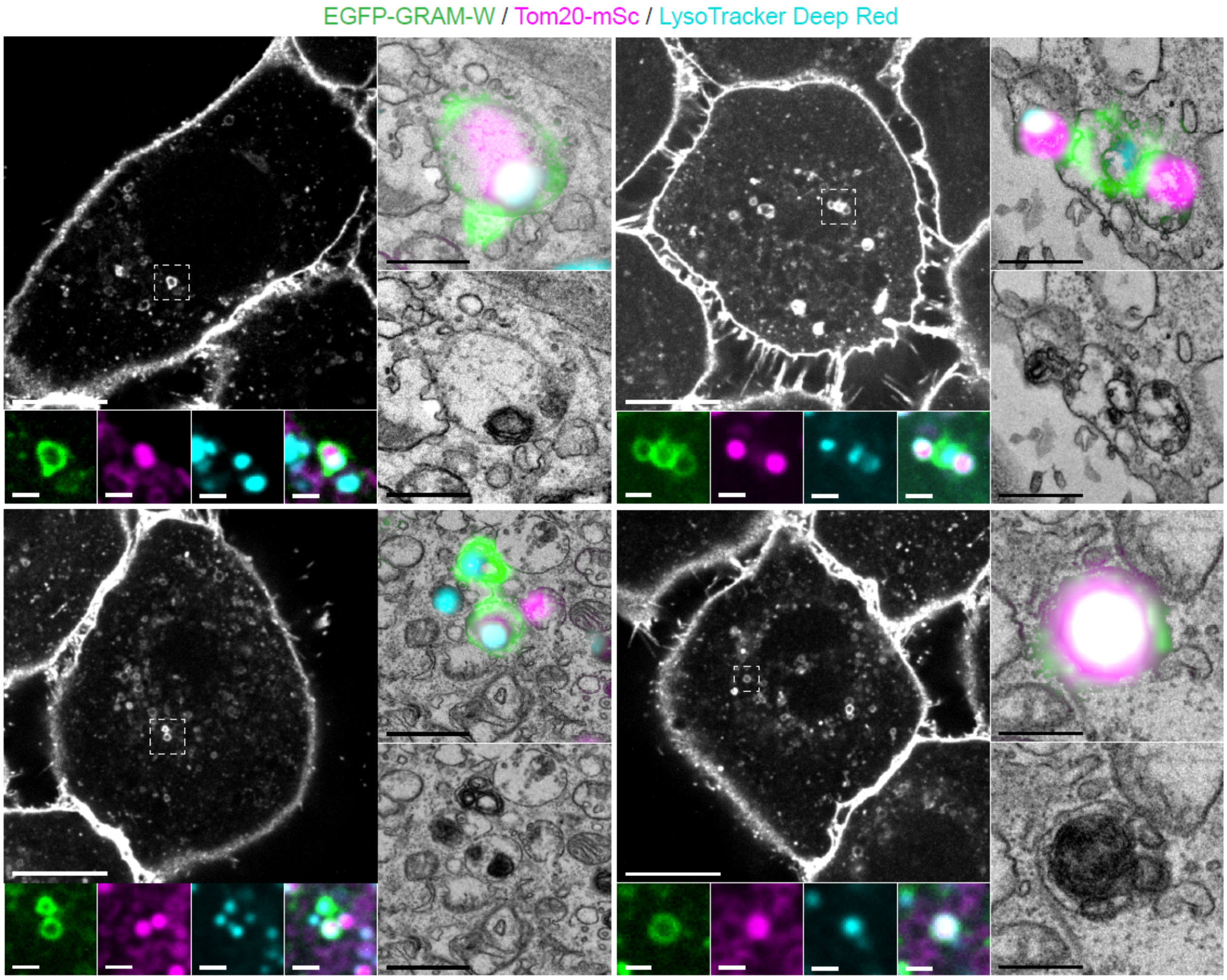

Appendix Figure 1-3
SDC-SIM images and correlated SDC-SIM and transmission electron microscopy images of fixed N/TERT cells expressing EGFP-GRAM-W and TOM20-mSc that were treated with O/A for 2 hours and stained with LysoTracker Deep Red. Scale bars, 10 µm. Insets show at higher magnification the regions indicated by white dashed boxes. Inset scale bars, 1 µm. Black scale bars, 1 µm.

8. The statement in figure legends that "data are pooled from one experiment" is unclear and concerning. If this means that an experiment was performed only once, the findings may be overinterpreted. Please clarify this and ensure that all key conclusions are based on biological replicates from several independent experiments.

Reply: Thank you very much for these important comments and suggestions. In response, we conducted an extensive replication study to increase the sample size and have provided additional information regarding the biological replicates from independent experiments. We have made the following updates:

Figure Number	Original Manuscript	Revised Manuscript
Figure 1b	n = 22-31 cells for each condition; data are pooled from one experiment	n = 66-72 cells for each condition; data are pooled from three independent experiments
Figure 1h	n = 25 cells for control and 23 for O/A; data are pooled from one experiment	n = 86 cells for control and 88 for O/A; data are pooled from three independent experiments
Figure 2b	n = 18-19 for each condition; data are pooled from one experiment	n = 79-83 for each condition; data are pooled from three independent experiments
Figure 2f (previously 2d)	n = 20-24 cells for each condition; data are pooled from one experiment	n = 61-73 cells for each condition; data are pooled from three independent experiments
Figure 2i (previously 2g)	n = 27-37 cells for each condition; data are pooled from one experiment	n = 62-66 cells for each condition; data are pooled from three independent experiments
Figure 2k (previously 2i)	n = 17-22 for each condition; data are pooled from one experiment	n = 99 for each condition; data are pooled from three independent experiments
Figure 3h	n = 24 cells for both conditions; data are pooled from one experiment	n = 92 cells for both conditions; data are pooled from four independent experiments
Figure 5d	n = 26-30 cells for each condition; data are pooled from one experiment	n = 63 cells for each condition; data are pooled from three independent experiments
Figure 5f	n = 35-45 cells for each condition; data are pooled from one experiment	n = 80-83 cells for each condition; data are pooled from three independent experiments
Figure 6i	n = 51 cells for control and 31 cells for O/A; data are pooled from one experiment	n = 82 cells for control and 83 cells for O/A; data are pooled from three independent experiments
Figure 6l	n = 16-26 cells for each condition; data are pooled from one experiment	n = 69-81 cells for each condition; data are pooled from three independent experiments
Figure 7b	n = 28 cells for DMSO + O/A and 24 for BafA1 + O/A; data are pooled from one experiment	n = 83 cells for DMSO + O/A and 84 for BafA1 + O/A; data are pooled from three independent experiments
Figure 7d	n = 20-23 cells for each condition; data are pooled from one experiment	n = 88-91 cells for each condition; data are pooled from three independent experiments
Figure 7f	n = 27-29 cells for each condition; data are pooled from one experiment	n = 82-85 cells for each condition; data are pooled from three independent experiments

New Figure 7h	NA	n = 59 cells for WT + O/A and 54 for PI4KIIα KO + O/A; data are pooled from three independent experiments
New Figure 7j	NA	n = 69-75 cells for each condition; data are pooled from three independent experiments
Figure S7b	n = 26-38 cells for each condition; data are pooled from one experiment	n = 60 cells for each condition; data are pooled from three independent experiments
Figure S7d	n = 21-26 cells for each condition; data are pooled from one experiment	n = 56-71 cells for each condition; data are pooled from three independent experiments
New Figure S7h	NA	n = 92-96 cells for each condition; data are pooled from three independent experiments

We also updated the Methods section to provide additional details on the quantification methods.

9. Since the manuscript discusses temporal regulation of lipid signaling and organelle interactions, live-cell imaging using multiple markers (e.g., for PI4P, lysosomes, mitochondria) is necessary. For example, in Fig. S2b, EGFP-GRAM-W signal appears reduced after 20 min of O/A treatment. Please quantify the number and intensity of vesicles over time and relate this to lysosome–mitochondria dynamics.

Reply: Thank you very much for this suggestion. We have performed additional time-lapse imaging of cells expressing EGFP-GRAM-W (cholesterol marker), dTomato-ORP9-PH (PI4P marker), and LAMP1-miRFP703 (lysosomal marker) that were treated with O/A and quantified the levels of lysosomal cholesterol and PI4P over time. We have replaced the images and added quantification in **new Figures 2c and 2d**.

10. Figure 2a- PI4P appears recruited in PI4K2A KO cells upon O/A treatment. Please explain this observation. What is the source of PI4P if PI4K2A is reported to be the sole contributor?

Reply: Thank you very much for this comment. While PI4KIIα seems to be the major lipid kinase to produce PI4P on lysosomes upon O/A treatment, we believe that other lipid kinases or machineries, such as PI4KIIIβ (PMID: 37289133; PMID: 23258225; PMID: 37883971), may also produce PI4P on lysosomes. This is also in line with our new data showing much reduced, but not completely eliminated, recruitment of OSBP to lysosomes upon O/A treatment in PI4KIIα KO cells (**new Figures S2f and S2g**) (please refer to reply to comment #2 above for experimental conditions). We had also noted that PI4P accumulation in lysosomes was not completely eliminated in PI4KIIα KO cells (**Figure 2b**).

We have added the following sentences in the Discussion section to describe the new data:

> *"We found that the knockout of PI4KIIα significantly, though not completely, suppressed the increase of PI4P on degradative lysosomal membranes during mitophagy (Figure 2a and 2b). While our results confirm that PI4KIIα is the major kinase for PI4P synthesis in this process, we do not exclude the possibility that other enzymes, such as PI4KIIIβ[19,77,78], may also contribute to the increase of PI4P on lysosomal membranes."*

11. Please evaluate lysosomal pH over time after O/A treatment and not just recovery using the pH-sensitive probe in live cells. This is essential to support claims regarding lysosomal functional integrity.

Reply: Thank you very much for this suggestion. We would like to clarify that mitophagy continues after O/A treatment, as demonstrated by our analysis of cells expressing a mitophagy reporter (**Figures S7c and S7d**) and by our analysis of cells treated with O/A followed by a 24-hour recovery, which displayed enlarged lysosomes, indicative of ongoing lysosome-autophagosome fusion (**Figures 1g and 1h**). To avoid confusion, we revised the following sentences in our revised manuscript:

Original sentences:

> *"To investigate whether the accumulation of cholesterol on degradative lysosomes is transient or sustained, cells were treated with O/A for 2 hours, allowed to recover for 24 hours, and then immunostained for LAMP1. Degradative lysosomes were larger, as revealed by LAMP1 staining, after mitophagy induction (Figures 1g and 1h). Importantly, cholesterol remained highly enriched on these degradative lysosomal membranes."*

Revised sentences:

> *"To investigate whether the accumulation of cholesterol on degradative lysosomes is transient or sustained, cells were treated with O/A for 2 hours, allowed to recover for 24 hours, and then immunostained for LAMP1. Following mitophagy induction, degradative lysosomes were larger, as revealed by LAMP1 staining (Figures 1g and 1h), indicating that mitophagy continues during this period with ongoing lysosome-autophagosome fusion. Importantly, cholesterol remained highly enriched on these degradative lysosomal membranes, suggesting sustained accumulation throughout the mitophagy process."*

We have also performed additional imaging experiments using FIRE-pHLy to monitor lysosomal pH over time following O/A treatment. As shown in **Appendix Figure 1-4**, lysosomes are able to maintain their pH for a certain period after O/A treatment. However, at 16 hours post-treatment, we observed a significant increase in lysosomal pH, indicating a loss of lysosomal acidification and a collapse in pH maintenance. These results suggest that lysosomal integrity is robustly maintained during the early phase of mitophagy but becomes compromised as mitophagy progresses following mitochondrial depolarization.

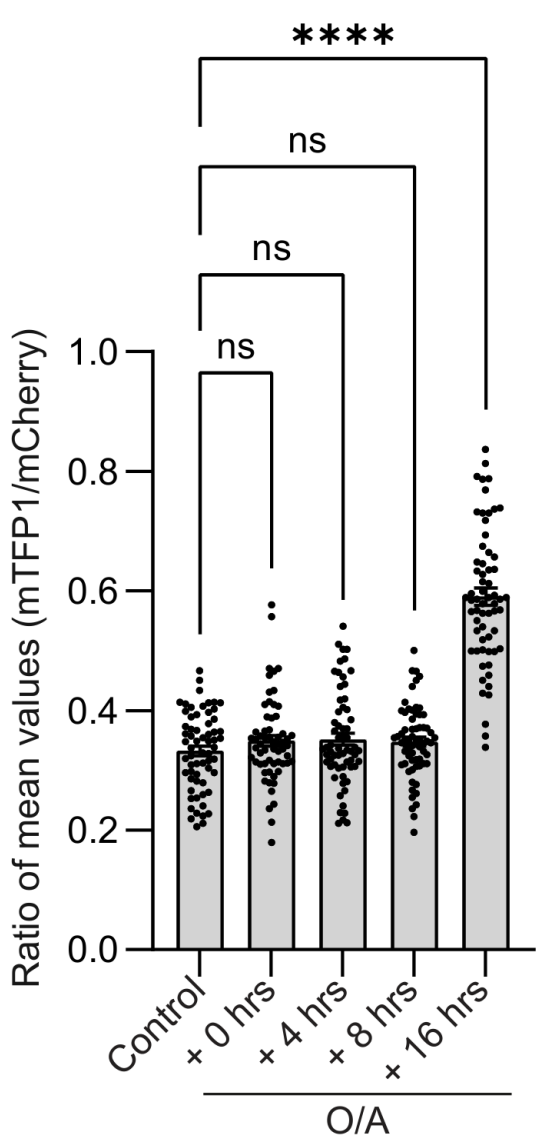

Appendix Figure 1-4
Quantification of the ratio of mean fluorescent intensity of mTFP1 to that of mCherry in each cell. Cells were not treated (Control) or treated with O/A for 2 hours, followed by recovery for times as indicate. (mean ± SEM, n = 61-65 cells for each condition; data are pooled from three independent experiments; Dunnett's multiple comparisons test, ****$P < 0.0001$).

12. GAL3 is a well-established marker of large-scale lysosomal rupture, but it may miss subtle membrane damage. To capture early and small-scale damage, please include CHMP4B localization as an ESCRT-III marker with faster recruitment kinetics (see Skowyra et al., Science, 2018). This additional analysis would clarify whether the lysosomes are truly undamaged or undergoing sub-lethal repair.

Reply: Thank you very much for these suggestions. We encountered technical difficulties in generating N/TERT cells stably expressing EGFP-tagged CHMP4B. As an alternative approach, we performed additional imaging experiments using antibodies against CHMP2A, another ESCRT-III component that is recruited to damaged lysosomes together with CHMP4B (PMID: 37968398). Cells were treated with O/A for 2 hours or LLOMe for 1 hour, followed by fixation and immunostaining for CHMP2A and LAMP1. LLOMe treatment induced robust accumulation of CHMP2A to lysosomes, consistent with previous reports. In contrast, O/A treatment did not induce CHMP2A accumulation at lysosomes, consistent with the absence of lysosomal membrane damage (**new Figure S1f**). These results further confirm that lysosomal damage is not induced during mitophagy.

We have revised the following statement in the Results section to describe the new data:

Original sentences:

> *"However, O/A treatment did not induce the rupture of lysosomal membranes, as assessed in fixed cells via immunostaining for galectin-3 (Gal-3) (Figure S1e). In contrast, treatment with L-Leucyl-L-Leucine methyl ester (LLOMe), a compound known to disrupt lysosomal membranes, resulted in clear Gal-3 recruitment (Figure S1e). These results rule out membrane disruption as the cause of cholesterol accumulation during mitophagy."*

Revised sentences:

> *"However, O/A treatment did not induce the rupture of lysosomal membranes, as assessed in fixed cells via immunostaining for galectin-3 (Gal-3) and for CHMP2A[49,50] (Figures S1e and S1f). In contrast, treatment with L-Leucyl-L-Leucine methyl ester (LLOMe), a compound known to disrupt lysosomal membranes, resulted in clear Gal-3 and CHMP2A recruitment (Figures S1e and S1f). These results rule out membrane disruption as the cause of cholesterol accumulation during mitophagy."*

13. Fig.5- Authors report that lysosomal pH increases after O/A treatment (+24 h recovery). However, LLOMe requires acidic pH for activation. This raises the possibility that low levels of lysosomal damage in O/A + LLOMe conditions may result from impaired LLOMe activity due to elevated pH. Please address this potential confounding factor.

Reply: Thank you very much for pointing this out. Indeed, our experiments suggest that lysosomal pH increases upon O/A treatment, which may have led to an underestimation of the extent of damage caused by LLOMe treatment.

We have included the following sentences in the Results section to acknowledge this possibility:

> *"Because LLOMe requires an acidic environment for enzymatic activation, elevated lysosomal pH, as shown earlier in O/A or mevastatin-treated cells, could reduce the potency of LLOMe to induce membrane damage. However, despite this potentially*

> *inhibitory pH shift, N/TERT cells treated with O/A or mevastatin showed a dramatic enhancement in lysosomal membrane rupture upon LLOMe exposure."*

14. Given the importance of ER–lysosome contact sites and OSBP-mediated lipid transfer, it is essential to examine how ER morphology and dynamics respond to O/A treatment. Please include live-cell imaging of ER structure over time following O/A exposure.

Reply: Thank you very much for the suggestion. Following the reviewer's suggestion, we generated N/TERT cells stably expressing an ER marker, BFP-tagged Sec61β, and imaged ER morphology following exposure to O/A. We found that the ER network was disrupted, lacking the clear sheets as typically observed in untreated control cells (**new Figure S2h**). Notably, the ER and EGFP-GRAM-W positive vesicles were in close association in O/A-treated cells (**new Figure S2h**), supporting the presence of close contacts between the ER and lysosomes under this condition.

We have added the following sentences in the Results section to describe the new data:

> *"Finally, we generated N/TERT cells stably expressing an ER marker, BFP-tagged Sec61β, and imaged ER morphology following exposure to O/A. In O/A-treated cells, the ER network was disrupted with the absence of clear sheets as typically observed in untreated control cells (Figure S2h). Notably, the ER and EGFP-GRAM-W-positive vesicles were in close association in O/A-treated cells (Figure S2h), supporting the presence of close contact between the ER and lysosomes under this condition."*

Minor points:

1. Figure 1- At later points following O/A treatment, particularly after 2h and 4h washout, cholesterol appears to accumulate at the plasma membrane. Please clarify this observation and discuss its potential relevance.

Reply: Thank you very much for the comments. Indeed, we observed accumulation of cholesterol at the plasma membrane, as assessed by EGFP-GRAM-W, upon O/A treatment. Interestingly, inhibition of OSBP via OSW-1 did not prevent this cholesterol accumulation (**Figure 2f**). This suggests that such cholesterol accumulation may be mediated by OSBP-independent mechanisms. While the characterization of such mechanisms is beyond the scope of the current study, we now discuss the potential relevance of this finding in the Discussion section as follows:

> *"In addition to the increase of cholesterol on lysosomal membranes, we also observed some increase of cholesterol at the PM, as assessed by EGFP-GRAM-W, following O/A treatment (Figure 1a). Notably, this increase in PM cholesterol was not suppressed by OSBP inhibition (Figure 2h), suggesting that such cholesterol accumulation at the PM is mediated by OSBP-independent mechanisms. This process may also contribute to the depletion of ER cholesterol and the subsequent activation of SREBP-2. Future studies will be required to elucidate the molecular mechanisms underlying this process."*

2. Figure 1b- Quantify the number of vesicles per cell to provide a clearer picture of vesicular dynamics following O/A treatment.

Reply: Thank you very much for the suggestion. We have quantified the number of EGFP-GRAM-W-positive vesicles and updated the quantification accordingly.

3. Figure 2e- Include quantitative colocalization analysis between PI4P and lysosomal markers to support your interpretations.

Reply: Thank you very much for the suggestions. We would like to clarify that in the **Figure 2e** of the original manuscript **(now Figure 2g in the revised manuscript)**, OSBP and the lysosomal marker LAMP1 were measured by immunofluorescence. The quantification of OSBP colocalization with LAMP1 is provided in **Figure 2i** of the original manuscript **(now Figure 2k in the revised manuscript)**. Although we observed some OSBP signals in proximity to LAMP1-positive lysosomes, the quantification revealed no statistically significant increase in OSBP recruitment.

To improve the accuracy and clarity of our claims, we have revised the following statement in the Results section:

Original sentences:

> *"Compared to control cells, in which OSBP primarily localized to the TGN (Figure S2f), OSBP was distributed throughout the cytoplasm in cells treated with O/A, with some accumulation in degradative lysosomes (Figure 2e)."*

Revised sentences:

> *"Compared to control cells, in which OSBP primarily localized to the TGN (Figure S2e), OSBP was re-distributed throughout the cytoplasm in cells treated with O/A (Figure 2g)."*

Reviewer #2 (Remarks to the Author):

In their manuscript, Yang and co-workers describe an interesting new mechanism in which cholesterol build-up in lysosomal membranes enables the upkeep of their degradative capacity and membrane integrity during mitochondrial degradation. Their data point to an initial PI4P production that activates OSBP for cholesterol to be transported from the ER to the lysosomes, causing the ER to get depleted from cholesterol. This, in turn, activates SREBP2-linked de novo cholesterol synthesis. When lysosomes function well, the mitochondrial lipids are effectively broken down in fatty acids which they show are converted to TAG for storage in lipid droplets. While some mechanistic aspects of the paper have been described before, the authors succeeded in connecting/dissecting in much mechanistic detail to explain how lysosomes maintain their integrity/function while 'consuming' an overload of mitochondrial membranes. I am not aware of any direct prior work that would have shown a similar complete proposed axis related to mitochondrial degradation. Overall the technical and experimental quality of the paper is high and very well controlled by independent and/or orthogonal approaches making it a convincing story suitable for publication. I have only some limited remarks and suggestions that the authors must address.

We greatly appreciate the reviewer's positive comments on our manuscript. Thank you very much for all the constructive comments to further improve our manuscript.

Major remarks:

- The authors performed some TEM analysis of the cells (Fig. 1f, 6j). However, none of these are showing or connecting to the lipid droplet (LD) phenotype, being the proposed end-stage of the mitochondrial lipid content by the authors. In this regard, different studies have detected that the accumulation of cholesterol might be linked to dysfunctional lysosomes in neurodegenerative diseases and described the formation of multilamellar bodies (MLB) instead, also specifically linked to mitochondrial/mitophagy defects (PMID: 29368986; PMID: 15840653, PMID: 37225734). Would there be a threshold of beneficial and detrimental cholesterol accumulation, leading to either LD or MLB? Can the authors discuss or investigate?

Reply: Thank you very much for these comments and suggestions. The outcome may depend on the subcellular location of cholesterol accumulation rather than a simple threshold. This distinction may determine whether cholesterol leads to mitophagy defects and multilamellar body (MLB) formation, or instead, supports efficient mitophagy and lipid droplet (LD) formation.

In the studies cited by the reviewer, autophagy defects and/or MLB accumulation are likely associated with cholesterol accumulation within the lysosomal lumen, due to a mutation in GBA1 (PMID: 29368986; PMID: 28779532), overexpression of Mgat5 coupled with NPC1 inhibition (PMID: 15840653), or the lack of PLD3 (PMID: 37225734). Such abnormal luminal cholesterol accumulation, which is detrimental, is often linked to impaired lysosomal degradative capacity and defective mitophagy, as also shown recently in NPC1-deficient cells (e.g., PMID: 33308480).

In contrast, in normal cells, where luminal cholesterol does not exceed a pathological threshold, cholesterol accumulation at the lysosomal limiting membrane appears to be beneficial. It helps maintain lysosomal structure and degradative function during mitophagy and supports efficient LD formation, consistent with our findings in the current study. We appreciate the reviewer's insightful comments and have incorporated new sentences reflecting this point into the Discussion section of the revised manuscript.

> *"Our findings suggest that accumulation of cholesterol on lysosomal membrane helps maintain lysosomal function and integrity. We find that this pool of cholesterol supports efficient degradative flux and lipid storage into LDs. In contrast, the accumulation of*

cholesterol within the lysosomal lumen has been reported in pathological states. For example, NPC1 deficiency, which leads to luminal cholesterol accumulation, impairs lysosomal integrity, degradative capacity, and mitophagy[68]. Moreover, luminal cholesterol accumulation is frequently associated with lysosomal dysfunction and the formation of multilamellar bodies (MLBs), which can be induced by mutation in GBA1[70,71], overexpression of Mgat5 coupled with NPC1 inhibition[72], or the loss of PLD3[73]. Together, these observations suggest that the site of cholesterol accumulation, whether at the limiting membranes or within the lumen, has profound functional implications for lysosomal homeostasis."

- Of note in this regard: "Fig. 6j. Transmission electron microscopy images of N/TERT cells with indicated treatment. Yellow arrowheads indicate lipid droplets. N, nucleus. PM, plasma membrane. Scale bars, 1 µm." To my opinion the arrowheads rather indicate degradative mature lysosomes (typically very dark to black content at the EM level), not lipid droplets. Lipid droplets typically appear as white to fuzzy light-grey organelles with a lipid monolayer on transmission electron microscopy (TEM), not as electron-dense vesicles as indicated here. Different examples can be found in literature, e.g. PMID: 31959936, showing both lipid droplets and lysosomes. Could the authors provide broader images of the cells as currently the case and use insets to highlight clear/correctly identified lipid droplets, if present? There is also no dedicated description on how the TEM was done to visualize LDs in the M&M section (only CLEM is described). Please include more details on the EM processing.

Reply: Thank you very much for the constructive comments. We reviewed the literature and found that, in most cases, lipid droplets (LDs) appear light grey rather than electron-dense; however, there are some reports of dense LDs (e.g., PMID: 21430261). To address this concern, we performed new CLEM using N/TERT cells stably expressing the LD marker mCherry-ADRP. Cells were either treated with ethanol (Control) or O/A for 2 hours and recovered for 24 hours before processing for CLEM. Our new data showed clear colocalization between mCherry-ADRP and the electron-dense structures, confirming their identity as LDs. In the revised manuscript, we have replaced the old images in **Figure 6j** with these new CLEM images and included broader views with insets to show the LDs at higher magnification (**new Figure 6j**).

- Fig. 5C. It was recently shown that calcium dysregulations would be upstream of acidification defects (DOI/10.1083/jcb.201201076DOI: 10.7554/eLife.15887, DOI: 10.1016/j.devcel.2024.03.030, DOI: 10.1073/pnas.2211999119). Changes in the levels of calcium and disruptions in the calcium-gradient can hinder the acidity of lysosomes by reducing the force that drives the influx of hydrogen ions, thus preventing the restoration of lysosomal function. (DOI: 10.3390/cells9122655). Some papers further detect calcium defects without pH changes e.g. in Niemann-Pick disease type C1 (DOI: 10.1038/nm.1876). Given the importance of calcium to the ER (and lysosome) and the link to cholesterol, could the authors investigate to what extent lysosomal Calcium is affected and could contribute to the observed defects. Currently the mechanism is limited to a potential role of cholesterol in the assembly of V-ATPase.

Reply: Thank you very much for these detailed comments and suggestions. We performed new time-lapse imaging experiments using Fluo-4 AM and glycyl-L-phenylalanine 2-naphthylamide (GPN), a compound that induces lysosomal permeabilization, to assess lysosomal Ca^{2+} levels by measuring the transient increase in cytosolic Ca^{2+} upon lysosomal permeabilization. Cells were pretreated with DMSO or 5 µM mevastatin for 24 hours, followed by treatment with ethanol or O/A for 2 hours, and then allowed to recover for 24 hours in the continued presence of DMSO or mevastatin (the same four conditions as in Figure 5c). Prior to imaging, cells were loaded with Fluo-4 AM for 1 hour at room temperature and subsequently incubated at 37 °C for 30 min. Time-lapse images were acquired at 1-min intervals, and GPN

was added at the 10-min time point. Cytosolic fluorescence intensity was quantified and normalized to the mean intensity of the first 10 frames.

The peak Fluo-4 fluorescence intensity was comparable across all conditions, although some variation in the post-peak Ca^{2+} dynamics was observed (**Appendix Figures 2-1a and 2-1b**). While we cannot exclude potential changes in lysosomal Ca^{2+} levels, the current experiments do not allow us to draw a definitive conclusion regarding the effects of O/A or mevastatin treatment on lysosomal Ca^{2+} maintenance. We thank the reviewer for this insightful suggestion, and we have discussed the potential role of Ca^{2+} in maintaining the degradative capacity of lysosomes in the revised manuscript.

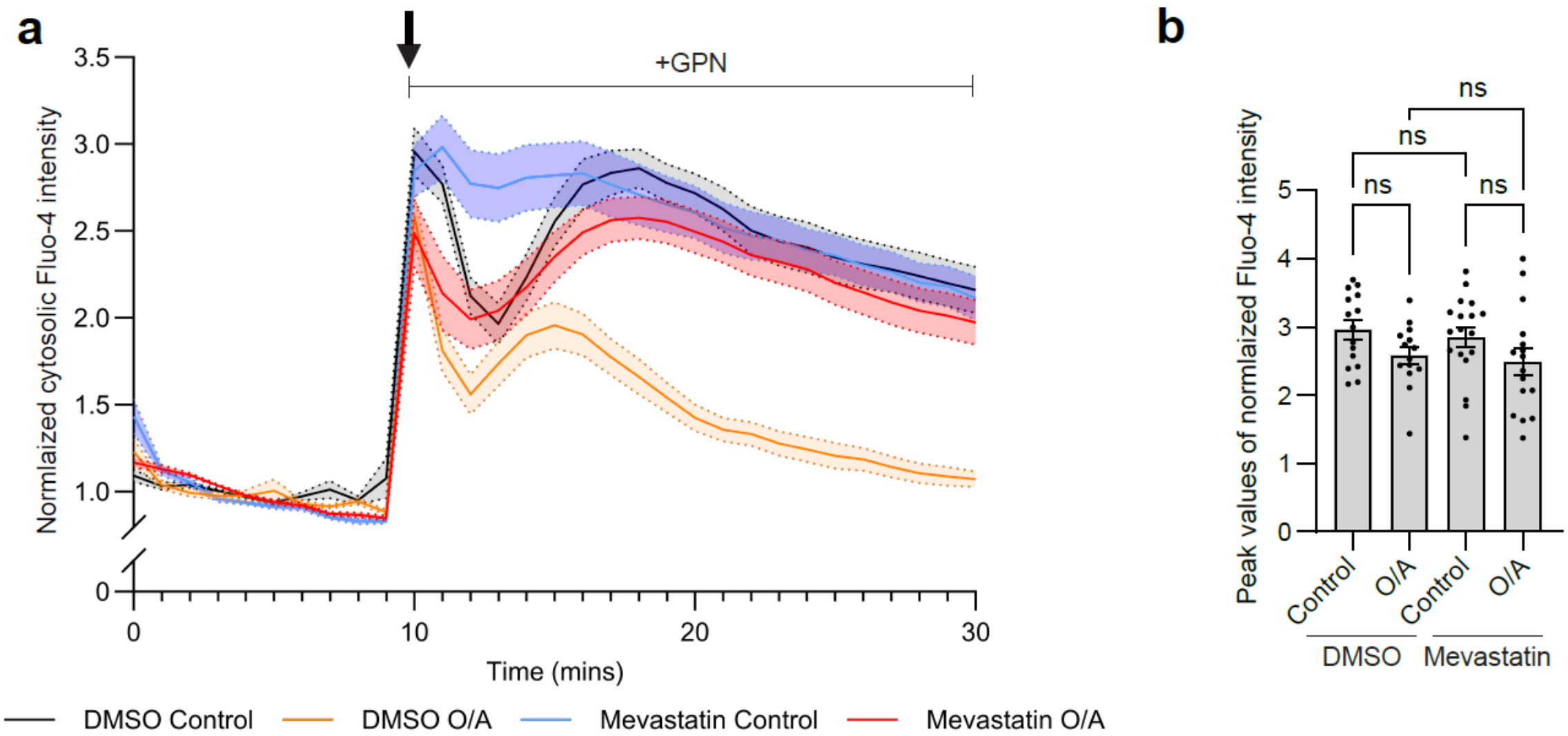

Appendix Figure 2-1
a. Normalized cytosolic Fluo-4 fluorescence intensity over time in cells treated with conditions as indicated. N/TERT Cells were treated with DMSO or 5 μM mevastatin for 24 hours before treatment with ethanol or O/A for 2 hours, washed with PBS, and recovered in KFSM for 24 hours in the presence of DMSO or mevastatin. GPN was added at 10 minutes time point. **b.** Peak values of normalized cytosolic Fluo-4 intensity (mean ± SEM, n = 14-18 for each condition; data are pooled from two independent experiments; Tukey's multiple comparison test, ns, not significant).

We have added the following new sentences into the Discussion section of the revised manuscript:

> *"Another factor important for maintaining the degradative capacity of lysosomes is lysosomal Ca^{2+} homeostasis. Growing evidence suggests that Ca^{2+} dysregulation can impair endolysosomal function[81-84]. Disruption of the Ca^{2+} gradient across lysosomal membranes may alter the electrochemical balance required for proton influx, thereby reducing the efficiency of lysosomal acidification[85]. However, defective lysosomal Ca^{2+} homeostasis has also been reported to occur in the absence of lysosomal pH defects[86]. Whether disruptions in cholesterol homeostasis affect lysosomal Ca^{2+} regulation remains an important question for future investigation."*

- The discussion section primarily includes a list of five major conclusions, which are then repeated in the next paragraphs. This results in repetition and a lack of integration with the existing literature; for example, the final paragraph on page 16 (refs. 71-85) mentions multiple research articles without completely contextualizing them. The discussion might benefit from less repetition and a more thorough integration of the findings into the larger scientific context. For instance, several aspects of this mechanistic cascade may fail (for different reasons) in the context of neurodegenerative diseases, including Alzheimer's and Parkinson (lysosomal dyshomeostasis, mitochondrial dysfunction, lipid metabolism dysregulations), and wherein

lysosomal abnormalities are increasingly taking in a central position (also based on genetics with many risk genes related to endolysosomal/autophagy functions in AD, PD as well as ALS and spastic paraglegia (SP)). Several risk/causal proteins are also linked to ER-Lys MCSs, such as VAPB (ALS), protrudin (SP), VPS13C (PD/DLB; doi.org/10.1186/s40478-021-01121-w; doi.org/10.1016/j.ajhg.2016.01.014) and recently APP (doi.org/10.1016/j.devcel.2024.03.030) suggesting failing MCSs could be a hub for initiating lysosomal dyshomeostasis (reviewed recently in DOI: 10.1002/bies.202400023).

Reply: Thank you very much for these comments and suggestions. While we retain the five major conclusions of our study, we have minimized repetition wherever possible. We appreciate the reviewer for introducing these insightful viewpoints.

We have revised the following sentences in the Discussion section (original refs. 71-85):

Original sentences:

> *"Impaired clearance of damaged mitochondria, dysregulated cholesterol homeostasis, lysosomal dysfunction, and defective LD formation have all been linked to neurodegenerative disorders[3,71-73]. For example, dysfunction of PINK1 and Parkin, key regulators of mitophagy, are linked to Parkinson's disease (PD)[74-76]. Additionally, dysfunction of lysosomal proteins, such as GBA1, LRRK2, and TMEM175, have been associated with PD[73,77,78]. Likewise, dysregulated cholesterol homeostasis and abnormal LD formation have been linked to neurodegenerative disorders, including PD and Alzheimer's disease (AD)[72,79-85]. Our findings reveal cholesterol as a central regulator of lysosomal functional integrity and LD formation during clearance and recycling of dysfunctional mitochondria, offering novel insights into cellular mechanisms that enable adaptation to organelle dysfunction."*

Revised sentences:

> *"The pathogenesis of neurodegenerative disorders is increasingly associated with the breakdown of inter-organelle communication[90]. Our findings demonstrate the critical importance of coordinated lipid transfer reactions at ER-lysosome contact sites in maintaining the degradative capacity of lysosomes for proper clearance of damaged mitochondria and subsequent recycling of their lipids into LDs. Failure of this process could potentially underlie neurodegeneration as impaired clearance of damaged mitochondria, lysosomal dysfunction, and defective LD formation have all been linked to various neurodegenerative disorders[3,91-93]. For example, dysfunction of key regulators of mitophagy, including PINK1 and Parkin, are tightly linked to Parkinson's disease (PD)[94-96]. Growing evidence suggests that lysosomal abnormalities have been associated with Alzheimer's disease (AD)[97], PD[93,98-100], amyotrophic lateral sclerosis (ALS)[99], and hereditary spastic paraplegia (HSP)[101]. Likewise, dysregulated cholesterol homeostasis and abnormal LD formation have been linked to PD and AD[92,102-108]. Finally, the dysfunction of the proteins involved in lipid transfer and / or the maintenance of ER-lysosome contact sites, including VPS13C, VAPB, Protrudin, are implicated in several neurodegenerative disorders, including ALS[109-111], HSP[112,113], and PD[79,114-116]. Interestingly, accumulation of amyloid precursor protein (APP), a primary driver of plaque formation in AD brain, has been reported to disrupt ER-late endosome/lysosome contact sites, suggesting that the instability of these contact sites may be an early event in AD[83]. Collectively, these findings underscore the vital role of inter-organelle communication at membrane contact sites in the maintenance of brain health."*

We have also removed the following sentences from the Discussion to avoid repetition:

“Our study identifies OSBP as a central regulator of cholesterol transport and homeostasis during the clearance of dysfunctional mitochondria.”

“We found that PI4KIIα is a key enzyme for PI4P synthesis on degradative lysosomal membranes to activate OSBP-mediated cholesterol delivery.”

“Our study demonstrates that lipids released from the autophagic digestion of mitochondria are the key driver of LD formation. We found that mitochondrial depolarization-induced mitophagy leads to the additional formation of DGAT1-dependent LDs.”

Small remarks:

*- The authors should indicate the specific statistical values instead of working with ****

Reply: Thank you very much for this suggestion. We would like to clarify that all the values of statistical tests were included in the figure legends. The asterisks in the figures were used for visual aesthetics.

- Fig. 1D. Could the authors quantify if lysosomes containing mitochondria have a higher cholesterol/GRAM signal? As this would provide more strength to the causality claim. If not, it could be a downstream effect.

Reply: Thank you very much for the suggestion. We quantified the number of lysosomes labelled with LAMP1 in each cell and determined the proportion containing degrading mitochondria, marked by Tom20-mScarlet. Quantification of 18 cells across three independent experiments identified a total of 1,831 lysosomes, of which 1,028 lysosomes contained Tom20-mScarlet, supporting that mitochondria is delivered to lysosomes for degradation.

We next determined the proportion of lysosomes decorated by EGFP-GRAM-W and assessed how many of these GRAM-W positive lysosomes contained Tom20-mScarlet. The distribution of the 1,831 lysosomes is summarized in the table below.

	GRAM-W positive	GRAM-W negative	Total
Tom20 positive	743 (40.58%)	285 (15.57%)	1028 (56.14%)
Tom20 negative	521 (28.45%)	282 (15.40%)	803 (43.86%)
Total	1264 (69.03%)	567 (30.97%)	1831 (100%)

As shown in the table, Tom20-positive structures were more frequently observed in GRAM-W-positive lysosomes (743 events) than in GRAM-W-negative lysosomes (285 events). Statistical analysis using a chi-square test revealed a significant association between Tom20 signal and GRAM-W status ($\chi^2(1) = 11.5$, $p = 0.0007$), corresponding to an odds ratio of 1.41 (95% CI: 1.15–1.72). These data are consistent with a modest enrichment of Tom20-positive structures in GRAM-W-positive lysosomes.

Collectively, these results provide additional evidence for the delivery of damaged mitochondria into lysosomes and further support that lysosomes containing damaged mitochondria are more frequently associated with elevated membrane cholesterol.

We have added the following sentences in the Results section to describe the new data:

“Quantification of 18 cells across three independent experiments identified a total of 1,831 lysosomes, among which 56% of lysosomes contained Tom20-mScarlet

> *(Supplementary Table 1). We also determined the proportion of lysosomes decorated by EGFP-GRAM-W and assessed how many of these GRAM-W positive lysosomes contained Tom20-mSc. Among the Tom20-positive lysosomes, 72% of those lysosomes were GRAM-W positive. These results provide additional evidence for the delivery of damaged mitochondria into lysosomes and further support that lysosomes containing damaged mitochondria are more frequently associated with elevated membrane cholesterol."*

As with Fig. 2a, please conduct the analysis within each condition individually, rather than limiting the comparison to control versus O/A.

Thank you very much for this suggestion. We added additional comparisons in **Figures 2b, 2f, 2i, 4d, 4f, 4l, 5d, 5f, 6l, 7d, 7f, 7j, S2g, S4f, S7h, and S7j** to show the analyses within each condition, with the corresponding *P* values included in the figure legends.

- Fig. S2b: "Importantly, this PI4P accumulation preceded that of cholesterol, indicating that PI4P may drive the accumulation of cholesterol on degradative lysosomal membranes during mitophagy (Figure S2b).". Can the authors provide zooms to clearly substantiate the claim? At 20, we can already see a green spot right upper quadrant that is first green before magenta, idem for a vesicle left below corner at 40. Maybe this is just less clear without zooms provided.

Reply: Thank you very much for these suggestions. To further support our claim, we performed additional time-lapse imaging experiments using N/TERT cells stably expressing the lysosomal marker LAMP1-miRFP703 together with a PI4P maker (dTomato-ORP9-PH) and a cholesterol marker (EGFP-GRAM-W). We then quantified the accumulation of PI4P and cholesterol on lysosomes following O/A treatment. Following the reviewer's recommendation, we have added zoomed-in images to more clearly show individual vesicles. These new results are now included in **new Figures 2c and 2d** of the revised manuscript.

- Is it possible that figure 6, panels k and l are not discussed/referred to in the Result section?

Reply: Thank you very much for pointing this out. Indeed, there were errors in our text, and Figure 6 k and l were not referenced correctly. We have corrected it.

- Could the authors list which reference genome (version) was used for the mRNA-seq?

Reply: Thank you very much for this suggestion. We used the following reference genome for our mRNA-seq experiments. This information is now added in the Method section.

- Species: Homo sapiens
- Origin: Ensembl
- Version: GRCh38.p13

We have included this information in our Methods section:

> "Obtained data were cleaned and aligned to reference genome (Homo sapiens, Ensembl, GRCh38.p13) via software Hisat2 (v2.0.1)."

- Data availability. The authors should deposit their RNA and lipidomics data to open repositories like MetaboLights or the Metabolomics Workbench.

Reply: Thank you very much for these suggestions. We will deposit these data upon acceptance / publication, in accordance with the journal's data availability policy.

- Could the authors provide information on which internal standards were used (types, quantities) for the lipidomic analysis?

Reply: Thank you very much for this suggestion. We have now included this information in a **new Table S3** in the revised manuscript.

Reviewer #3 (Remarks to the Author):

In this study, Yang and colleagues identified cholesterol as a critical regulator maintaining lysosomal functional integrity during the clearance and recycling of dysfunctional mitochondria. They found that mitochondrial depolarization triggers acute cholesterol delivery from the ER to degradative lysosomal membranes, mediated by PI4P-driven, OSBP-dependent transport. This massive cholesterol efflux from the ER activates SREBP-2, driving cholesterol biosynthesis and ultimately lipid droplet (LD) formation—revealing cholesterol's pivotal role in maintaining cellular homeostasis during organelle stress. The data mostly supports the conclusions and there are a number of additional experiments I think would strengthen the conclusions:

We greatly appreciate the reviewer's positive comments on our manuscript. Thank you very much for all the constructive comments to further improve our manuscript.

Major points:

1. Most quantifications are not properly done: statistics should be performed between independent samples with the sample size to at least 50 cells per group per biological replicate. The experiments should be repeated at lease three times and statistics performed between experiments (using the mean of several cells quantified per experiment).

Reply: Thank you very much for these important comments and suggestions. In response, we conducted an extensive replication study to increase the sample size and have provided additional information regarding the biological replicates from independent experiments. We have made the following updates:

Figure Number	Original Manuscript	Revised Manuscript
Figure 1b	n = 22-31 cells for each condition; data are pooled from one experiment	n = 66-72 cells for each condition; data are pooled from three independent experiments
Figure 1h	n = 25 cells for control and 23 for O/A; data are pooled from one experiment	n = 86 cells for control and 88 for O/A; data are pooled from three independent experiments
Figure 2b	n = 18-19 for each condition; data are pooled from one experiment	n = 79-83 for each condition; data are pooled from three independent experiments
Figure 2f (previously 2d)	n = 20-24 cells for each condition; data are pooled from one experiment	n = 61-73 cells for each condition; data are pooled from three independent experiments
Figure 2i (previously 2g)	n = 27-37 cells for each condition; data are pooled from one experiment	n = 62-66 cells for each condition; data are pooled from three independent experiments
Figure 2k (previously 2i)	n = 17-22 for each condition; data are pooled from one experiment	n = 99 for each condition; data are pooled from three independent experiments
Figure 3h	n = 24 cells for both conditions; data are pooled from one experiment	n = 92 cells for both conditions; data are pooled from four independent experiments
Figure 5d	n = 26-30 cells for each condition; data are pooled from one experiment	n = 63 cells for each condition; data are pooled from three independent experiments

Figure 5f	n = 35-45 cells for each condition; data are pooled from one experiment	n = 80-83 cells for each condition; data are pooled from three independent experiments
Figure 6i	n = 51 cells for control and 31 cells for O/A; data are pooled from one experiment	n = 82 cells for control and 83 cells for O/A; data are pooled from three independent experiments
Figure 6l	n = 16-26 cells for each condition; data are pooled from one experiment	n = 69-81 cells for each condition; data are pooled from three independent experiments
Figure 7b	n = 28 cells for DMSO + O/A and 24 for BafA1 + O/A; data are pooled from one experiment	n = 83 cells for DMSO + O/A and 84 for BafA1 + O/A; data are pooled from three independent experiments
Figure 7d	n = 20-23 cells for each condition; data are pooled from one experiment	n = 88-91 cells for each condition; data are pooled from three independent experiments
Figure 7f	n = 27-29 cells for each condition; data are pooled from one experiment	n = 82-85 cells for each condition; data are pooled from three independent experiments
New Figure 7h	NA	n = 59 cells for WT + O/A and 54 for PI4KIIα KO + O/A; data are pooled from three independent experiments
New Figure 7j	NA	n = 69-75 cells for each condition; data are pooled from three independent experiments
Figure S7b	n = 26-38 cells for each condition; data are pooled from one experiment	n = 60 cells for each condition; data are pooled from three independent experiments
Figure S7d	n = 21-26 cells for each condition; data are pooled from one experiment	n = 56-71 cells for each condition; data are pooled from three independent experiments
New Figure S7h	NA	n = 92-96 cells for each condition; data are pooled from three independent experiments

We also updated the Methods section to provide additional details on the quantification methods.

2. In Figure 1e, they demonstrated that EGFP-GRAM-W progressively accumulates in TOM20-mSc-labeled mitochondria, yet the majority of cholesterol pools showed distinct localization patterns, what functional roles might these non-mitochondrial cholesterol pools play.

Reply: Thank you very much for this suggestion. In **Figure 1e**, the non-mitochondrial cholesterol pools correspond to the normal pool of cholesterol at the plasma membrane. We have added a label in the relevant images to indicate the plasma membrane and to avoid any confusion.

3. In Figure 2, genetic ablation of PI4P and OSBP would help determine whether O/A-induced cholesterol enrichment on lysosomal membranes depends on this transport pathway.

Reply: Thank you very much for these comments and suggestions. PI4P is a phosphoinositide, a key signalling phospholipid generated by the coordinated actions of specific lipid kinases and phosphatases. Therefore, genetic ablation of PI4P itself is not feasible. To circumvent this, we suppressed PI4P accumulation on lysosomal membranes by genetically knocking out

PI4KIIα, a lipid kinase responsible for the production of PI4P on lysosomal membranes (**Figures 2a and 2b**). Using this approach, PI4P accumulation on lysosomal membranes upon O/A treatment was significantly reduced compared with wild-type control cells (**Figures 2a and 2b**). This lead to impaired cholesterol delivery to lysosomal membranes, demonstrating the critical importance of PI4P generated by PI4KIIα in mediating cholesterol transport to lysosomes during mitophagy (**now Figures 2e and 2f in the revised manuscript**).

We also attempted OSBP knockout using CRISPR/Cas9-mediated gene editing but found that complete loss of OSBP is lethal in N/TERT cells. As an alternative approach, we pharmacologically inhibited OSBP function using the well-characterized chemical inhibitor OSW-1 (**now Figures 2h and 2i in the revised manuscript**). OSW-1 treatment markedly reduced cholesterol accumulation on lysosomal membranes upon O/A treatment, consistent with OSBP's role in mediating cholesterol delivery to lysosomes during mitophagy.

They should also verify PI4P levels regulated by OSBP.

Reply: Thank you very much for this suggestion. We performed additional experiments to assess the pool of PI4P on lysosomal membranes regulated by OSBP, using N/TERT cells stably expressing EGFP-GRAM-W (cholesterol marker), dTomato-ORP9-PH (PI4P marker), and LAMP1-miRFP703 (lysosomal marker). Upon treatment with OSW-1, a chemical inhibitor of OSBP, we observed significant increase in PI4P levels on lysosomal membranes (**Appendix Figure 3-1**). These results confirm that PI4P levels on lysosomal membranes are regulated by OSBP activity; inhibition of OSBP halts the counter-transport of cholesterol and PI4P at ER-lysosome contact sites, leading to PI4P accumulation on lysosomal membranes.

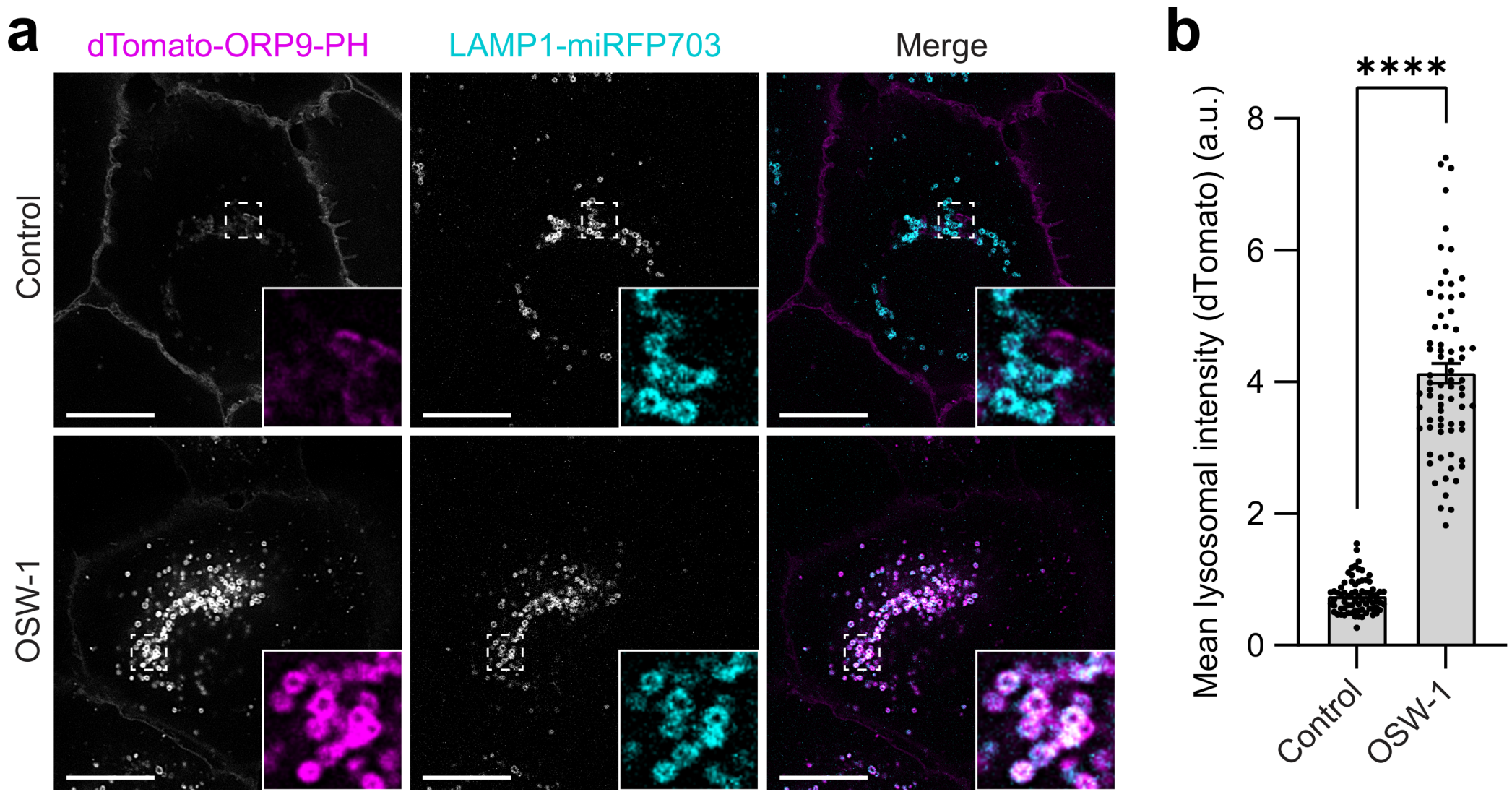

Appendix Figure 3-1
a. SDC-SIM images of N/TERT cells stably expressing EGFP-GRAM-W, dTomato-ORP9-PH, and LAMP1-miRFP703. Cells were treated with DMSO (Control), or 20 nM OSW-1 for 4 hours. Insets show at higher magnification the regions indicated by white dashed boxes. Scale bars, 10 µm. **b.** Quantification of dTomato-ORP9-PH signals on lysosomal membranes as shown in (a) (mean ± SEM, n = 72 cells for control and 73 cells for O/A; data are pooled from three independent experiments; Two-tailed unpaired t-test, ****$P < 0.0001$).

A comparative analysis of cholesterol and PI4P distribution between trans-Golgi and lysosomes, both pre- and post-OA treatment, would reveal potential trafficking dynamics.

Reply: Thank you very much for this suggestion. We performed a new set of experiments to examine cholesterol and PI4P levels at lysosomes and the trans-Golgi network (TGN) before and after O/A treatment in fixed cells. We observed a significant increase in cholesterol on

lysosomal membranes, labelled with the anti-LAMP1 antibody (albeit less prominent than in live-cell imaging, likely due to fixation affecting EGFP-GRAM-W binding) and on the TGN, labelled with the anti-TGN46 antibody. We also observed a significant increase in PI4P on lysosomal membranes, but not at the TGN (which already has high levels of PI4P at steady state), suggesting that lysosomes are the primary sites of additional PI4P generation during mitophagy. The increase of cholesterol levels in the TGN may reflect the additional delivery of ER cholesterol, whose biosynthesis is enhanced during mitophagy. These new results are presented in **Appendix Figure 3-2**.

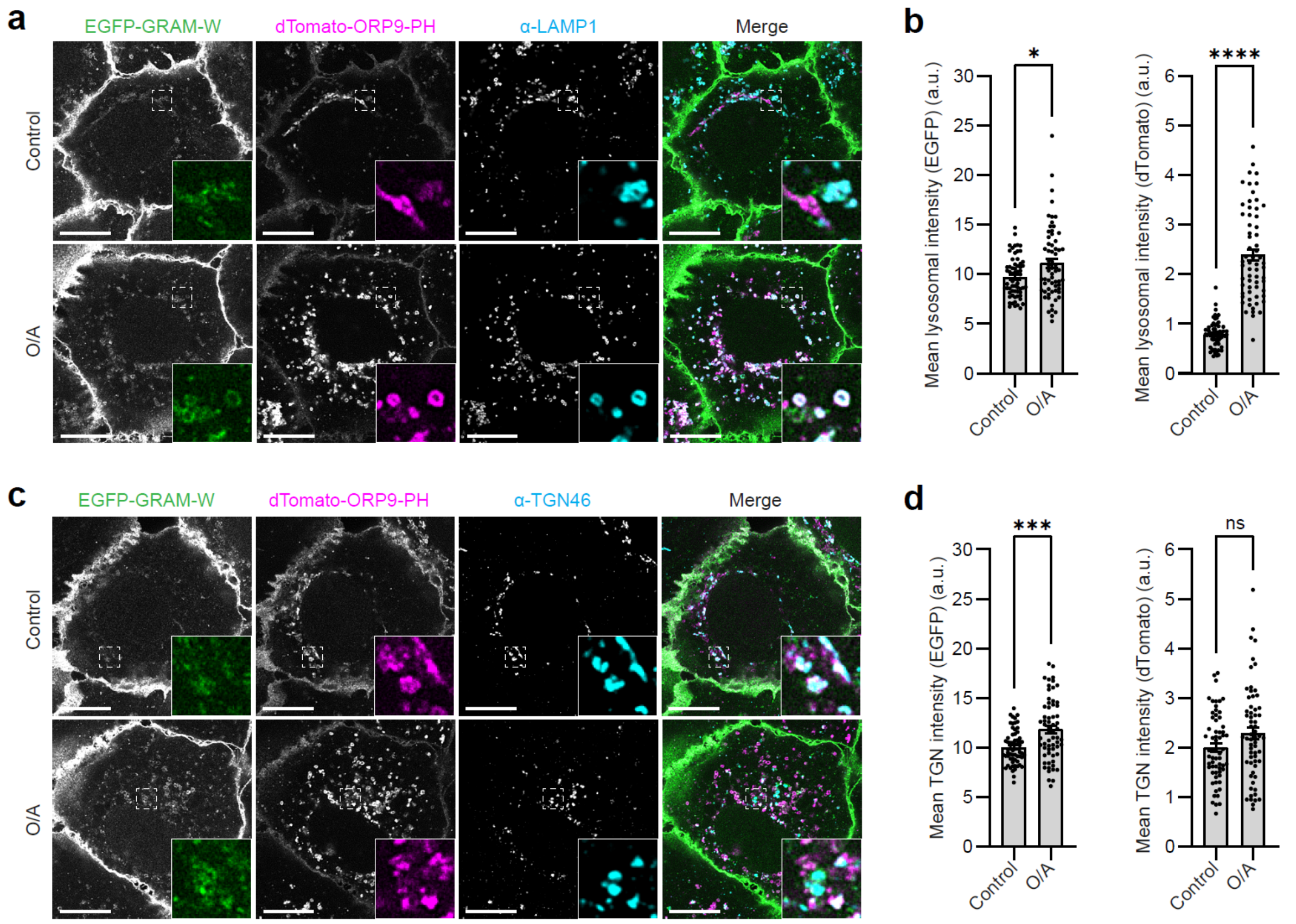

Appendix Figure 3-2
a. SDC-SIM images of N/TERT cells stably expressing EGFP-GRAM-W and dTomato-ORP9-PH. Cells were treated with ethanol (Control), or O/A for 2 hours, fixed and immunolabelled using antibodies against LAMP1. Insets show at higher magnification the regions indicated by white dashed boxes. Scale bars, 10 μm. **b.** Quantification of cholesterol (EGFP-GRAM-W) and PI4P (dTomato-ORP9-PH) signals on lysosomal membranes as shown in (a) (mean ± SEM, n = 58-61 cells for each condition; data are pooled from three independent experiments; Two-tailed unpaired t-test, *P=0.0425, ****P < 0.0001). **c.** SDC-SIM images of N/TERT cells stably expressing EGFP-GRAM-W and dTomato-ORP9-PH. Cells were treated with ethanol (Control), or O/A for 2 hours, fixed and immunolabelled using antibodies against TGN46. Insets show at higher magnification the regions indicated by white dashed boxes. Scale bars, 10 μm. **d.** Quantification of cholesterol (EGFP-GRAM-W) and PI4P (dTomato-ORP9-PH) signals on TGN membranes as shown in (c) (mean ± SEM, n = 63-64 cells for each condition; data are pooled from three independent experiments; Two-tailed unpaired t-test, ns, not significant, ***P= 0.0003).

4. In Figure 3, genetic knockout of SREBP-2 should be performed to validate its role in regulating both cholesterol content and PI4P expression on lysosomal membranes.

Reply: Thank you very much for this suggestion. We attempted to generate SREBP-2 knockout (KO) N/TERT cells using CRISPR/Cas9-mediated gene editing. However, complete loss of SREBP-2 was lethal, likely because N/TERT cells rely on cholesterol biosynthesis as their primary cholesterol source (PMID: 37880244). To achieve a similar effect, we used a SCAP inhibitor (PMID: 38330014), which traps SREBP-2 in the ER and prevents its cleavage in the Golgi, thereby mimicking SREBP-2 KO and reducing cholesterol biosynthesis in the ER.

Cells treated with the SCAP inhibitor UT-59 did not show cholesterol accumulation on lysosomal membranes upon O/A treatment, supporting that this additional pool of cholesterol on lysosomal membranes is derived from the ER (**Appendix Figure 3-3**). While SCAP inhibition alone did not affect PI4P levels on lysosomal membranes, O/A-induced increases in lysosomal PI4P were further enhanced by SCAP inhibition, likely due to inefficient countertransport of PI4P and cholesterol between lysosomes and the ER resulting from ER cholesterol depletion (**Appendix Figure 3-3**).

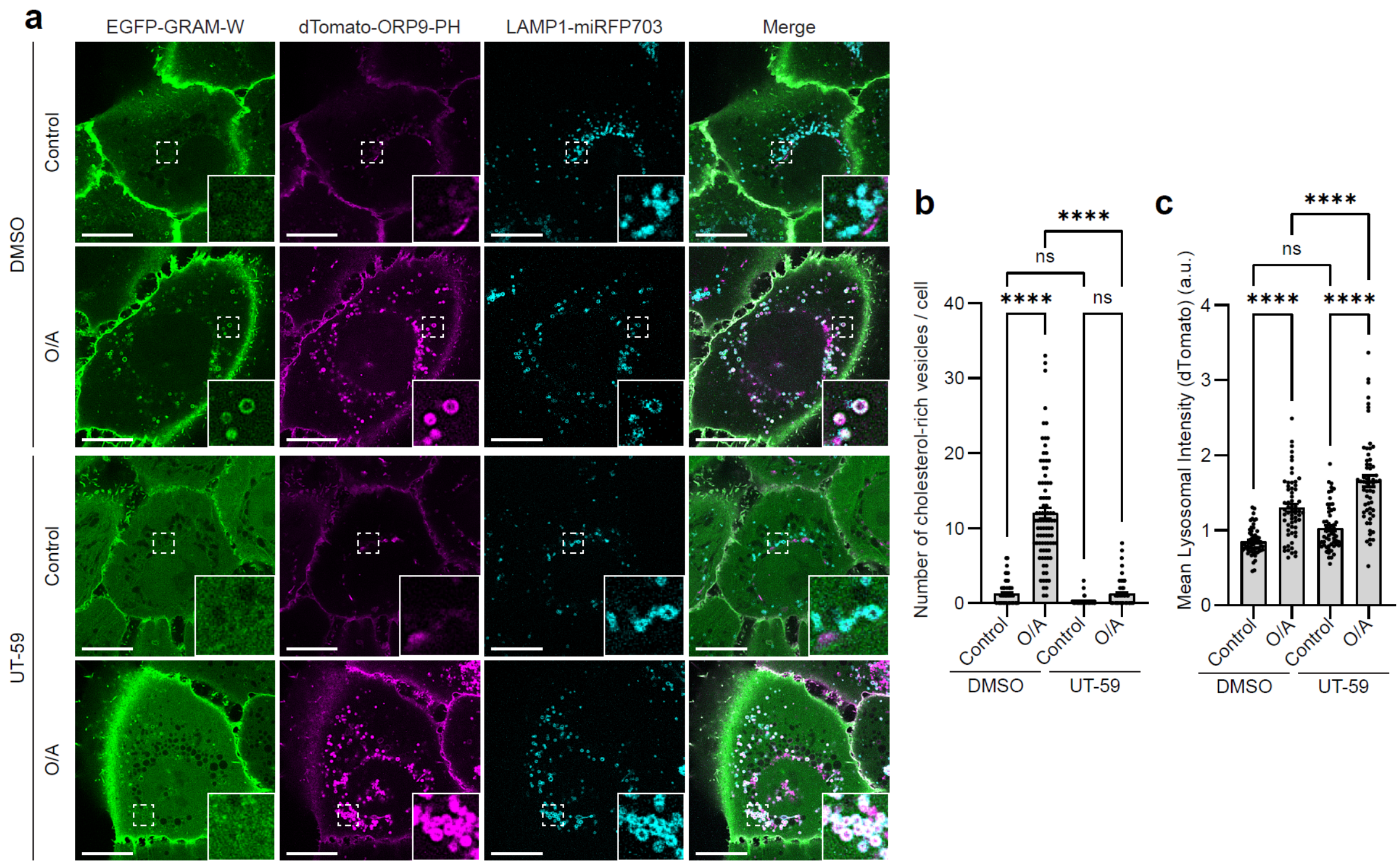

Appendix Figure 3-3
a. SDC-SIM images of N/TERT cells stably expressing EGFP-GRAM-W, dTomato-ORP9-PH, and LAMP1-miRFP703. Cells were pretreated with DMSO or UT-59 for 20 hours before treating with ethanol (Control) or O/A for 2 hours in the presence of DMSO or UT-59. Insets show at higher magnification the regions indicated by white dashed boxes. Scale bars, 10 µm. **b.** Quantification of the number of cholesterol-rich vesicles (detected by EGFP-GRAM-W) as shown in (a). (mean ± SEM, n = 87-90 cells for each condition; data are pooled from three independent experiments; Tukey's multiple comparisons test, ns, not significant, ****$P < 0.0001$). **c.** Quantification of dTomato-ORP9-PH signals on lysosomal membranes (labelled with LAMP1-miRFP703) as shown in (a) (mean ± SEM, n = 58-61 for each condition; data are pooled from three independent experiments; Tukey's multiple comparisons test, ns, not significant, ****$P < 0.0001$).

Whether PI4KIIα/PI4P KO inhibited SREBP-2 cleavage?

Reply: Thank you very much for this important question. We performed new experiments to examine SREBP-2 cleavage in PI4KIIα KO cells; however, we did not observe significant inhibition of SREBP-2 cleavage in these cells (**Appendix Figure 3-4**).

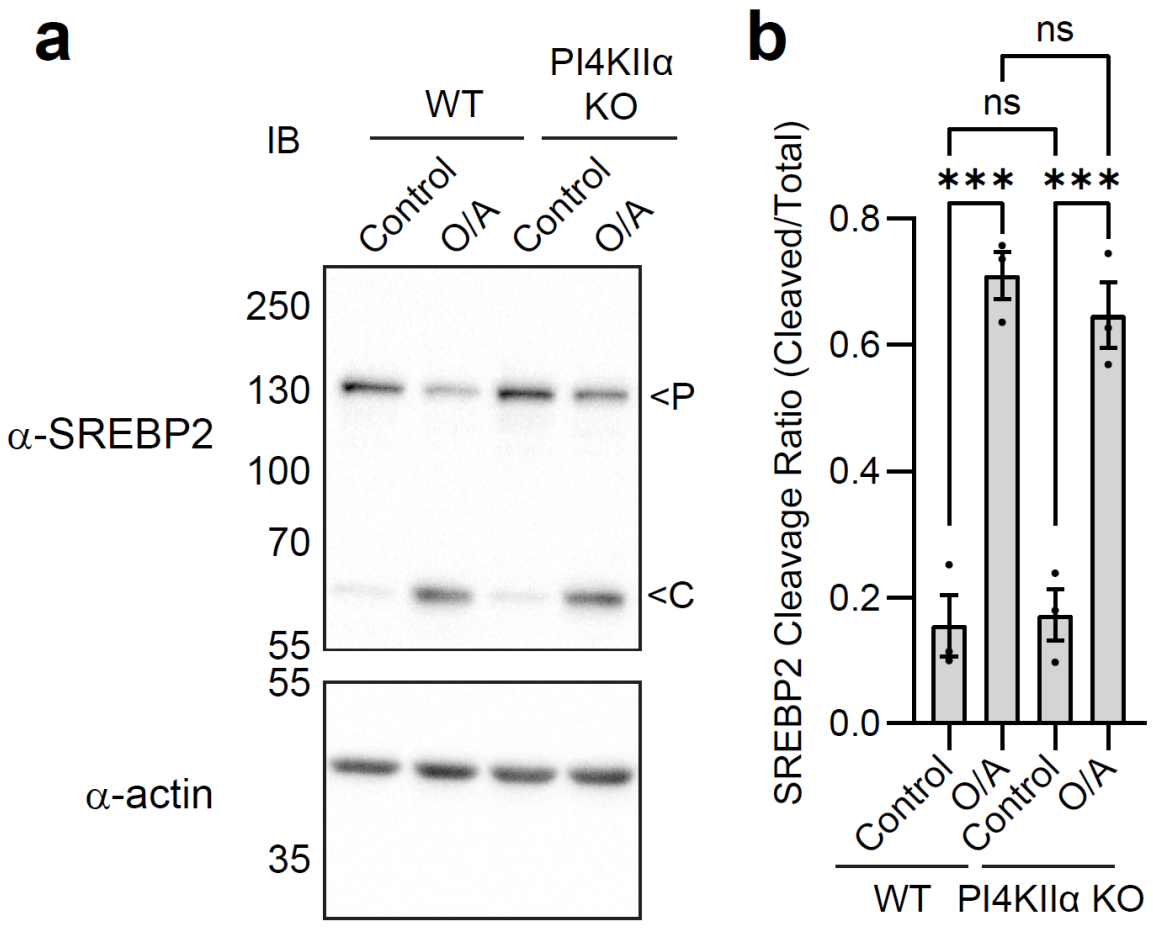

Appendix Figure 3-4
a. Lysates of N/TERT wild-type (WT) and PI4KIIα KO cells treated with ethanol (Control) or O/A for 4 hours were processed for SDS-PAGE and immunoblotting (IB) with antibodies against SREBP-2 and actin. Precursor (P) and cleaved (C) forms of SREBP-2 are indicated. **b.** Quantification of the ratio of cleaved SREBP-2 to total SREBP-2 as shown in (a) (mean ± SEM, n = 3 lysates from independent experiments for each condition; Tukey's multiple comparisons test, ***P = 0.0001 for control and O/A, ns, not significant.

In addition to the increase of cholesterol on lysosomal membranes, we also observed some accumulation of cholesterol at the plasma membrane, as assessed by EGFP-GRAM-W, following O/A treatment (**Figure 1a**). Interestingly, inhibition of OSBP via OSW-1 did not prevent this cholesterol accumulation (**Figure 2f**). This suggests that such cholesterol accumulation may be mediated by OSBP-independent mechanisms. This process may also contribute to the depletion of ER cholesterol, thereby inducing SREBP-2 cleavage even in the absence of PI4KIIα. While the characterization of such mechanisms is beyond the scope of the current study, we now discuss the potential relevance of this finding in the Discussion section as follows:

> *"In addition to the increase of cholesterol on lysosomal membranes, we also observed some increase of cholesterol at the PM, as assessed by EGFP-GRAM-W, following O/A treatment (Figure 1a). Notably, this increase in PM cholesterol was not suppressed by OSBP inhibition (Figure 2h), suggesting that such cholesterol accumulation at the PM is mediated by OSBP-independent mechanisms. This process may also contribute to the depletion of ER cholesterol and the subsequent activation of SREBP-2. Future studies will be required to elucidate the molecular mechanisms underlying this process."*

5. In Figure 4, they used a dTAG system to induced OSBP degradation, why not directly knockout the OSBP gene? Is there molecular evidence for direct interaction between OSBP and SREBP2?

Reply: Thank you very much for these questions. As mentioned above in reply to this reviewer's comment #3, we attempted OSBP knockout (KO) using CRISPR/Cas9-mediated gene editing but found that complete loss of OSBP is lethal in N/TERT cells. As an alternative approach, we employed the dTAG system to achieve rapid OSBP degradation, effectively mimicking the effects of OSBP KO. Regarding a potential interaction between OSBP and SREBP-2, there is no evidence supporting such an interaction, which is consistent with previous literature on the functions of these proteins.

6. In Figure 5, while lysosomal pH measurement provides one indicator of degradative capacity, additional assays would provide more comprehensive functional characterization.

Reply: Thank you very much for this comment. In addition to measuring lysosomal pH, we generated N/TERT cells stably overexpressing BFP-tagged Parkin and examined mitochondrial depolarization-dependent degradation of MTCO2 (COXII), an inner mitochondrial protein known to be targeted by mitophagy, using Western blotting, a well-established method in the field (PMID: 26266977; PMID: 27864321). Consistent with observations in other cell lines, Parkin overexpression dramatically enhanced O/A-dependent degradation of MTCO2 compared to control cells (**new Figures 5a and 5b**). To further test whether mitochondrial clearance depends on cholesterol, we examined O/A-dependent MTCO2 degradation in the presence of mevastatin to deplete cholesterol. Mevastatin treatment resulted in reduced MTCO2 degradation (**new Figures 5a and 5b**), providing additional evidence that mitochondrial clearance relies on cholesterol enrichment on degradative lysosomal membranes. We now replaced our previous data based on Magic Red with this new data to support the functional evidence of the role of cholesterol in mitochondrial degradation.

We have added the following sentences in the Results section to describe the new data:

> *"We first asked whether efficient degradation of depolarized mitochondria requires cholesterol. To address this, we generated N/TERT cells stably expressing BFP-tagged Parkin and examined mitophagy-dependent degradation of mitochondrial depolarization-dependent degradation of MTCO2 (COXII) by immunoblotting lysates from cells treated with O/A for 2 hours, followed by a 24-hour recovery, in the absence or presence of mevastatin to deplete cholesterol. In wild-type control cells, MTCO2 remained largely detectable, whereas BFP-Parkin-expressing cells showed robust MTCO2 degradation, consistent with previous reports[44,58] (Figure 5a). Notably, MTCO2 clearance was significantly reduced in cells treated with mevastatin, indicating that cholesterol is required for efficient degradation of damaged mitochondria (Figures 5a and 5b)."*

7. In Figure 6, the study demonstrates O/A-induced LD accumulation, but the functional relationships between these LDs and (1) lysosomal cholesterol/PI4P dynamics, and (2) SREBP2 activation remain unclear.

Reply: Thank you very much for these comments. In our model, the formation of lipid droplets (LDs) is a downstream consequence of lysosomal digestion of depolarized mitochondria. Cholesterol accumulation on lysosomes helps maintain their degradative capacity (**new Figures 5a and 5b; Figures 5c and 5d**), thereby promoting efficient LD formation.

To further support this model, we assessed LD formation in 1) PI4KIIα KO cells and 2) cells treated with a SCAP inhibitor, which mimics SREBP-2 KO (as described in response to this reviewer's comment #4). In both conditions, LD formation following O/A treatment was markedly suppressed. These results are consistent with the model in which PI4P-driven delivery of ER cholesterol, whose biosynthesis depends on SREBP-2 activity, to lysosomes is required for efficient LD formation following mitochondrial digestion. We have included these new data in **new Figures 7g and 7h; new Figures S7i and 7j**.

We have added the following sentences in the Results section to describe the new data (page 13):

> *"Finally, to test the role of PI4KIIα, and PI4P generated on lysosomal membranes by this kinase, in LD formation, we assessed LD formation in PI4KIIα KO cells. O/A-induced LD formation was markedly suppressed in PI4KIIα KO cells (Figures 7g and*

7h), supporting a model in which PI4P-driven delivery of ER cholesterol to lysosomes promotes efficient LD formation following mitochondrial digestion."

"Consistent with this result, treatment with UT-59, a SCAP inhibitor that blocks SREBP-2 cleavage and thereby inhibits cholesterol biosynthesis[69], similarly suppressed O/A-induced LD formation (Figures S7g and S7h)."

Importantly, does OSBP inhibition attenuate LD biogenesis, potentially linking these pathways.

Reply: Thank you very much for pointing this out. We performed additional experiments to investigate the effect of OSBP inhibition on O/A-induced LD formation using N/TERT cells stably expressing mCherry-ADRP. We found that the treatment of the cells with the OSBP inhibitor, OSW-1, significantly suppressed O/A-induced LD formation compared to control cells treated with DMSO (**new Figures 7i and 7j**). We also observed modest increase in the levels of LDs in cells treated with OSW-1 alone (**new Figures 7i and 7j**). This effect is consistent with the role of OSBP as a major lipid transfer protein that mediates cholesterol export from the ER, as OSBP inhibition likely leads to cholesterol accumulation in the ER, where excess cholesterol is esterified and stored in LDs.

Collectively, our new findings further support a role for OSBP in delivering cholesterol to degradative lysosomes during mitophagy, thereby maintaining lysosomal functional integrity required for efficient lipid recycling.

We have added the following sentences in the Results section to describe the new data (page 14):

"Consistent with a role of PI4P-dependent activation of OSBP in driving cholesterol delivery to lysosomal membranes, pharmacological inhibition of OSBP by OSW-1 also reduced O/A-induced LD formation (Figures 7i and 7j). Notably, treatment with OSW-1 alone modestly increased LD levels (Figures 7i and 7j). This effect is consistent with the role of OSBP as a major lipid transfer protein that mediates cholesterol efflux from the ER, as OSBP inhibition likely leads to cholesterol accumulation in the ER, where excess cholesterol is esterified and stored in LDs."

8. In Figure 7, they used NPC1 KO cells to verify LD formation, further confirming that the degradative function of lysosomes is a key requirement for LD biogenesis following mitochondrial depolarization, PI4P/OSBP/PI4KIIα-KO cells should also be used to verify LD formation.

Reply: Thank you very much for these suggestions. As described in reply to this reviewer's comment #3, PI4P is a phosphoinositide, a key signalling phospholipid generated by the coordinated actions of specific lipid kinases and phosphatases. Therefore, genetic ablation of PI4P itself is not feasible. To circumvent this, we suppressed PI4P accumulation on lysosomal membranes by genetically knocking out PI4KIIα, a lipid kinase responsible for the production of PI4P on lysosomal membranes (**Figures 2a and 2b**). Using this approach, PI4P accumulation on lysosomal membranes upon O/A treatment was significantly reduced compared with wild-type control cells (**Figures 2a and 2b**). To test the role of PI4KIIα, and PI4P generated on lysosomal membranes by this kinase, in LD formation, we assessed LD formation in PI4KIIα KO cells and found that O/A-induced LD formation was markedly suppressed (**new Figures 7g and 7h**), consistent with the model in which PI4P-driven delivery of ER cholesterol to lysosomes promotes efficient LD formation following mitochondrial digestion.

As mentioned above in reply to this reviewer's comment #3, we attempted OSBP knockout using CRISPR/Cas9-mediated gene editing but found that complete loss of OSBP is lethal in

N/TERT cells. As an alternative approach, we performed additional experiments to investigate the effect of pharmacological inhibition of OSBP on O/A-induced LD formation using N/TERT cells stably expressing mCherry-ADRP. We found that the treatment of the cells with the OSBP inhibitor, OSW-1, significantly suppressed O/A-induced LD formation compared to control cells treated with DMSO (**new Figures 7i and 7j**). We also observed modest increase in the levels of LDs in cells treated with OSW-1 alone (**new Figures 7i and 7j**). This effect is consistent with the role of OSBP as a major lipid transfer protein that mediates cholesterol export from the ER, as OSBP inhibition likely leads to cholesterol accumulation in the ER, where excess cholesterol is esterified and stored in LDs.

We have added the following sentences in the Results section to describe the new data (page 14):

> *"Finally, to test the role of PI4KIIα, and PI4P generated on lysosomal membranes by this kinase, in LD formation, we assessed LD formation in PI4KIIα KO cells. O/A-induced LD formation was markedly suppressed in PI4KIIα KO cells (Figures 7g and 7h), supporting a model in which PI4P-driven delivery of ER cholesterol to lysosomes promotes efficient LD formation following mitochondrial digestion."*

> *"Consistent with a role of PI4P-dependent activation of OSBP in driving cholesterol delivery to lysosomal membranes, pharmacological inhibition of OSBP by OSW-1 also reduced O/A-induced LD formation (Figures 7i and 7j). Notably, treatment with OSW-1 alone modestly increased LD levels (Figures 7i and 7j). This effect is consistent with the role of OSBP as a major lipid transfer protein that mediates cholesterol efflux from the ER, as OSBP inhibition likely leads to cholesterol accumulation in the ER, where excess cholesterol is esterified and stored in LDs."*

Reviewer #4 (Remarks to the Author):

I co-reviewed this manuscript with one of the reviewers who provided the listed reports. This is part of the Nature Communications initiative to facilitate training in peer review and to provide appropriate recognition for Early Career Researchers who co-review manuscripts.

We sincerely thank the reviewer for the constructive comments, which have greatly helped us improve the manuscript.

Nature Communications manuscript NCOMMS-25-49554A

REBUTTAL TO THE COMMENTS RAISED BY THE REVIEWERS

We thank the editor and the reviewers for the constructive comments and suggestions. We have carried out new experimentation to address all the concerns and made necessary changes to the texts. We have now:

1) Performed transient siRNA-mediated knockdowns of OSBP and SREBP-2 to provide orthogonal validation of our pharmacological findings (**new Figures S8a-e**).
2) Performed additional experiments to show that re-supplementing cholesterol following cholesterol depletion restored the efficient degradation of damaged mitochondria compared with the cholesterol-depleted condition, as assessed by the detection of MTCO2 via Western blotting (**new Figures 5a and 5b**).

A specific list of changes is indicated below, followed by a point-by-point rebuttal from page 2.

<u>The following data are newly included:</u>

Immunoblot analysis of mitochondrial degradation following mitophagy induction

- **Figures 5a and 5b:** Immunoblot analysis showing that re-supplementing cholesterol following cholesterol depletion restored the efficient degradation of damaged mitochondria compared with the cholesterol-depleted condition, as assessed by the detection of MTCO2.

siRNA-mediated knockdown of OSBP and SREBP-2

- **Supplementary Figure 8a:** Immunoblot analysis showing the efficient depletion of OSBP and SREBP-2, following siRNA treatment.
- **Supplementary Figures 8b and 8c:** Showing the suppression of O/A-induced cholesterol accumulation on lysosomal membranes in OSBP and SREBP-2 knockdown cells.
- **Supplementary Figures 8d and 8e:** Showing the suppression of O/A-induced lipid droplet formation in OSBP and SREBP-2 knockdown cells.

The following data are provided for the reviewers' reference only.

- **Appendix Figure 3-1:** Showing that suppression of lipid droplet formation by DGAT1 inhibition does not affect O/A-induced lysosomal cholesterol accumulation.

-Sentences from the reviewers' comments are *in italics*
-Our responses are in blue

Reviewer #1 (Remarks to the Author):

The authors have adequately addressed my key concerns raised during review, and I consider the manuscript suitable for publication.

Reply: We sincerely thank the reviewer for all the constructive comments and valuable suggestions, which have greatly improved the quality and clarity of our manuscript.

Reviewer #2 (Remarks to the Author):

Yang et al have submitted a revised manuscript on the role of cholesterol in maintaining lysosomal degradative capacity during mitophagy. In general the authors have substantially improved their paper with the addition of multiple novel datasets, based on the reviewers' comments. The novel data further strengthen the proposed working mechanism of cholesterol. I am very satisfied on how the authors addressed all critiques and improved their manuscript both qualitatively and quantitatively with these new data. I have only a few small remarks that the authors should comment or adjust in their manuscript, but which would otherwise not question my recommendation for publication.

Reply: We greatly appreciate the reviewer's positive assessment of our revised manuscript. Below, we provide our responses to the additional comments and remarks raised by the reviewer.

- In Fig. 1e (and suppl movie 1), an example is shown for the accumulation of chol in degradative lysosomal membranes. The authors state that chol accumulates from the moment mitophagy is initiated, but this is an example of a degradative lysosome that already contains Tomm20 staining at the beginning of O/A treatment (so, chol should be already on it). Is there no example where a degradative lyso becomes positive for tomm20 (does receives mito for degradation) and next accumulates chol?

Reply: Thank you very much for this suggestion. Despite our best efforts, we were unable to capture the early phase in which degradative lysosomes first become TOMM20-mScarlet-positive and subsequently EGFP-GRAM-W-positive, as prolonged imaging led to significant bleaching of both fluorescent signals. This was further complicated by the fact that most lysosomes already contained detectable TOMM20-mScarlet at steady state, likely reflecting basal mitophagy in our cell line.

- Related to Fig. 5e-f, the text states that 'additional treatment of cells with O/A or mevastatin dramatically enhanced lysosomal membrane rupture', but this doesn't differentiate for the protective role of chol in control cells with LLOME treatment. From the data it is clear that in control cells LLOME increased fragility in O/A treated cells but this was more dramatically when cells were depleted from cholesterol. The authors could adjust the text to highlight this difference.

Reply: Thank you very much for pointing this out. We have revised the following sentences in our revised manuscript:

Original sentences:

> *"Whereas LLOMe alone did not induce significant lysosomal membrane rupture in this condition, the additional treatment of cells with O/A or mevastatin dramatically enhanced lysosomal membrane rupture, suggesting that these lysosomal membranes were more fragile (Figures 5e and 5f)."*

Revised sentences:

> *"Whereas LLOMe treatment alone did not result in significant lysosomal membrane rupture, additional treatment with O/A induced substantial lysosomal membrane rupture, suggesting that lysosomal membranes become more fragile during mitophagy (Figures 5e and 5f). Notably, mevastatin treatment caused greater lysosomal membrane rupture, with no further increase observed upon combined treatment with O/A (Figures 5e and 5f).*

These results demonstrate that membrane cholesterol plays a protective role in maintaining lysosomal membrane integrity."

- In Fig. 7, the authors demonstrate that LD formation is reduced in O/A treated cells when SCAP is inhibited, in mevastatin treated cells, when PI4KIIa is knocked out, or following inhibition of OSBP by OSW-1, showing that chol is crucial for mitophagy-induced LD formation. However, when control cells are treated with OSW-1, this also resulted in increased LD levels: in this case, the authors argue that instead of storage of FAs, this is caused by chol accumulation in the ER where 'excess chol is esterified and stored in LDs'. If true, these LDs should be positive for a cholesterol marker (GRAM-W of filipin)?

Reply: Thank you very much for this comment. Excess cholesterol in the ER is converted into cholesterol esters before being sequestered into lipid droplets (LDs). GRAM-W detects unesterified cholesterol on cellular membranes that is exposed to the cytosol and therefore cannot detect cholesterol esters stored within the lumen of LDs (PMID: 37880244). Filipin likewise binds unesterified cholesterol and does not stain cholesteryl esters within LDs (PMID: 6202548). Therefore, the absence of GRAM-W and filipin signals at these LDs is expected.

And second, if a blockade in chol transport to lyso increases LDs, shouldn't that be the case as well for the other treatments under O/A conditions? Or is the flux of FAs more dominant compared to chol in these cases. Could the authors comment or clarify this further?

Reply: Thank you very much for this comment.

In addition to OSBP inhibition using its chemical inhibitor OSW-1, our manuscript includes three other manipulations that impair cholesterol delivery to lysosomes: cholesterol depletion via mevastatin, which inhibits HMGCR; cholesterol depletion through SCAP inhibition, which blocks SREBP-2 cleavage; and PI4KIIα knock-out (KO). Although all of these manipulations inhibit cholesterol transport to lysosomes, only OSW-1 treatment induces cholesterol accumulation in the ER, which promotes cholesterol esterification and LD formation. This effect is therefore specific to OSBP inhibition / OSW-1 treatment and is not a general consequence of impaired lysosomal cholesterol transport.

In mevastatin-treated and SCAP-inhibited cells, cholesterol biosynthesis is globally inhibited through inhibition of HMGCR or SREBP-2 cleavage, respectively. This results in reduced ER cholesterol levels; therefore, there is no enhancement of cholesterol ester-driven LD formation.

In PI4KIIα KO cells, lysosomal PI4P, the major driver of OSBP-mediated cholesterol transport at ER-lysosome contact sites, is specifically reduced, selectively impairing OSBP activity at these sites while preserving its function at other membrane contact sites, such as ER-Golgi contact sites. Accordingly, cholesterol does not accumulate in the ER in PI4KIIα KO cells, and cholesterol ester-driven LD formation is not expected in this condition. In contrast, OSBP inhibition by OSW-1 impairs OSBP activity at all contact sites, leading to ER cholesterol accumulation due to halted cholesterol export from the ER. This accumulation is likely followed by conversion into cholesterol esters, thereby resulting in increased LD formation.

Finally, in OSW-1-treated conditions, O/A treatment does not further increase lipid LD formation, likely because impaired degradation of damaged mitochondria (which result

from the absence of cholesterol accumulation in lysosomal membranes) reduces fatty acid (FA) flux required for LD formation.

- Ref doi: 10.3389/fnins.2022.900338 is a more updated review on MCSs in neurodegenerative diseases.

Reply: Thank you very much for this suggestion. We have cited this insightful review in our revised main text.

Reviewer #3 (Remarks to the Author):

Overall, the revised manuscript is rigorous, complete, and provides significant new insights. It is suitable for potential publication. I have four suggestions for further strengthening the study:

Reply: We greatly appreciate the reviewer's positive assessment of our revised manuscript. Below, we provide our responses to the comments and suggestions raised by the reviewer.

(1) The authors report that complete loss of OSBP, PI4P, and SREBP-2 is lethal. Would transient siRNA knockdown also be lethal? If viability is retained, such knockdowns could serve as orthogonal, genetically defined validation of the key pharmacological findings.

Reply: Thank you very much for this suggestion. We performed transient siRNA-mediated knockdowns of OSBP and SREBP-2 to provide orthogonal validation of our pharmacological findings. Although complete loss of OSBP or SREBP-2 compromises cell viability, we identified a viable window to assess cholesterol distribution and LD formation. Our new results have been added to the manuscript as **new Figure S8**, which are consistent with and support our pharmacological data.

- OSBP knockdown (**new Figures S8a–f**): siRNA-mediated depletion of OSBP suppressed lysosomal cholesterol accumulation induced by O/A treatment. Notably, it induced a basal increase in LD formation while blunting any further increase in LDs upon O/A treatment. These results are consistent with those obtained using the OSBP inhibitor OSW-1 (**Figures 2h, 2i, 7i, and 7j**).
- SREBP-2 knockdown (**new Figures S8b–f**): siRNA-mediated depletion of SREBP-2 suppressed both lysosomal cholesterol accumulation and LD formation induced by O/A treatment. These results are consistent with those obtained using mevastatin, an HMGCR inhibitor, and UT-59, a SCAP inhibitor (**Figures 3g, 3h, 7e, 7f, S7g, and S7h**).

Together with our results on PI4KIIα knockout, which suppresses both lysosomal cholesterol accumulation and LD formation induced by O/A treatment through inhibition of lysosomal PI4P accumulation (**Figures 2e, 2f, 7g and 7h**), these new experiments provide validation of our key findings and further strengthen the conclusion of our study.

We have included the following sentences to the result section of the revised manuscript:

> *"As a complementary approach, SREBP-2 expression was transiently reduced using RNA interference (RNAi) (Figure S8a). Treatment of cells with small interfering RNA (siRNA) targeting SREBP-2 for 72 hours results in significant suppression of both lysosomal cholesterol accumulation and LD formation induced by O/A treatment (Figures S8b-e). These results further support the crucial role of SREBP-2-mediated cholesterol biosynthesis in mitophagy-induced LD formation."*
>
> *"Similar results were obtained using siRNA targeting OSBP (Figures S8a-e), supporting the crucial role of OSBP-mediated cholesterol transport in this process."*

(2) The authors show cholesterol depletion impairs lysosomal function. Restoring

cellular cholesterol levels could test whether enhanced cholesterol availability rescues lysosomal degradative capacity.

Reply: Thank you very much for this suggestion. In response, we conducted additional experiments that included a cholesterol re-supplementation condition, in which mevastatin was removed and methyl-β-cyclodextrin-loaded cholesterol (MCD/Chol) was added to deliver cholesterol to cells. We also optimized our experimental protocol by increasing the mevastatin concentration from 5 to 25 μM and harvesting lysates 18 hours after O/A treatment instead of 24 hours. These modifications enabled a more robust distinction among the control, mevastatin-treated, and cholesterol re-supplementation conditions. As shown in the **new Figures 5a and 5b**, cholesterol re-supplementation following cholesterol depletion restored the efficient degradation of damaged mitochondria relative to the cholesterol-depleted condition, as assessed by MTCO2 levels measured via Western blotting. These results provide further support for the role of cholesterol in maintaining the degradative capacity of lysosomes during mitophagy.

We have revised the following sentences in the result section (page 11):

Original sentences:

> *"Notably, MTCO2 clearance was significantly reduced in cells treated with mevastatin, indicating that cholesterol is required for efficient degradation of damaged mitochondria (Figures 5a and 5b)."*

Revised sentences:

> *"Notably, MTCO2 clearance was significantly reduced in cells treated with mevastatin (Figures 5a and 5b). Furthermore, MTCO2 clearance was restored by cholesterol re-supplementation through the removal of mevastatin and the addition of MCD/Chol, following 24 hours of mevastatin treatment (Figures 5a and 5b). These results demonstrate that cholesterol is required for efficient degradation of damaged mitochondria."*

(3) The functional role of LD formation during mitophagy remains incompletely defined. Does LD accumulation act as a regulatory node—for instance, by sequestering excess cholesterol and thereby attenuating lysosomal cholesterol overload—or does it represent a passive, stoichiometric sink? Clarifying whether LDs exert active feedback on lysosomal cholesterol dynamics would critically inform their proposed role in modulating the mitophagy–lysosome axis.

Reply: We would like to clarify that cells undergoing mitophagy exhibit a marked accumulation of triacylglycerol (TAG), as detected by lipidomics (**Figure 6g**). Inhibition of DGAT1, the enzyme responsible for TAG synthesis, strongly suppresses the formation of mitophagy-induced lipid droplets (LDs). Furthermore, impairment of the degradative capacity of lysosomes similarly reduces LD formation. Together, these results indicate that LD biogenesis occurs downstream of the efficient degradation of damaged mitochondria by lysosomes. As discussed in our manuscript, we propose that LD formation serves as a mechanism to sequester excess lipids, including TAG, generated during mitochondrial degradation and / or to facilitate their subsequent recycling.

We examined whether inhibition of LD formation via DGAT1 inhibition affects cholesterol accumulation on lysosomal membranes during O/A-induced mitophagy and found that DGAT1 inhibition does not alter lysosomal cholesterol accumulation (**new Appendix Figure 3-1**). This new result suggests that LD formation is not required for lysosomal cholesterol accumulation under these conditions and further supports the notion that LD formation occurs downstream of mitophagy. Therefore, our data do not support a model in which LDs exert an active feedback role in regulating lysosomal cholesterol dynamics.

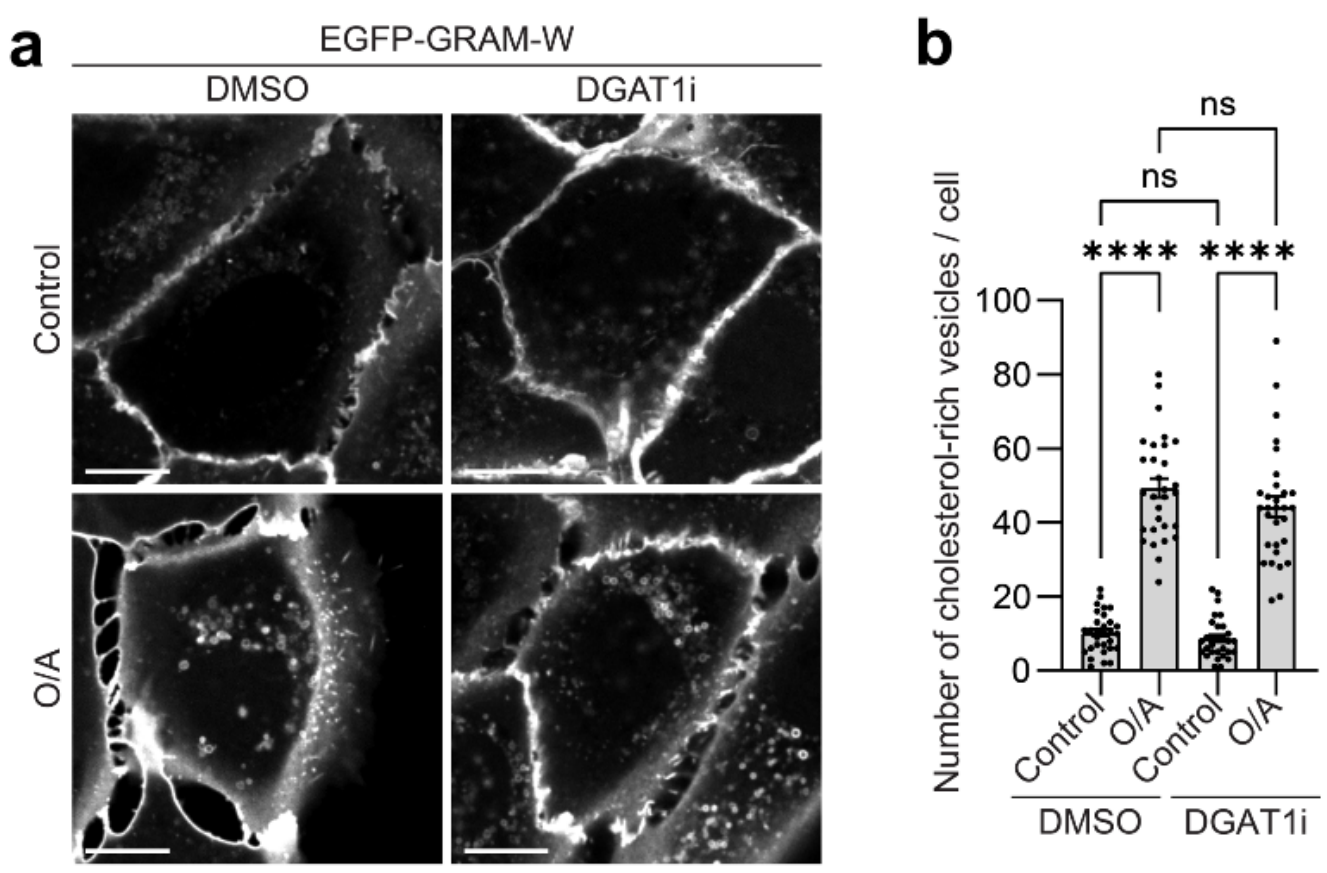

Appendix Figure 3-1

a. Confocal images of live N/TERT cells expressing EGFP-GRAM-W that were pre-treated with DMSO or 20 μM T863 (DGAT1i) for 2 hours and then treated with ethanol (Control) or O/A for 2 hours, washed with PBS, and recovered in KSFM for 24 hours. Scale bars, 10 μm. **b**. Quantification of the number of cholesterol-rich vesicles (detected by EGFP-GRAM-W) as shown in (a). (mean ± SEM, n = 30 cells for each condition; data are pooled from four independent experiments; Tukey's multiple comparisons test, ns, not significant, ****P<0.0001).

(4) Direct visualization and tracking of lipid trafficking would substantially strengthen the proposed model of inter-organellar lipid recycling. Specifically, pulse-labeling of mitochondrial membrane lipids could enable quantitative assessment of their transfer to lysosomes and subsequent incorporation into LDs. Similarly, ER-localized cholesterol could be selectively labeled to trace its mobilization to lysosomal membranes. Such approaches would provide definitive, spatially resolved evidence for the hypothesized lipid flux pathway.

Reply: We thank the reviewer for this insightful suggestion. We agree that directly tracking the transfer of mitochondria-derived lipids to lysosomes and their subsequent transfer to LDs would further strengthen our model. However, such experiments remain technically challenging. Most cellular lipids are synthesized in the ER and subsequently distributed to other organelles, including mitochondria and lysosomes, through complex trafficking pathways involving both vesicular and non-vesicular transport. While metabolic or pulse-labeling approaches (such as radiolabeled or stable isotope tracing) can effectively monitor lipid flux, they do not provide sufficient spatial resolution to distinguish the origin of lipids once they redistribute across multiple compartments. Consequently, when pulse-labeled lipids are detected in lysosomes, it is difficult to unambiguously determine whether they are derived from mitochondria or from other sources such as the ER.

In principle, mitochondria-specific labeling strategies, such as bifunctional, photoactivatable, or "caged" lipids, could offer improved spatial control. However, these approaches remain technically demanding, often requiring custom synthesis and careful validation, and have thus far been applied only in limited contexts to study lipid dynamics (e.g., PMID: 40770077, PMID: 40836094, PMID: 35654841). To our knowledge, their application to directly track lipid transfer from mitochondria to lysosomes, or from lysosomes to LDs, has not yet been robustly established in mammalian cells.

Regarding the tracking of ER-derived cholesterol, we previously showed that keratinocytes rely heavily on de novo cholesterol synthesis in the ER to maintain cellular cholesterol levels (PMID: 37880244), consistent with prior studies (PMID: 6192180, PMID: 2443512). Thus, cholesterol detected by GRAM-W and other cholesterol probes predominantly reflects ER-derived cholesterol in these cells, one of the reasons why we used keratinocytes as our model cells in this study, as stated in the results (page 5).

Given these technical limitations, we have taken a complementary approach by combining imaging, lipidomics, and both genetic and chemical perturbations, allowing us to support our model through multiple independent lines of evidence. We have clarified this point in the revised manuscript and highlighted direct tracking of mitochondria-derived lipids as an important direction for future studies.

We have included the following sentences to the discussion (page 17):

> *"In the future, it would be informative to track the movement of mitochondria-derived lipids during mitochondrial degradation using organelle-specific labelling approaches to gain further insights into the mechanisms of lipid recycling and/or storage"*

Reviewer #4 (Remarks to the Author):

I co-reviewed this manuscript with one of the reviewers who provided the listed reports. This is part of the Nature Communications initiative to facilitate training in peer review and to provide appropriate recognition for Early Career Researchers who co-review manuscripts.

Reply: Thank you very much for all the constructive feedback to further improve our manuscript.
